# eEF1G Orchestrates Translation to Ensure Meiotic Progression in Transcriptionally Quiescent Spermatocytes

**DOI:** 10.1002/advs.76264

**Published:** 2026-07-01

**Authors:** Jianze Xu, Yuwei Hu, Xukun Lu, Yuling Cai, Tongtong Li, Ming Gao, Jinlong Ma, Yuan Gao, Shangming Liu, Zi‐Jiang Chen, Jing Meng, Hongbin Liu, Xiaohua Jiang

**Affiliations:** ^1^ State Key Laboratory of Reproductive Medicine and Offspring Health, Center for Reproductive Medicine, Institute of Women, Children and Reproductive Health Shandong University Shandong P. R. China; ^2^ National Research Center for Assisted Reproductive Technology and Reproductive Genetics Shandong University Jinan Shandong P. R. China; ^3^ Key Laboratory of Reproductive Endocrinology, Ministry of Education Shandong University Jinan Shandong P. R. China; ^4^ Shandong Technology Innovation Center for Reproductive Health Jinan Shandong P. R. China; ^5^ Shandong Provincial Clinical Research Center for Reproductive Health Jinan Shandong P. R. China; ^6^ Shandong Key Laboratory of Reproductive Research and Birth Defect Prevention Jinan Shandong P. R. China; ^7^ School of Basic Medical Sciences Shandong University Jinan Shandong P. R. China; ^8^ Jinan Maternal and Child Health Care Hospital Jinan Shandong P. R. China; ^9^ Center for Reproduction and Genetics, Department of Obstetrics and Gynecology, The First Affiliated Hospital of USTC, Division of Life Sciences and Medicine University of Science and Technology of China Hefei Anhui P. R. China

**Keywords:** eukaryotic translation elongation factor 1, homologous recombination, male infertility, meiosis, translation elongation

## Abstract

During meiotic prophase I, mammalian spermatocytes must synthesize large amounts of recombination and synapsis proteins despite global transcriptional suppression at the leptotene/zygotene (L/Z) stages. Here, we identify eukaryotic translation elongation factor 1 gamma (eEF1G), highly expressed in spermatogenic cells, as a factor essential for sustaining translation during this transcriptionally quiescent period. Germ cell–specific *Eef1g* knockout causes complete male infertility due to zygotene arrest, characterized by defects in recombination intermediate stabilization and synapsis. Mechanistically, eEF1G associates with ribosomal proteins, and ribosome profiling reveals increased ribosome occupancy on specific meiotic transcripts in *Eef1g*‐deficient spermatocytes. Quantitative proteomics further reveals selective depletion of synapsis‐related (e.g., SYCP1, SYCE1) and recombination‐related proteins (e.g., MSH4, TEX11). Together, these findings demonstrate that eEF1G is required to maintain efficient protein production during the transcriptionally quiescent leptotene/zygotene stages, thereby supporting proper meiotic progression in mammalian spermatocytes.

## Graphical abstract

eEF1G supports translation elongation of meiotic mRNAs in transcriptionally quiescent leptotene and zygotene spermatocytes. Its depletion in germ cells causes meiotic arrest at the zygotene stage, with defective homologous synapsis and unstable recombination intermediates. Thus, eEF1G‐dependent translation elongation is essential for male meiosis and fertility.

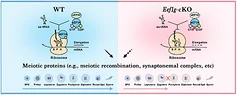

## 1 Introduction

Meiosis, the cornerstone of sexual reproduction, ensures genetic diversity and genome stability through a series of tightly orchestrated chromosomal events: programmed DNA double‐strand break (DSB) formation [1, 2, 3, 4], synaptonemal complex (SC)‐mediated homologous chromosome synapsis [5, 6], and homologous recombination (HR) [7, 8]. During early prophase I (leptotene to zygotene stages), spermatocytes face a fundamental paradox: they must produce an extensive vast repertoire of DSB repair proteins (e.g., MEIOB, HSF2BP, RAD51, and DMC1) and synapsis‐related factors (SYCP1, SYCE1, TEX12 and SIX6OS1) [6, 9, 10, 11, 12, 13] precisely, when transcriptional activity is globally suppressed to near‐undetectable levels at the leptotene and zygotene stages [14, 15, 16]. Unable to rely on new transcription, these cells must meet stage‐specific protein demands entirely through translation of pre‐stored mRNAs. Yet the molecular factors enabling this translational adaptation remain poorly understood, posing a fundamental question in reproductive biology: how do transcriptionally quiescent spermatocytes mobilize pre‐existing transcripts to drive the rapid protein synthesis required for meiotic completion?

Translation converts mRNA‐encoded genetic information into functional proteins through three mechanistic stages: initiation, elongation, and termination [17, 18]. During initiation, the small ribosomal subunit, together with initiation factors (e.g., eIF2, eIF4E), binds to the 5′ end of mRNA and scans for the start codon, and is joined by the large ribosomal subunit to form a translationally competent ribosome [19]. During elongation, ribosomes sequentially add amino acids to the nascent peptide chain in a conserved cycle guided by the codon template on the mRNA [17]. Each cycle comprises three core steps: decoding, peptide bond formation, and translocation—all catalyzed by the ribosome with critical assistance from essential elongation factors [20, 21]. In the decoding step, a cognate aminoacyl‐tRNA is selectively recruited and positioned at the ribosomal A‐site, a process mediated by the ribosomal decoding center in coordination with elongation factor eEF1A, eEF1B2, eEF1D, and eEF1G [22, 23, 24]. Subsequently, peptide bond formation is catalyzed by the ribosome's peptidyl transferase center, linking the nascent peptide chain to the amino acid carried by the incoming aminoacyl‐tRNA. Finally, elongation factor eEF2 facilitates translocation of the ribosome along the mRNA, shifting it one codon toward the 3’ end to enable the next round of elongation. Finally, stop codon recognition by release factors eRF1 and eRF3 triggers polypeptide release and ribosome recycling [25].

Although translation control during gametogenesis has been classically studied at the level of initiation or in terms of mRNA storage and decay, evidence is mounting that translation elongation constitutes an equally important regulatory node [26, 27, 28], particularly in cell types with high translational demand [29], such as transcriptionally quiescent early meiotic spermatocytes or elongating spermatids. Translation elongation depends on the coordinated action of eukaryotic elongation factors (eEFs), among which the eEF1 complex mediates the delivery of aminoacyl‐tRNAs to the ribosomal A‐site [30]. The subunit eEF1G serves as a structural scaffolding critical for eEF1B complex stability and efficient guanine nucleotide exchange, thereby promoting translation elongation [24, 28]. Notably, germ cells exhibit distinct translation regulation compared to somatic cells. Unlike somatic cells that rely primarily on transcription initiation and have continuous mRNA supply, germ cells depend on post‐transcriptional regulation and efficient translation elongation from stored transcripts [29, 31]. Interestingly, recent studies have linked elongation factors to germ cell‐specific functions: eEF1B2 regulates the proliferation of human spermatogonial stem cells via TAF4B [32], while eEF1A1b variants cause spermatogenesis arrest in male tilapia [33]. However, the function of eEF1G in mammalian meiosis remains entirely unexplored.

In this study, we show that eEF1G is highly expressed in spermatogenic cells, particularly during early meiotic stages. Germline‐specific *Eef1g* knockout males show infertility due to meiotic arrest at prophase I, accompanied by unresolved DNA double‐strand breaks, impaired homologous chromosome synapsis, and defective meiotic recombination. Consistent with a role in translational maintenance, eEF1G associates with ribosomes, and its loss reduces translation output during the transcriptionally quiescent leptotene/zygotene stages. These results identify eEF1G as a critical factor coupling translation capacity to the protein synthesis demands of early meiosis.

## 2 Results

### 2.1 eEF1G Is Highly Expressed in Spermatogenic Cells

**FIGURE 1:**
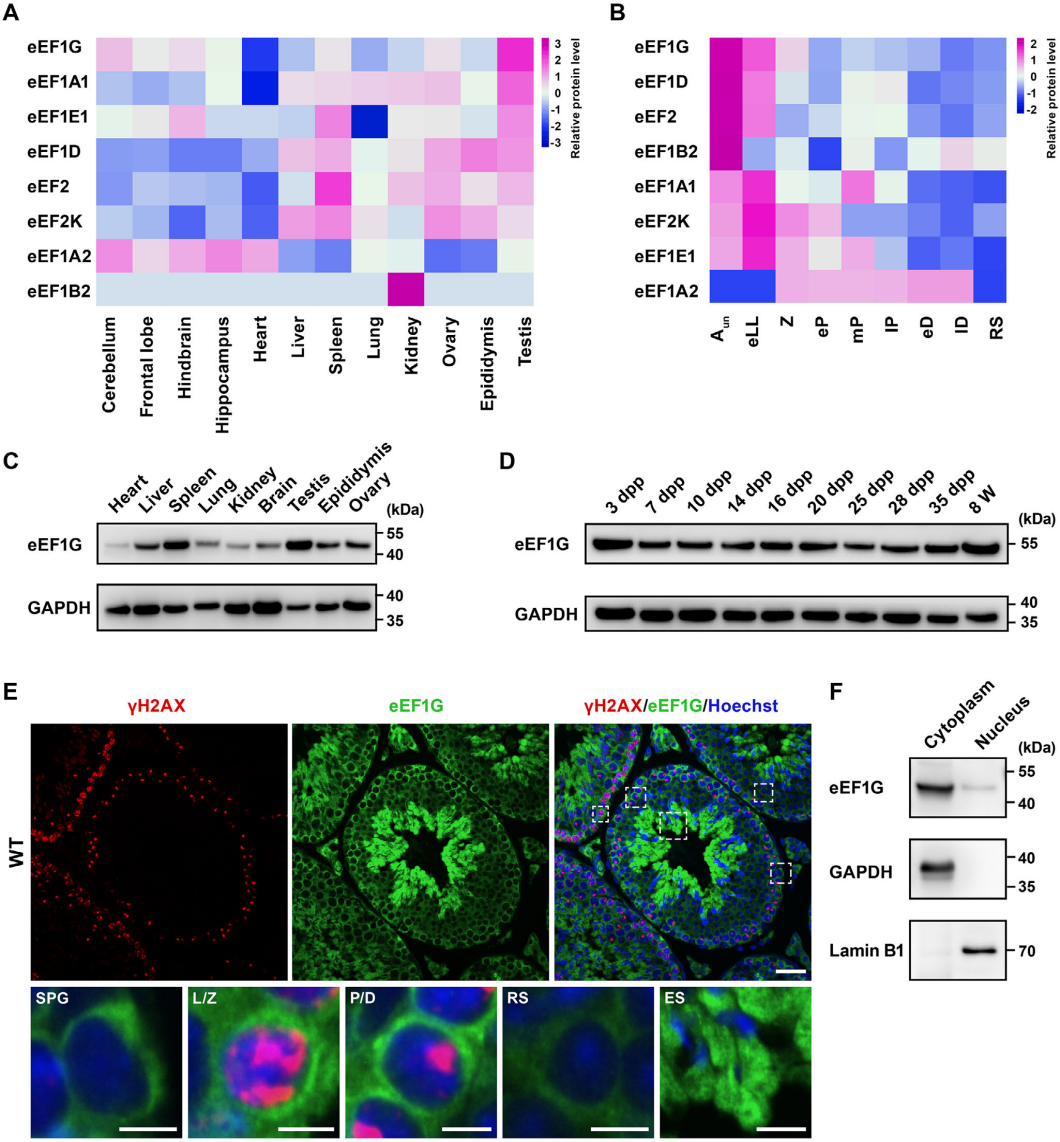
eEF1G displays a dynamic expression pattern during spermatogenesis. (A) Heatmap shows the protein expression profiles of eukaryotic translation elongation factors (EEFs) in mouse tissues (data from Giansanti et al. [35]). The color scale represents row‐wise z‐score–normalized protein abundance across tissues. (B) Heatmap shows the protein expression profiles of subunits of eukaryotic translation elongation factors (EEFs) during different stages of mouse spermatogenesis identified by proteomics (data from Fang et al. [36]). The color scale represents row‐wise z‐score–normalized protein abundance across developmental stages. A_un_, type A undifferentiated spermatogonia; eLL, early leptotene and leptotene; Z, zygotene; eP, early pachytene; mP, middle pachytene; lP, late pachytene; eD, early diplotene; lD, late diplotene; RS, round spermatids. (C) Western blot showing eEF1G protein expression in various organs in wild‐type adult mice. GAPDH served as a loading control. (D) Western blot showing eEF1G protein levels in wild‐type developing mouse testes. GAPDH served as a loading control. (E) Immunofluorescence staining of eEF1G and γH2AX in WT mouse testis cross‐sections. 50 µm (main panel) and 5 µm (insets). SPG, spermatogonia; L/Z, leptotene/zygotene; P/D, pachytene/diplotene; RS, round spermatid; ES, elongated spermatid. (F) Western blot analysis of eEF1G in the nucleus and cytoplasm of spermatogenic cells from 28 dpp testes. Lamin B1 and GAPDH were used to assess the separation efficiency of nuclear and cytoplasmic proteins, respectively.

To identify translation elongation factors that might be functionally relevant to meiosis, we surveyed eight proteins annotated to participate in translation elongation or its regulation, including eEF1A1, eEF1A2, eEF1B2, eEF1D, eEF1E1, eEF1G, eEF2, and eEF2K [17, 34]. Comprehensive analysis of mass spectrometry‐based proteomics data from mouse tissues [35] and spermatogenic cells [36] revealed that eEF1G displayed pronounced testicular enrichment (Figure 1A). Notably, it was more enriched in early meiotic prophase I cells, especially the leptotene and zygotene spermatocytes (Figure 1B and Figure S1A,B), the stages at which chromosomes undergo pairing, synapsis, and recombination. Western blot analyses demonstrated that eEF1G expression levels were relatively high in mouse testes (Figure 1C,D). Immunofluorescent staining showed that eEF1G can be detected in spermatogonia, spermatocytes, and round/elongating spermatids in adult mouse testes (Figure 1E). Notably, eEF1G exhibits a cytoplasmic localization in spermatogenic cells. To further validate the subcellular localization pattern of eEF1G protein, we prepared cytosolic and nuclear fractions from testes of mice at 28 days postpartum (dpp) for Western blot analysis. We confirmed that eEF1G is predominantly expressed in the cytoplasm of spermatogenic cells (Figure 1F).

### 2.2 eEF1G Deletion in Germ Cells Casuses Spermatogenic Impairment and Male Infertility

**FIGURE 2:**
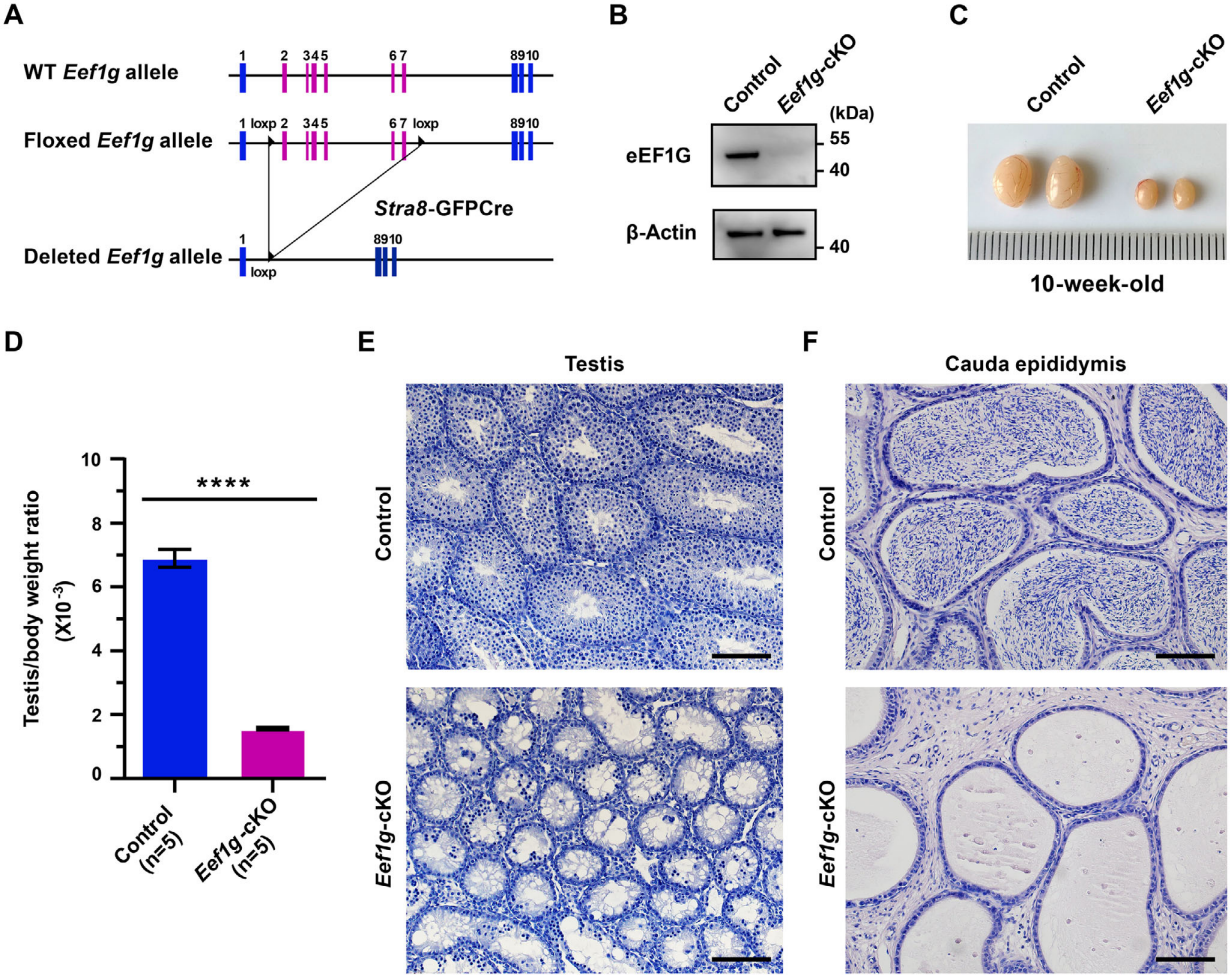
Deletion of *Eef1g* in spermatogenic cells leads to massive germ cell loss and infertility in male mice. (A) Schematic diagram of the targeting strategy used to generate a floxed *Eef1g* allele by the CRISPR/Cas9 gene editing technique. Deletion of *Eef1g* in germ cells using *Stra8*‐GFPCre. (B) Representative Western blot confirming the absence of eEF1G protein in leptotene and zygotene spermatocytes of 12 dpp *Eef1g*‐cKO mice and littermate controls. β‐Actin was used as a loading control. (C) Comparison of testis size between 10‐week‐old *Eef1g*‐cKO male mice and littermate controls. (D) Testis to body weight ratios of 10‐week‐old *Eef1g*‐cKO male mice and littermate controls. Data are presented as mean ± SEMs (n = 5 per group). Student's *t*‐test; ^****^ *p* < 0.0001. (E) Hematoxylin staining of testes from adult *Eef1g*‐cKO and control mice. Scale bars, 50 µm. (F) Hematoxylin staining of cauda epididymides from adult *Eef1g*‐cKO and control mice. Scale bars, 50 µm.

To investigate the physiological role of eEF1G in spermatogenesis, we generated a mouse model in which exons 2–7 of *Eef1g* were specifically deleted in male germ cells by using *Stra8*‐GFPCre (Figure 2A), which is expressed from 3 dpp and induces recombination starting from type A1 spermatogonia [37]. Western blot analyses of FACS‐enriched leptotene and zygotene spermatocytes confirmed a marked reduction in eEF1G protein (Figure 2B). Furthermore, immunofluorescence analyses verified the absence of eEF1G expression in spermatocytes from *Eef1g*‐cKO male mice (Figure S2A,B).

*Eef1g*‐cKO adult male mice were infertile and exhibited remarkably smaller testes compared with their age‐matched control littermates (Figure 2C,D). The vast majority of seminiferous tubules in *Eef1g*‐cKO mice exhibited obvious abnormalities, including disorganized tubular architecture and a substantial reduction in the germ cells (Figure 2E). Consistently, no sperm were found in the cauda epididymides in adult *Eef1g*‐cKO mice (Figure 2F). TdT‐mediated dUTP nick end labeling (TUNEL) staining of testis sections from *Eef1g*‐cKO mice revealed that both the proportion of seminiferous tubules containing apoptotic cells and the number of apoptotic germ cells per tubule were significantly increased (Figure S3A–C). These results indicate that a large number of germ cells are eliminated before the completion of spermatogenesis, which results in smaller testes and spermatogenesis arrest and thus infertility in *Eef1g*‐cKO mice.

### 2.3 eEF1G Is Essential for Meiotic Progression

**FIGURE 3:**
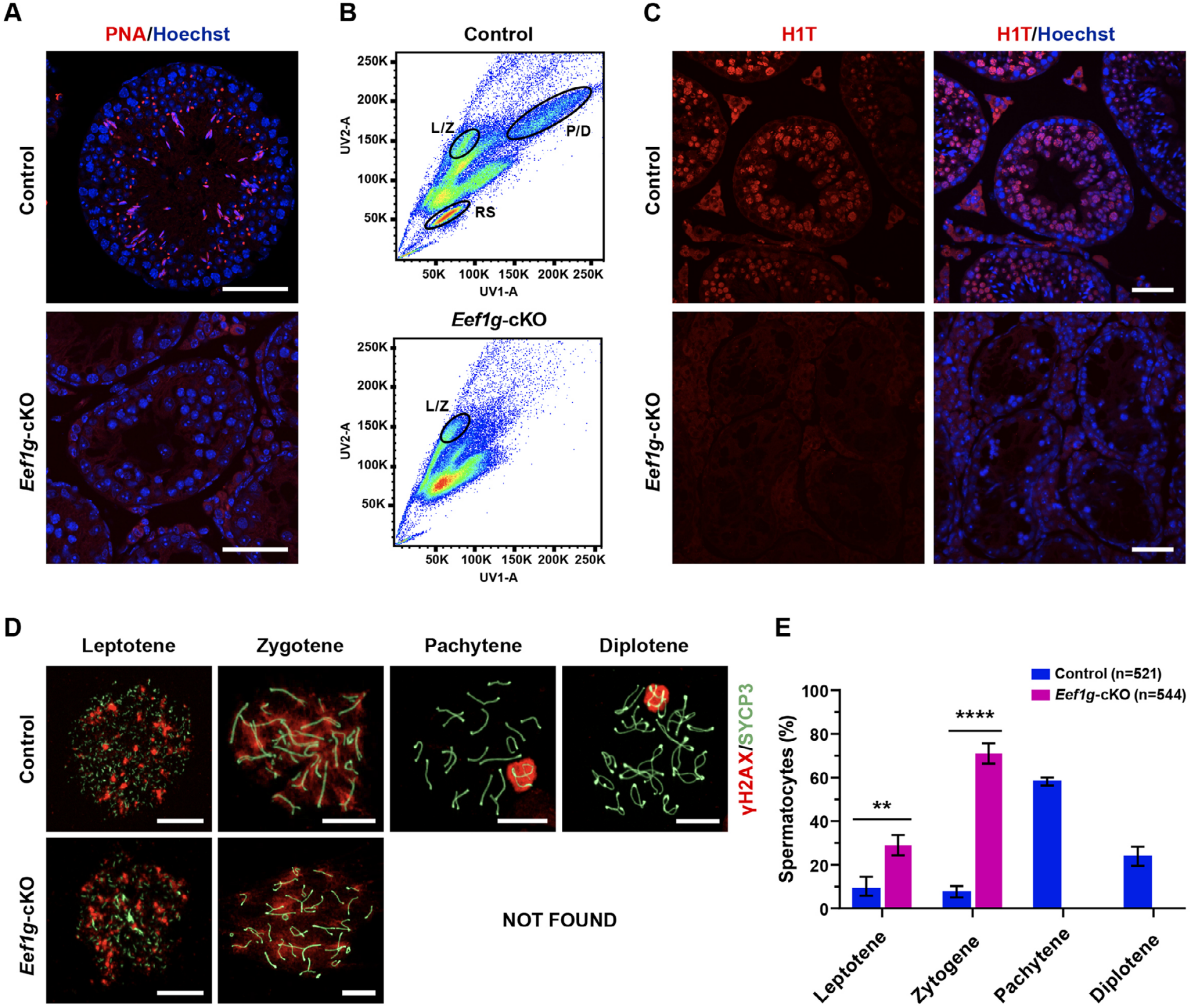
*Eef1g* deletion in germ cells causes meiotic zygotene arrest. (A) Immunostaining for PNA in testis sections from 10‐week‐old control and *Eef1g*‐cKO mice. Nuclei were counterstained with Hoechst. Scale bars, 50 µm. (B) Flow cytometry analysis of the spermatogenic cell population in 21 dpp control and *Eef1g*‐cKO testes. L/Z indicates leptotene and zygotene spermatocytes; P/D indicates the pachytene and diplotene spermatocytes; RS indicates round spermatids. (C) Immunostaining for H1T (red) in testis sections from 10‐week‐old control and *Eef1g*‐cKO mice. Nuclei were counterstained with Hoechst. Scale bars, 50 µm. (D) Representative images of spread spermatocytes from 10‐week‐old control and *Eef1g*‐cKO mice stained with antibodies against SYCP3 (green) and γH2AX (red). Scale bars, 10 µm. (E) The proportions of spermatocytes at the indicated substages. Stages were determined based on SYCP3 morphology and γH2AX distribution (see Materials and Methods). The data are from three independent experiments, and the bars represent the mean ± SEMs. The “n” indicates the number of meiotic prophase cells examined. ^**^ *p* < 0.01; ^****^ *p* < 0.0001; Student's *t*‐test.

To identify the specific substage of meiotic prophase I at which *Eef1g* deletion triggers spermatogenesis arrest, we first confirmed the absence of postmeiotic germ cells in *Eef1g*‐cKO mice by performing immunostaining with peanut agglutinin (PNA), an acrosomal marker that labels spermatids and sperms [38], on their testicular sections (Figure 3A). Flow cytometry (FACS) analysis further revealed that haploid spermatids, along with pachytene and diplotene spermatocytes, were completely absent in the spermatogenic cell population of 21 dpp *Eef1g*‐cKO testes (Figure 3B). We then co‐stained H1T (testis‐specific histone H1), a marker of spermatocytes later than mid‐pachytene [39, 40], in testicular sections from control and *Eef1g*‐cKO mice (Figure 3C), and showed that no H1T‐positive signals were detected in spermatocytes of *Eef1g*‐cKO mice, indicating the absence of spermatocytes beyond mid‐pachytene. We next performed co‐staining of γH2AX and SYCP3 on spread spermatocytes from both adult control and *Eef1g*‐cKO mice. Control samples contained spermatocytes at leptotene, zygotene, pachytene, and diplotene stages (Figure 3D,E). In contrast, pachytene spermatocytes were undetectable in *Eef1g*‐cKO testes (Figure 3D,E), indicating meiotic arrest at zygotene upon *Eef1g* deletion. This observation was further corroborated by histological analysis of the first wave of spermatogenesis. For instance, hematoxylin staining showed no obvious differences between control and *Eef1g*‐cKO testes at 10 dpp (Figure S4A). At 14 dpp, pachytene spermatocytes were clearly observed in the seminiferous tubules of control mice, whereas they were completely absent in those of *Eef1g*‐cKO mice (Figure S4B). Immunostaining of γH2AX in seminiferous tubules of 14 dpp control and *Eef1g*‐cKO testes further confirmed the absence of pachytene and diplotene spermatocytes (Figure S4C). Further co‐immunostaining for γH2AX and VASA, a marker for germ cells, in 10, 12, and 14 dpp mouse testes from control and *Eef1g*‐cKO mice showed that the number of VASA^+^ germ cells starts to decrease after 12 dpp, and γH2AX^+^ (indicative of spermatocytes) germ cells also decreased after 12 dpp. These results indicate the *Eef1g* deletion in germ cells causes meiotic arrest at the zygotene stage without impairing spermatogonia (Figure S5A–C). Taken together, these results demonstrate that eEF1G is essential for the progression of meiosis beyond the zygotene stage.

### 2.4 eEF1G Is Required for Homologous Chromosome Synapsis during Meiotic Prophase I

**FIGURE 4:**
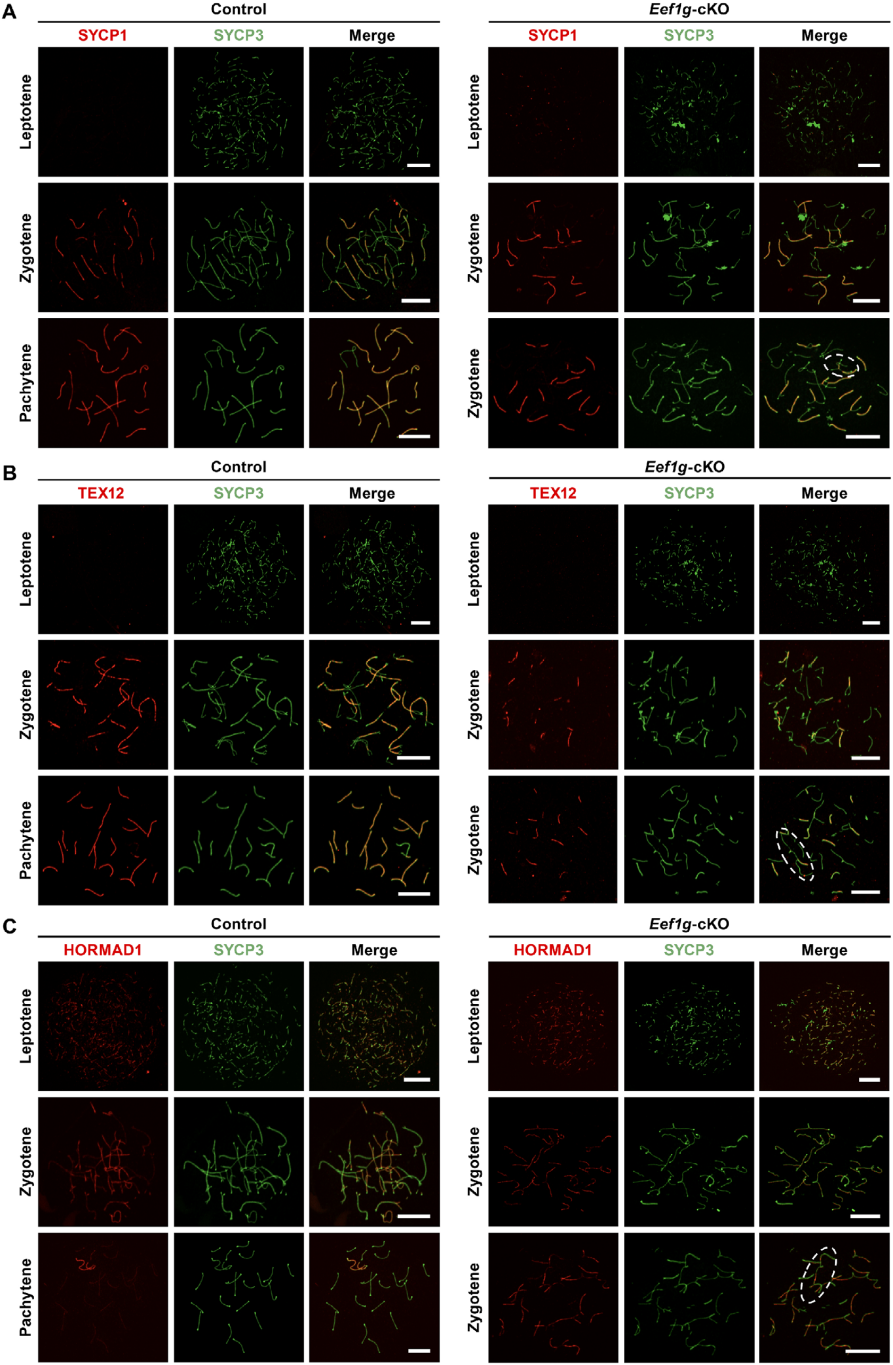
*Eef1g‐*deficient spermatocytes exhibit synaptic defects. (A) Representative spread spermatocytes from 10‐week‐old control and *Eef1g*‐cKO mice stained with antibodies against SYCP3 (green) and SYCP1 (red). Scale bars, 10 µm. The chromosome axes showed that non‐homologous synapsis was circled with white dashed lines. (B) Representative images of spermatocyte spreads from 10‐week‐old control and *Eef1g*‐cKO mice stained for SYCP3 (green) and TEX12 (red). Scale bars, 10 µm. The chromosome axes showed non‐homologous synapsis were circled with white dashed lines. (C) Representative images of spermatocyte spreads from 10‐week‐old control and *Eef1g*‐cKO mice stained for SYCP3 (green) and HORMAD1 (red). Scale bars: 10 µm. The chromosome axes showed non‐homologous synapsis were circled with white dashed lines.

To investigate the role of eEF1G in chromosomal synapsis, we performed immunostaining on spermatocyte spreads using antibodies against SYCP3 and SYCP1, a transverse filament of the synaptonemal complex (SC) [10]. As expected, in control spermatocytes, SYCP1 signals first appeared in the homologous pairing regions during the early zygotene stage, and extended along the entire length of autosomal axes and the pseudoautosomal region (PAR) of sex chromosomes by the pachytene stage (Figure 4A). By contrast, *Eef1g*‐cKO spermatocytes were arrested at the zygotene stage and displayed unsynapsed or partially synapsed chromosomal axes, as well as ectopic partner switch (an indicator of non‐homologous synapsis) [41] (Figure 4A and Figure S6A,B), indicating that homologous synapsis is defective in the absence of eEF1G. Co‐staining for SYCP3 and TEX12 (Figure 4B) or SYCE1 (Figure S6C), two central elements of the synaptonemal complex [12, 42], further showed synapsis abnormalities, consistent with the synapsis defects observed in SYCP1 staining. We also immunostained spermatocytes for HORMAD1, which is generally localized to unsynapsed chromosome axes [43]. We found that in *Eef1g*‐cKO spermatocytes, HORMAD1 localized to chromosome axes, indicating defective chromosome synapsis (Figure 4C). Together, these findings indicate that eEF1G is essential for the completion of proper chromosomal synapsis during meiosis (Figure S6D).

### 2.5 eEF1G Is Required for Meiotic Recombination through Stabilization of Recombination Intermediates

**FIGURE 5:**
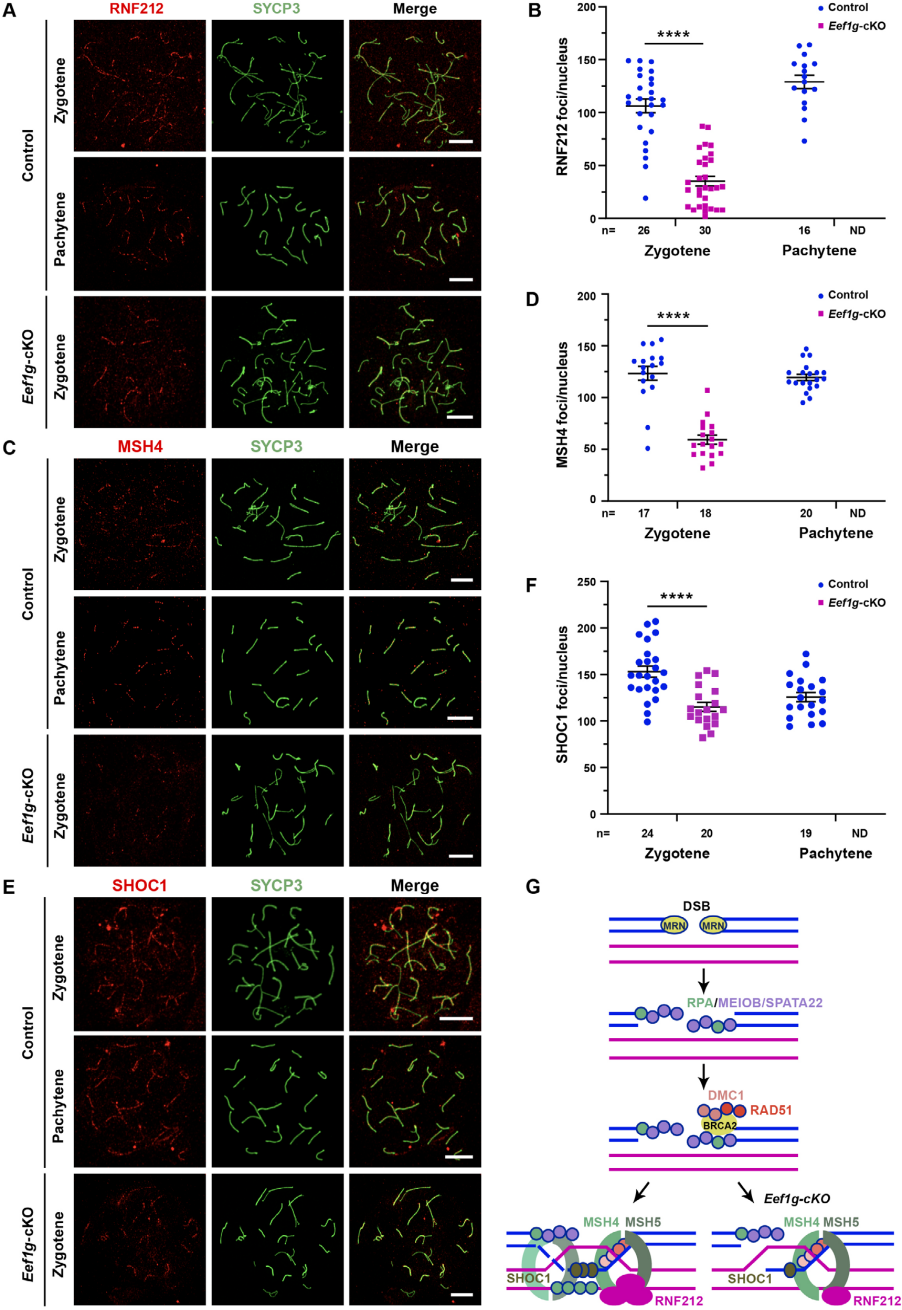
*Eef1g* ablation in germ cells decreases recombination intermediates in spermatocytes. (A) Representative images of spread spermatocytes from 10‐week‐old control and *Eef1g*‐cKO mice immunostained for SYCP3 (green) and RNF212 (red). Zygotene and pachytene spermatocytes are shown. Scale bars: 10 µm. (B) Quantification of RNF212 foci in spermatocytes at the indicated substages. n, the total number of nuclei analyzed from two animals for each genotype. The bars represent mean ± SEMs. ^****^ *p*  < 0.0001; Student's *t*‐test. (C) Representative images of spread spermatocytes from 10‐week‐old control and *Eef1g*‐cKO mice immunostained for SYCP3 (green) and MSH4 (red). Zygotene and pachytene spermatocytes are shown. Scale bars: 10 µm. (D) Quantification of MSH4 foci in spermatocytes at the indicated substages. n, the total number of nuclei analyzed from three animals for each genotype. The bars represent mean ± SEMs. ^****^ *p*  < 0.0001; Student's *t*‐test. (E) Representative images of spread spermatocytes from 10‐week‐old control and *Eef1g*‐cKO mice immunostained for SYCP3 (green) and SHOC1 (red). Zygotene and pachytene spermatocytes are shown. Scale bars: 10 µm. (F) Quantification of SHOC1 foci in spermatocytes at the indicated substages. n, the total number of nuclei analyzed from two animals for each genotype. The bars represent mean ± SEMs. ^****^ *p*  < 0.0001; Student's *t*‐test. (G) Schematic model of the role of eEF1G in meiotic recombination. In *Eef1g‐*cKO spermatocytes, the localization of RNF212 and SHOC1 onto DSBs is reduced. Consequently, the localization of the recombination intermediate (MSH4) is also reduced.

Since homologous recombination (HR) and synapsis are interdependent and tightly coupled processes during meiotic prophase I, we further investigated the role of eEF1G in homologous recombination. We first examined the late recombination nodule components MutLγ (MLH1) and ubiquitin ligase HEI10 in spread spermatocytes, both of which are essential for the formation of class I crossovers, and normally appear in mid‐to‐late pachytene spermatocytes [44, 45, 46]. Given the zygotene arrest of *Eef1g*‐cKO spermatocytes, MLH1 and HEI10 foci were absent in *Eef1g*‐cKO spermatocytes. While this absence is consistent with a failure to form class I crossovers, it also reflects the developmental arrest before the pachytene stage (Figure S7A–D). Therefore, we examined additional recombination factors that act earlier, at the zygotene stage, to pinpoint the defect. The SUMO ligase RNF212 plays a crucial role in designating crossover sites by stabilizing the ZMM protein MSH4 [46, 47, 48]. We examined the chromosomal localization of RNF212 in spread spermatocytes (Figure 5A,B). As previously reported [46, 48], RNF212 foci first appeared on synapsed chromosome axes during the zygotene stage in control spermatocytes. By contrast, *Eef1g*‐cKO mice displayed fewer RNF212 foci compared to control mice (35.13 ± 4.55 foci per nucleus in *Eef1g*‐cKO vs. 106.27 ± 6.53 foci per nucleus in control, mean ± SEMs), suggesting that RNF212 recruitment is impaired in *Eef1g*‐cKO mutants. We next examined MSH4 dynamics using immunostaining of spermatocyte spreads (Figure 5C,D). In control mice, MSH4 foci were detected in the synapsed regions of homologous chromosomes during zygotene (n = 123.35 ± 6.67, mean ± SEM) and declined (n = 119.35 ± 3.00, mean ± SEM) by the pachytene stage, consistent with previous reports [49]. However, fewer MSH4 foci were observed in zygotene spermatocytes from *Eef1g*‐cKO males (n = 59.22 ± 4.32, mean ± SEM). Another ZMM family protein, SHOC1 [50], exhibited similar dynamic patterns to MSH4 during prophase I in *Eef1g*‐cKO spermatocytes (115.16 ± 4.92 foci per nucleus in *Eef1g*‐cKO zygotene vs. 153.08 ± 5.97 foci per nucleus in control zygotene, mean ± SEMs) (Figure 5E,F). These observations indicate that eEF1G is required for the accumulation of MSH4 and SHOC1 during meiotic recombination.

Given that eEF1G is required for the accumulation of RNF212, MSH4, and SHOC1, we next asked whether this defect arises from an earlier failure in early recombination events. DMC1 and RAD51 form nucleoprotein filaments that mediate homology search and strand invasion during meiotic recombination [51, 52]. We therefore examined the localization of the meiosis‐specific recombinase DMC1 and RAD51 (Figure S8A–D). In zygotene spermatocytes, the number of DMC1 foci was comparable between control and *Eef1g*‐cKO mice (147.13 ± 8.64 per nucleus in *Eef1g*‐cKO vs. 164.17 ± 7.22 foci per nucleus in control, mean ± SEMs), indicating that DSB formation and early recombination processing are largely unaffected in *Eef1g*‐cKO spermatocytes. Likewise, the number of RAD51 foci in zygotene spermatocytes did not differ significantly between *Eef1g*‐cKO and control mice (131.03 ± 6.94 per nucleus in *Eef1g*‐cKO vs. 113.00 ± 5.76 foci per nucleus in control, mean ± SEMs), suggesting that homology search and strand invasion are not grossly impaired in the absence of eEF1G. Together, these results place the defect downstream of DSB formation and early strand invasion, but upstream of crossover designation. Therefore, the impaired recruitment of downstream crossover factors (RNF212, MSH4, and SHOC1) and the absence of class I crossovers are not due to a failure in DSB formation or initial strand exchange, but rather reflect a failure in the stabilization of recombination intermediates. Collectively, these results identify eEF1G as a factor required for recombination intermediate stabilization during meiotic recombination (Figure 5G).

### 2.6 Meiosis‐Related Proteins Are Selectively Downregulated in *Eef1G*‐Deficient Spermatocytes

**FIGURE 6:**
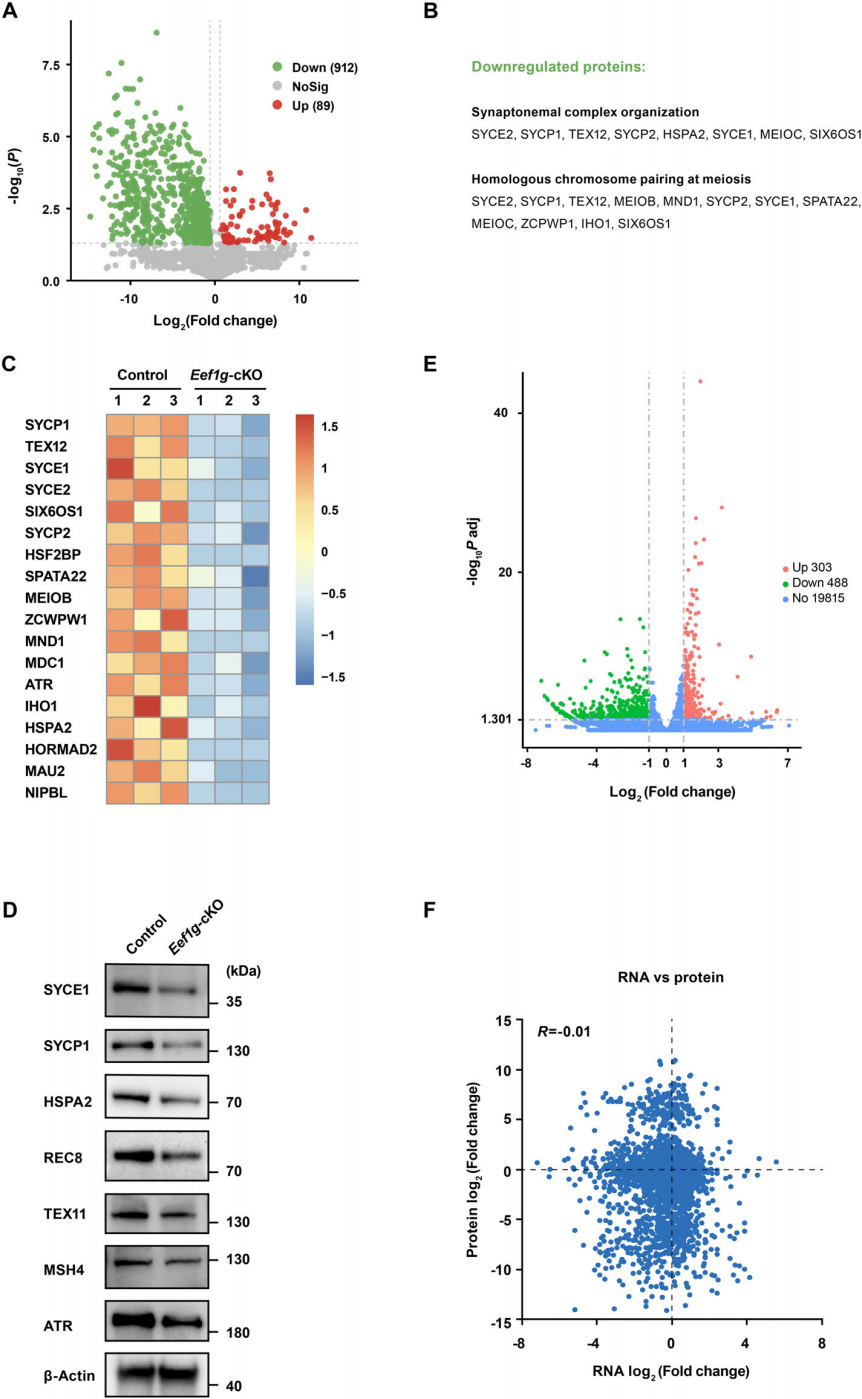
Meiotic proteins are decreased in leptotene and zygotene spermatocytes upon *Eef1g* deletion. (A) Volcano plot showing differentially expressed proteins identified via mass spectrometry proteomic analyses (*p* < 0.05, |log_2_ (fold change)| > 0.585, n = 3). (B) Gene ontology enrichment analysis for the biological process of downregulated proteins in *Eef1g*‐deficient leptotene and zygotene spermatocytes. Representative pathways and genes in the downregulated groups are shown. (C) Heatmap depicting downregulated proteins related to synaptonemal complex formation and meiotic homologous recombination that were significantly downregulated in *Eef1g*‐deficient leptotene and zygotene spermatocytes. The color scale represents row‐wise z‐score–normalized protein abundance. (D) Representative Western blot showing the protein levels of factors known to be essential for meiotic recombination and synaptonemal complex formation in the testis of 12 dpp *Eef1g*‐cKO and control mice. (E) Volcano plot showing differentially expressed RNAs identified via transcriptional analysis (*p* < 0.05, |log_2_ (fold change)| > 1, n = 3).  (F) Scatter plot showing the relationship between RNA and protein expression changes in *Eef1g*‐cKO spermatocytes relative to control spermatocytes. For each gene, the *x*‐axis represents the log_2_ (fold change) in RNA levels, and the *y*‐axis represents the log_2_ (fold change) in protein levels. The Pearson correlation coefficient (R) is −0.01, indicating no linear correlation between RNA and protein fold changes under this genetic perturbation.

To investigate the molecular consequences of eEF1G deletion in meiosis, we purified leptotene/zygotene spermatocytes from the *Eef1g*‐cKO and littermate control testes at 12 dpp using FACS [53] (Figure S9A,B). To assess the purity of sorted spermatocytes, we performed immunostaining with SYCP3 and γH2AX (Figure S9C). Quantification showed that the proportion of leptotene/zygotene spermatocytes (SYCP3^+^/γH2AX^+^) was 82.67% ± 2.36 (mean ± SEM) in control and 84.66% ± 2.38 (mean ± SEM) in *Eef1g*‐cKO samples (Figure S9D), indicating comparable enrichment efficiency between genotypes. These sorted cells were then subjected to mass spectrometry (MS) analysis. Proteomic analyses identified 1001 differentially expressed proteins (*p* < 0.05, |log_2_ (fold change)| > 0.585, n = 3), including 89 upregulated and 912 downregulated proteins (Figure 6A and Table S1). Gene Ontology (GO) analysis revealed that the downregulated proteins were enriched for meiotic nuclear division (Figure 6B). Notably, five of these proteins (Figure 6C), SYCP1 [10], SYCE1 [12], SYCE2 [54], TEX12 [42, 55], and SIX6OS1 [6], are directly involved in the formation of the synaptonemal complex; seven proteins (Figure 6A), HSF2BP [41, 56, 57], SPATA22 (La [58, 59]), MEIOB [60, 61, 62], ZCWPW1 [63, 64], MND1 [65, 66], MDC1 [67], and ATR [68, 69, 70] were directly linked to the repair and recombination of meiotic chromosomes, three proteins, IHO1, HORMAD2, and HSPA2 were linked to chromosome axes, and three proteins (REC8, MAU2 and NIPBL) were cohesion proteins. The complete ablation of most of these proteins has been shown to cause impaired development of meiosis in mouse models. Western blot further confirmed markedly reduced levels of SYCE1, SYCP1, HSPA2, REC8, TEX11, MSH4, and ATR in 12 dpp *Eef1g*‐cKO testes compared to controls (Figure 6D).

We further performed RNA‐seq analyses of the FACS‐sorted leptotene/zygotene spermatocytes at 12 dpp (Figure 6E and Table S2), which showed that 303 genes were upregulated and 488 genes were downregulated (*p* < 0.05, |log_2_ (fold change)| > 1, n = 3) (Figure 6E and Table S2). Strikingly, GO analyses revealed that the upregulated genes were associated with the meiotic nuclear division and cell cycle processes (Figure S10A,B), the opposite of the proteomic trend (Figure 6B). Integrative analysis of the two datasets revealed a near‐zero correlation between protein and transcript changes (Pearson correlation coefficient *R* = −0.01) (Figure 6F), demonstrating that the proteomic defects are largely post‐transcriptional.

Taken together, these findings suggest that loss of eEF1G compromises the ability of leptotene/zygotene spermatocytes to produce sufficient amounts of short‐lived meiotic proteins, despite stable mRNA levels. As a result, key synapsis and recombination factors fail to accumulate to the levels required for normal meiotic progression.

### 2.7 eEF1G Associates with Ribosomal Proteins and Supports Translation of Meiosis‐Related mRNAs in Spermatocytes

**FIGURE 7:**
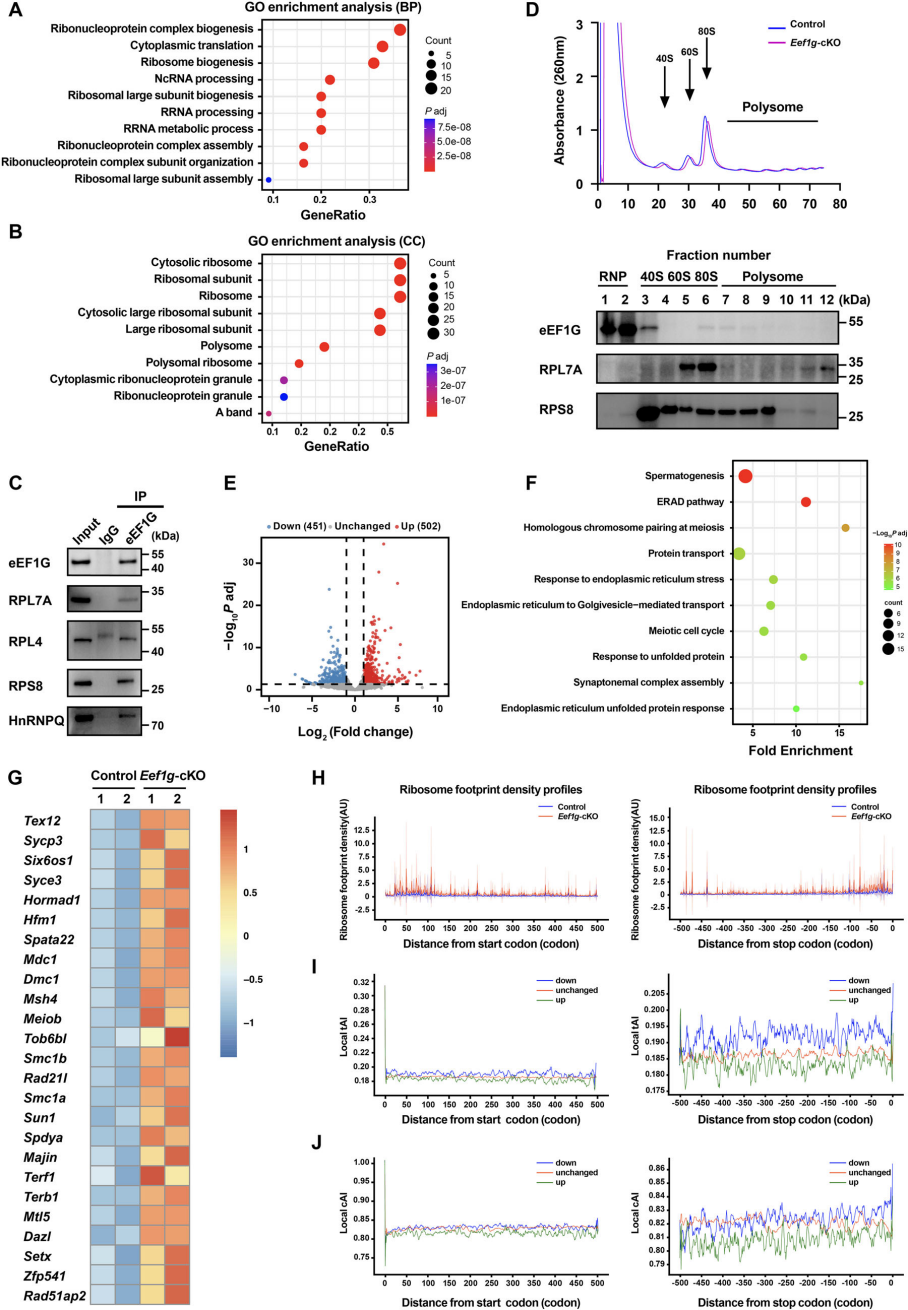
eEF1G interacts with ribosomal proteins and promotes translation in spermatocytes. (A) Gene Ontology enrichment analysis for the biological process of eEF1G‐interacting proteins in leptotene and zygotene spermatocytes from 12 dpp mouse testes. (B) Gene Ontology enrichment analysis for the cellular component of eEF1G‐interacting proteins in leptotene and zygotene spermatocytes from 12 dpp mouse testes. (C) Western blot validation of the interactions between eEF1G and the indicated ribosome‐associated proteins in leptotene and zygotene spermatocytes from 12 dpp mouse testes. (D) Polysomal profiling and co‐sedimentation profiles of testicular lysates following *Eef1g* knockout in germ cells. (E) Volcano plot showing differentially enriched ribosome‐protected mRNA fragments (RPFs) identified by Ribo‐seq analyses (*p* < 0.05, |log_2_ (fold change)| > 1, n = 2). (F) Gene ontology enrichment analysis for Biological Process of upregulated RPFs in *Eef1g*‐deficient leptotene and zygotene spermatocytes. (G) Heatmap depicting upregulated RPFs from Ribo‐seq related to synaptonemal complex formation, meiotic homologous recombination, meiotic telomere complex formation, cohesion complex, and meiosis in *Eef1g*‐deficient leptotene and zygotene spermatocytes. (H) Global read distribution along transcripts for transcripts with upregulated RPFs. (I) Distribution of the local tRNA adaptation index (tAI) along transcripts for different gene sets. (J) Distribution of the local codon adaptation index (cAI) along transcripts for different gene sets.

To investigate how loss of eEF1G impairs protein production during early meiosis, we performed co‐immunoprecipitation (co‐IP) to characterize the interactome of eEF1G in leptotene/zygotene spermatocytes enriched from 12 dpp wild‐type mice using the anti‐eEF1G antibody, followed by mass spectrometry analysis. MS results identified 58 eEF1G‐interacting proteins, among which 31 were ribosomal proteins (including 27 large ribosomal subunits and 4 small ribosomal subunits), and 27 were RNA‐binding proteins (Table S3). GO analysis revealed that these eEF1G‐associated proteins are enriched in processes related to ribonucleoprotein complex biogenesis, cytoplasmic translation, ncRNA processing, and rRNA processing (Figure 7A,B), consistent with eEF1G's role as part of the translation machinery. We confirmed the interaction between eEF1G and ribosome/translation‐related proteins, including RPL4, RPL7A, and RPS8, using co‐IP in 12 dpp leptotene/zygotene spermatocytes (Figure 7C), indicating that eEF1G participates in the translation machinery in spermatocytes.

We next asked whether eEF1G loss destabilizes its known interaction partners or other eEF1 complex subunits. Immunofluorescence on paraffin‐embedded testis sections showed that eEF1A1, eEF1B2, RPL7A, and RPS8 all remained cytoplasmic in *Eef1g*‐cKO spermatocytes; however, eEF1B2 signal intensity was visibly reduced, whereas eEF1A1, RPL7A, and RPS8 were unaffected (Figure S11A–D). Western blot of 12 dpp testis lysates corroborated this selectivity: eEF1B2 and eEF1D protein levels were reduced in *Eef1g*‐cKO mice, while eEF1A1, RPL7A, and RPS8 remained unchanged (Figure S11E). These findings indicate that eEF1G acts as a stabilizing scaffold for the eEF1B subcomplex (eEF1B2/eEF1D/eEF1G) [24], without broadly disrupting eEF1A1 or ribosome‐associated proteins.

To directly assess the impact of eEF1G on translation, we performed ribosome profiling (Ribo‐seq) in leptotene/zygotene spermatocytes, also at 12 dpp, by optimizing a protocol tailored for low‐input samples (Figure 7D,E) (Methods). The mapped reads exhibited strong 3‐nt periodicity (Figure S12A) and were strongly enriched in the first reading frame (Figure S12B). Two replicates showed strong correlations (Figure S12C). 502 genes with increased and 451 genes with decreased RPF abundance (*p* < 0.05, |log_2_ (fold change)| > 1, n = 2) were identified in *Eef1g*‐cKO leptotene/zygotene spermatocytes (Figure 7E and Table S4). The down‐regulated genes were mainly involved in positive/negative regulation of transcription by RNA polymerase II, and cell migration, etc (Figure S13). Notably, the upregulated genes were mainly associated with meiotic processes, such as synaptonemal complex formation (e.g., *Tex12*, *Sycp3*, *Six6os1*, *Syce3*, and *Hormad1*), meiotic recombination (e.g., *Hfm1*, *Spata22*, *Mdc1*, *Dmc1*, *Msh4*, *Meiob*, and *Top6bl*), cohesin complex components (*Smc1b* [71], *Rad21l* [72, 73], and *Smc1a*), meiotic telomere formation (e.g., *Sun1* [74], *Spdya* [75], *Majin* [76], *Terf1* [77]), and *Terb1* [78]), and other spermatogenesis‐related genes (e.g., *Mtl5* [79], *Dazl*, *Setx*, *Zfp541* [40],  and *Rad51ap2*) (Figure 7G). The increase in ribosome‐protected fragments (RPFs) on these transcripts is consistent with altered ribosome progression and increased ribosome occupancy on specific regions of these mRNAs in *Eef1g*‐cKO spermatocytes.

To determine whether the elevated ribosome occupancy on meiotic transcripts reflects genuine translational upregulation, we calculated translational efficiency (TE) as the ratio of RPF abundance to mRNA abundance (RPF/FPKM) using matched Riboseq and RNAseq data from the same FACS‐purified leptotene/zygotene spermatocytes (Figure S14A and Table S5). In total, 144 genes showed significantly increased TE (ΔTE Log_2_ FC > 0, adjusted *p* < 0.05), and 168 genes showed significantly decreased TE (ΔTE Log_2_ FC < 0, adjusted *p* < 0.05) (Figure S14A). GO analysis of the TE‐upregulated genes revealed terms related to protein localization to the cell periphery, the synaptic vesicle cycle, and regulation of glycoprotein biosynthesis, but no terms associated with meiosis (Figure S14B). Notably, no significant GO terms were enriched among the TE‐downregulated genes. Manual inspection further confirmed that known meiotic genes, including those encoding SC and recombination proteins, were not overrepresented in either group. Thus, although RPF density is elevated on meiotic transcripts in *Eef1g*‐cKO spermatocytes, their TE is not selectively changed. This suggests that the increased RPF density likely reflects altered ribosome dynamics (e.g., elongation pausing) rather than a genuine increase in translation output, a conclusion consistent with the reduced protein levels of these meiotic proteins (Figure 6).

We next plotted the average ribosome occupancy of all genes [80]. While the coding and 3' UTR ribosome densities were similar between control and *Eef1g*‐cKO spermatocytes, the 5' UTR density was slightly lower in the knockout (Figure S15A). Ribosome polarity scores were comparable between *Eef1g*‐deficient and control leptotene and zygotene spermatocytes [81] (Figure S15B), indicating that we do not detect a strong directional shift in ribosomes toward the 5′ or 3′ ends of coding sequences upon *Eef1g* deletion. These global measures, however, do not exclude more subtle or stochastic changes in ribosome pausing. To further examine ribosome distribution on the most affected transcripts, we analyzed relative ribosome occupancy for genes with increased RPF abundance and observed overall elevated footprint density in *Eef1g*‐cKO spermatocytes (Figure 7H), confirming altered ribosome progression on these specific mRNAs.

We then calculated the codon adaptation index (cAI) (Figure 7I) and tRNA adaptation index (tAI) [81] (Figure 7J), and found that transcripts with increased ribosome footprint abundance (genes with upregulated RPFs) tend to have significantly smaller local tAI and local cAI values compared to other transcripts. Analysis of global tAI (Figure S15C) or cAI (Figure S15D) yields similar results. These observations raise the possibility that transcripts enriched in relatively suboptimal codons may be more susceptible to altered ribosome progression upon eEF1G loss. Amino acid‐ and codon‐level analysis of ribosome density changes (Figure S16A‐D) shows that RPF‐upregulated transcripts had elevated ribosome density across virtually all 20 standard amino acids, consistent with broadly increased occupancy. In RPF‐downregulated transcripts, ribosome density was moderately reduced for most amino acids, though tyrosine showed a marked increase whose significance is unclear.

Collectively, these results establish that eEF1G is required for efficient translation of meiosis‐related mRNAs encoding SC and recombination proteins, and that its absence causes decreased protein levels needed for meiotic progression.

## 3 Discussion

This study identifies eEF1G as a critical factor required for meiosis progression in transcriptionally quiescent spermatocytes. Germ cell‐specific deletion of *Eef1g* causes meiotic arrest at the zygotene stage, with persistent DSBs, impaired homologous recombination, and defective synapsis, phenocopying mutants lacking synaptonemal complex components or recombination machinery. Integrated Ribo‐seq and proteomics demonstrate that eEF1G loss reduces the translational capacity of leptotene and zygotene spermatocytes, a stage in which transcription is globally suppressed, and rapid synthesis of short‐lived meiotic proteins is essential. Under these conditions, it is plausible that reduced translational efficiency disproportionately affects rapidly turning‑over proteins required for synapsis and recombination, providing a straightforward explanation for the observed meiotic failure. These findings underscore the vulnerability of early meiosis to perturbations in the core translation machinery and establish that a generalized reduction in translational output is sufficient to cause meiotic failure and male infertility.

Since homologous recombination and synapsis are interdependent during meiotic prophase I, the observed recombination failure could theoretically be secondary to defective synapsis. However, several lines of evidence suggest the primary defect lies in recombination intermediate stabilization. First, normal DMC1 and RAD51 foci in *Eef1g*‐cKO zygotene spermatocytes (Figure S8) indicate intact DSB formation, end processing, and strand invasion. Second, reduced MSH4 and SHOC1 (ZMM proteins acting after strand invasion but before crossover designation) point to a specific failure in stabilizing early recombination intermediates. Third, despite reduced SYCP1 and SYCE1 levels, partial SYCP1 localization along chromosome axes remains detectable (Figure 4A), suggesting that synapsis defects are not absolute and are likely aggravated by unstable recombination intermediates. Nonetheless, given known feedback loops, we cannot fully exclude that some recombination defects arise secondarily from impaired synapsis. Future experiments with finer temporal control (e.g., induction after DSB formation) are needed to dissect the hierarchy. For now, we conclude that eEF1G is required to stabilize recombination intermediates, a function essential for both proper synapsis and crossover formation (Figure 5G).

eEF1G shows germline‐enriched expression, consistent with the heightened translational demand of spermatocytes during the transcriptionally quiescent leptotene/zygotene stage [14]. Unlike somatic cells that continuously replenish mRNA, these spermatocytes must exploit pre‐existing transcripts to synthesize proteins required for DNA repair, homologous recombination, and synapsis [29, 82]. The widespread decrease in meiotic protein abundance in *Eef1g*‐deficient spermatocytes, therefore, likely reflects reduced global translation rather than transcript‐specific regulation.

Our findings also suggest that different stages of germ cell development may exhibit differential sensitivity to reduced translation. For example, germline deletion of eIF5 causes apoptotic loss of progenitor spermatogonia via ROS/DNA damage [83]. In contrast, the heterozygous eEF1A1b mutation in tilapia allows spermatogonia and meiotic cells to develop normally but impairs spermiogenesis [33]. Similarly, removal of eIF5A permits spermatogonia to differentiate and meiosis to finish, yet stalls development at the round spermatid stage [84]. In our model, *Eef1g* deficiency preserves spermatogonia but impairs progression through early meiosis. This suggests that spermatogonia, spermatocytes, or spermatids differ in their sensitivity to reductions in translational output. Such stage‐specific sensitivities imply that germ cells depend differently on individual components of the translational apparatus, although the possibility of stage‐specific non‐canonical functions for certain translation factors cannot be excluded. Distinguishing between these possibilities will require further mechanistic investigation.

From an evolutionary perspective, the pronounced requirement for eEF1G during mammalian meiosis may reflect the particular physiological and kinetic constraints of vertebrate spermatogenesis. In contrast to oogenesis, which involves prolonged arrest and relies on long‐lived proteins [85], spermatogenesis is a highly dynamic and continuous process that requires rapid protein turnover to ensure the timely coordination of essential meiotic events, including double‐strand break (DSB) repair, synapsis, and crossover formation [86]. These demands may render early meiosis highly dependent on efficient elongation, although whether this represents evolutionary specialization or simply differential sensitivity to reduced translational capacity remains unclear.

**FIGURE 8:**
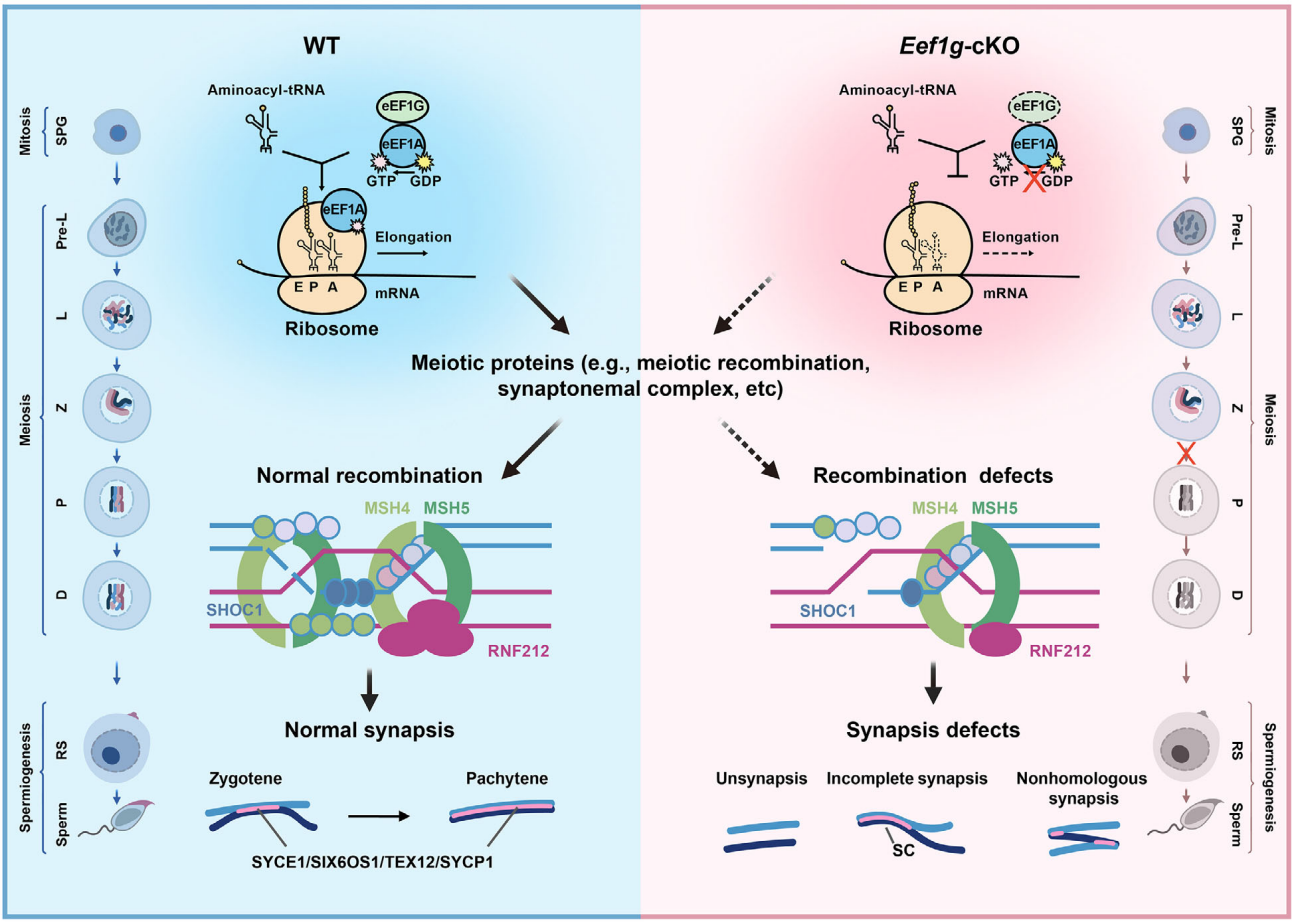
Proposed model of eEF1G function in regulating translation during meiosis. eEF1G, as a scaffolding subunit of the eEF1B complex, promotes efficient translation elongation of meiosis‐related mRNAs, ensuring sufficient proteins for recombination and synaptonemal complex assembly. Loss of eEF1G reduces these proteins, causing defective homologous synapsis and recombination, zygotene arrest, and ultimately male infertility.

eEF1G is a scaffolding subunit of the eEF1B complex, which functions as a guanine nucleotide exchange factor (GEF) for eEF1A [24]. Consistent with its proposed scaffolding role, we found that eEF1G deletion in mouse spermatocytes selectively reduces the protein levels of its binding partners eEF1B2 and eEF1D, without affecting the localization or levels of eEF1A1 or the ribosomal protein RPL7A and RPS8 (Figure S11A–D). This observation aligns with a previous study in human A549 cells, in which CRISPR/Cas9‐mediated disruption of eEF1G similarly causes decreased expression of eEF1B2 and eEF1D [24], supporting a conserved scaffolding function in protecting the eEF1B subcomplex from degradation. Our findings highlight its importance for maintaining adequate translational output during meiosis, although we do not establish a distinct mRNA‐specific regulatory function. Many mRNAs encoding recombination, synapsis, telomere‐related, and cohesion proteins show altered ribosome occupancy and/or reduced protein output upon *Eef1g* deletion. Reduced synthesis of these factors provides a coherent explanation for the synaptic and recombination defects, resembling phenotypes of *Tex12*, *Syce1*, *Six6os1*, *Meiob*, *Hsf2bp*, and *Msh4* mutants. Thus, eEF1G is an essential component of the translational machinery that supports early meiotic progression (Figure 8).

Ribosome profiling reveals increased ribosome occupancy on transcripts enriched for relatively suboptimal codons, despite reduced protein abundance. One interpretation is that ribosome progression is slowed, leading to higher footprint density without corresponding increases in protein output, although the underlying mechanism remains unresolved. These observations align with prior studies showing that increased ribosome density does not necessarily translate into higher protein synthesis when elongation is impaired [80, 87]. However, it remains possible that other mRNA features, such as secondary structure or RNA‐binding protein interactions, also contribute to the observed differences, which warrants further investigation. Our translational efficiency (TE) analysis further showed that meiotic genes are not selectively enriched among TE‐altered genes (Figure S14A,B), indicating that the increased ribosome occupancy on these transcripts reflects altered elongation dynamics rather than a genuine increase in translation output. Together, these findings emphasize the importance of integrating proteomics with ribosome profiling to accurately infer translational dynamics.

The translational defects uncovered here may have direct clinical relevance to idiopathic non‐obstructive azoospermia (NOA), a condition in which many patients present with meiotic arrest of unknown etiology [88, 89]. Conventional diagnostic pipelines centered on DNA sequencing and transcriptomics are blind to post‐transcriptional failures in which mRNA levels are normal, but protein levels are insufficient. Incorporating functional measures of translational activity into the diagnostic workup of azoospermic patients could reveal a class of infertility driven by elongation insufficiency that currently goes undetected.

In summary, our findings highlight eEF1G as a core translation factor whose activity is crucial for meiotic progression in transcriptionally quiescent spermatocytes. By sustaining sufficient protein synthesis during the leptotene/zygotene stages, eEF1G enables the production of factors required for synapsis and recombination, thereby ensuring meiotic fidelity and male fertility. More broadly, this work supports the view that defects in general translation factors can impair spermatogenesis, particularly in cells operating under high translational demand and limited transcription.

## 4 Limitations

Whether the synapsis defects, specifically, mislocalization of SYCP1, SYCE1, and TEX12, are a direct consequence of translational insufficiency or arise secondarily from defective recombination intermediates remains unresolved. Although eEF1G physically associates with ribosomal proteins and RNA‐binding proteins, precisely how it influences elongation dynamics or why particular mRNA classes are preferentially affected remains unclear. Finally, the relative resilience of spermatogonia to *Eef1g* loss, despite broad germline expression, hints at stage‐specific compensatory mechanisms that remain to be identified.

## 5 Experimental Model and Study Participant Details

### 5.1 Animals

*Eef1g* ^flox/+^ mice on a C57BL/6J background were generated by Cyagen Biosciences using a CRISPR‐Cas9‐mediated genome editing system, and *Stra8*‐GFPCre mice were a generous gift from Professor Minghan Tong of the Chinese Academy of Sciences. The *Stra8*‐GFPCre mice were used to generate germ cell‐specific *Eef1g*‐deficient mice (*Eef1g‐*cKO mice) with a 3069 base pair deletion (6350‐7334), which specifically targeted the exons 2 to 7 of the *Eef1g* gene (NM_001166589). The founders were tested with genotyping performed by polymerase chain reaction (PCR) (Primers used in this study were listed in Table S6), followed by DNA sequence analysis. All mice were housed in the Model Animal Research Center of Shandong University and exposed to a 12‐h light‐dark cycle under specific pathogen‐free (SPF) conditions, with controlled temperature (22°C ± 1°C) and humidity (40%–70%). All experimental protocols were approved by the Institutional Animal Care and Use Committee of Shandong University.

### 5.2 Histological Analysis and Immunostaining

Freshly isolated mouse testes and epididymides were fixed in Bouin's solution (Sigma, HT10132) immediately after euthanasia. Post‐fixed tissues were then paraffin‐embedded and cut into 5 µm slices using a HistoCore Autocut microtome (Leica). Histological analysis of testis and epididymis sections, along with immunofluorescence staining of testis sections, was performed as previously described [40]. For histological examination, sections were stained with hematoxylin and observed under light microscopy. For immunostaining, paraffin sections were dewaxed, rehydrated, and subjected to antigen retrieval by heating in citrate buffer (Proteintech, PR30001). Sections were then blocked with antibody dilution buffer (10% normal donkey serum, 3% bovine serum albumin [BSA], 0.05% Triton X‐100 in PBS) and incubated with primary antibodies at 4°C overnight. Afterward, primary antibodies were washed three times with 1 × PBST (PBS containing 0.1% Triton X‐100), followed by incubation with appropriate secondary antibodies at 37°C for 1 h and subsequent washing with 1 × PBST. All antibodies utilized in this study are listed in Table S7. Immunofluorescence images were captured immediately utilizing either an LSM 780/710 microscope (Zeiss) or a SP8 microscope (Leica).

### 5.3 Spermatocyte Spreading and Immunofluorescence

Spermatocyte spreading and immunofluorescence staining were performed as we described previously [40]. For spermatocyte spreading, testicular tubules were gently incubated in a hypotonic extraction buffer [30mMTris (pH 8.2), 50mMsucrose, 17 mM trisodium citrate dihydrate, 5 mM EDTA, 2.5 mM dithiothreitol (DTT), and 1 mM PMSF] for 20–25 min. The resulting cell suspension was spread onto glass slides containing 1% PFA and 0.1% Triton X‐100. Slides were dried in a humidified chamber and washed with 0.4% Photo‐Flo (Kodak). For immunofluorescence staining, slides were blocked for 30 min with 1 × PBST containing 3% nonfat milk. Slides were then incubated with primary antibodies overnight at room temperature or 37°C in a humidified chamber. After three washes with 1 × PBST, secondary antibodies were applied for 1 h at room temperature or 37°C in a humidified chamber. After secondary antibody incubation, the slides were washed in 1 × PBST three times and then mounted with VECTASHIELD mounting medium (H‐1000, Vector Laboratories) containing Hoechst 33 342 (Beyotime, C1022). Antibodies are listed in Table S7. Meiotic prophase I substages were assigned by the behavior of chromosome axes stained with SYCP3 antibodies and the distribution of γH2AX. The leptotene stage was defined as short SYCP3 stretches with widespread γH2AX signal throughout the nucleus. The zygotene stage was defined by partial synapsis of chromosome axes with persistent γH2AX. The pachytene stage was defined by complete synapsis of all autosomes with γH2AX restricted to the sex body. The diplotene stage was defined by desynapsed autosomes with γH2AX restricted to the sex body.

### 5.4 TUNEL Assay

The TUNEL assay was performed as described [40]. Briefly, testis sections were deparaffinized in xylene, rehydrated through a graded ethanol series (100%, 95%, 90%, 80%, 70%, 50% ethanol), and permeabilized with proteinase K (20 µg/mL) in 10 mM Tris‐HCl (pH 7.5) for 15 min at room temperature. After two washes with 1 × PBS, sections were blocked with 5% BSA for 30 min. The TUNEL reagent mix (Servicebio, G1504‐100T) was applied to each slide, followed by incubation at 37°C for 1 h according to the manufacturer's protocol. Sections were then washed with PBS three times and mounted in VECTASHIELD mounting medium (Vector Laboratories, H1000) containing Hoechst 33 342 (Beyotime, C1022).

### 5.5 Western Blot

Western blot was performed as previously described [40]. Briefly, testes from mice were homogenized in lysis buffer (50 mM Tris‐HCl, pH 7.5; 150 mM NaCl; 2.5 mM EDTA; and 0.5% Triton X‐100) supplemented with protease inhibitors and phenylmethylsulfonyl fluoride (PMSF) using a glass homogenizer. After denaturing by heating at 100°C in a metal bath for 10 min, the cell lysates were separated by SDS‐PAGE and transferred to a 0.45 µm Nitrocellulose Blotting membrane (GE Healthcare Life Sciences, 10 600 002) using a vertical electrophoresis and blotting apparatus (Tanon). Membranes were blocked in PBST (1 × PBS containing 0.1% Triton X‐100) with 5% nonfat milk for 30 min and incubated with primary antibodies diluted in PBST buffer containing 5% nonfat milk overnight at 4°C. After incubation with horseradish peroxidase (HRP)‐conjugated secondary antibodies for 2 h, blots were developed with chemiluminescence (GE Healthcare, ImageQuant LAS 4000). Antibodies used in Western blot are listed in Table S7.

### 5.6 Immunoprecipitation

Protein was extracted from freshly dissociated tissues using immunoprecipitation (IP) lysis buffer (Thermo Scientific, 87 788) with protease inhibitor cocktail (Beyotime, P1005) on ice for 30 min, and then centrifuged at 12000 × g for 10 min at 4°C. Total protein was preincubated according to experimental needs. Thereafter, the lysates were incubated with the relevant antibodies and precleaned magnetic beads (MecChemexpress, HY‐K0202) at 4°C. The beads were then washed three times with lysis buffer, and bound proteins were eluted from the beads in 1 × SDS sample buffer by boiling at 95°C for 10 min for Western blot analyses.

### 5.7 Fluorescence‐Activated Cell Sorting (FACS) of Spermatocytes

Germ cell populations were isolated using flow cytometry as previously described [90]. Briefly, testicular tissues were dissociated enzymatically using enzyme mixtures comprising 1 mg/mL collagenase IV (Gibco, 17 104 019), 1.5 mg/mL hyaluronidase (Sigma, H2126), 1 mg/mL trypsin (Sigma, T1426), and 1 mg/mL DNase I (Sigma, DN25), dissolved in DMEM medium at 35°C with gentle agitation for 20 min. Subsequently, the cell suspension was filtered through a 40 µm cell strainer, followed by centrifugation at 300 × g for 5 min at 4°C. The supernatant was removed, and cells in the pellet were resuspended carefully in cold DPBS (Servicebio, G4200) containing 0.05% FBS. After staining with Hoechst 33342 at 35°C for 30 min, cells were sorted by flow cytometer (BD Biosciences, FACS Aria II, USA) according to different fluorescent channels. The leptotene and zygotene spermatocytes from control and *Eef1g*‐cKO mice were then enriched and separated for RNA‐seq, Ribo‐seq, or proteomics analysis.

### 5.8 Proteomics

Leptotene and zygotene spermatocytes were isolated from 12 dpp C57BL/6 male mice by FACS and subjected to proteomic analysis. Proteins were extracted and digested with trypsin and desalted, and then analyzed using high‐precision mass spectrometry. We utilized database retrieval software, including Proteome Discoverer, Mascot, Spectronaut, and MaxQuant, for data analysis. The National Center for Protein Sciences (Beijing, China) provided the proteomics services.

### 5.9 Polysome Profiling

Polysome profiling of testicular extract was performed as described previously [91, 92]. Briefly, testes from 12 dpp control and *Eef1g*‐cKO mice were homogenized in lysis buffer (50 mM Tris‐HCl, pH 7.0, 100 mM NaCl, 5 mM MgCl_2_, 1% Triton X‐100, 100 µg/mL CHX) supplemented with a protease inhibitor cocktail and an RNase inhibitor, followed by incubation on ice for 15 min. After centrifugation at 13000 × g for 10 min, the supernatants (cytosolic fractions) were collected. Supernatants were then loaded onto 10%–50% sucrose gradients containing RNase inhibitor and centrifuged at 38000 rpm for 2 h using a Beckman SW41 Ti rotor. The sucrose gradient was separated into 12 fractions from the top, and absorbance at 260 nm was monitored continuously by a Piston Gradient Fractionator (Biocomp, Canada).

### 5.10 RNA‐seq

For RNA sequencing, cell lysis, mRNA reverse transcription, and cDNA amplification were performed using the Single Cell Full Length mRNA‐Amplification Kit (Vazyme, N712). Subsequently, cDNA fragmentation and adapter ligation were carried out with the TruePrep Flexible DNA Library Prep Kit for Illumina (Vazyme, TD504). All procedures were conducted in strict accordance with the manufacturer's protocols. The RNA‐seq library sequencing was performed by Novogene on Illumina platforms, generating 150 bp paired‐end reads.

### 5.11 RNA‐seq Data Analysis

Raw data (raw reads) of fastq format were first subjected to quality control using fastp software. In this step, clean data (clean reads) were obtained by removing reads containing adapters, reads containing poly‐N, and low‐quality reads from raw data. Paired‐end clean reads were aligned to the GRCm38 reference genome by using HISAT2 (version 2.2.1) [93]. FeatureCounts (version 2.0.6) [94] from the Subread package was used to count the reads mapped to each gene. Fragments per kilobase of transcript per million mapped reads (FPKM) values for each gene were subsequently calculated using an R script based on gene lengths and read counts.

### 5.12 Ribo‐seq Experiment, Library Preparation, and Sequencing

Ribosome profiling was carried out using the QEZ‐seq kit (Hangzhou NeoRibo Biotechnology Co., Ltd., Cat. R1024L) following the manufacturer's instructions. The QEZ‐seq workflow is based on an advanced ribosome footprinting protocol that enables high‐resolution mapping of ribosome‐protected mRNA fragments, as previously described [95]. In brief, cells were lysed under conditions that preserve polysomes, and ribosome–mRNA complexes were digested with RNase I to generate ribosome‐protected fragments (RPFs). The fragments were then purified, dephosphorylated, ligated to adaptors, and converted into cDNA libraries according to the QEZ‐seq protocol. The resulting libraries were quantified, pooled, and sequenced on an Illumina platform.

### 5.13 Ribo‐seq Analysis

Ribosome profiling (Ribo‐seq) data were analyzed using RiboMiner [81] with the code available at https://github.com/xryanglab/RiboMiner. After standard preprocessing of ribosome‐protected fragments (adapter trimming, quality filtering, and genome alignment), we applied RiboMiner to conduct data quality assessment (read length distribution, 3‐nt periodicity, and genomic mapping profiles), metagene analysis of ribosome footprint distributions, feature analysis of codon usage and amino acid properties, and comparative enrichment analysis across experimental conditions. All procedures followed the published RiboMiner pipeline with default parameters to ensure reproducibility.

### 5.14 Gene Ontology Analysis

GO analysis was performed using GO_KEGG_Analysis tools in Hiplot Pro (https://hiplot.com.cn/), a comprehensive web service for biomedical data analysis and visualization, or the David database (https://davidbioinformatics.nih.gov/).

### 5.15 Quantification and Statistical Analysis

Quantification and statistical analyses were performed using GraphPad Prism 9.0 software. Data are presented as mean ± SEM (standard error of the mean), unless otherwise stated. Details of statistical tests, sample sizes, and significance levels are indicated in the relevant figure legends. All experimental results were confirmed by three independent experiments, unless otherwise stated.

## Author Contributions

H.L., X.J., and Z.C. conceived and designed this study; J.X. was involved in conceptualization, the majority of experimentation, data collection, analysis, and writing – original draft, review & editing, with some helpful input from all authors. Y.H. and Y.C. performed most of the experiments. X.L. and T.L. analyzed the high‐throughput data; J.M., J.L.M., Y.G., S.L., and M.G. participated in the additional experiments during the revision and contributed significantly to manuscript refinement and the preparation of responses to reviewers’ comments. H.L., X.J., J.M., and Z.C. were responsible for supervision, project administration, and resources. All authors have read and approved the final version of the manuscript.

## Conflicts of Interest

The authors declare no conflicts of interest.

## Supporting information




**Supporting File 1**: advs76264‐sup‐0001‐SuppMat.pdf.

**Supporting File 2**: advs76264‐sup‐0002‐Table S1‐S5.zip.

## Data Availability

The data that support the findings of this study are openly available in the Gene Expression Omnibus (GEO) data repository under accession numbers GSE307441 and GSE307886, and in the ProteomeXchange consortium (via iProX) under accession number PXD068205.
