# Supplementary material for: eEF1G Orchestrates Translation to Ensure Meiotic Progression in Transcriptionally Quiescent Spermatocytes
Source: Adv Sci (Weinh). 2026 Jul 1;13(52):e76264. doi: 10.1002/advs.76264 (PMC13336642; doi:10.1002/advs.76264)
Supplement: Supplementary file 1 [file ADVS-13-e76264-s001.pdf]

Supporting Information

eEF1G orchestrates translation to ensure meiotic progression in transcriptionally quiescent spermatocytes

Jianze Xu[1,2,3,4,5,6†], Yuwei Hu[1,2,3,4,5,6,7†], Xukun Lu[1,2,3,4,5,6†], Yuling Cai[1,2,3,4,5,6,7†], Tongtong Li[1,2,3,4,5,6,7], Ming Gao[1,2,3,4,5,6], Jinlong Ma[1,2,3,4,5,6], Yuan Gao[1,2,3,4,5,6], Shangming Liu[7], Zi-Jiang Chen[1,2,3,4,5,6,7]*, Jing Meng[8]*, Hongbin Liu[1,2,3,4,5,6,7]*, Xiaohua Jiang[9]*.

†These authors contributed equally to this work

*Corresponding author

This PDF file includes:

Supplementary figures

Figure S1 - 16

Supplementary tables

Table S6 and S7

Description of Table S1 to S5 (Separate excel file)

Other Supplementary materials for this manuscript include the following:

Table S1 - S5

Supplementary figures

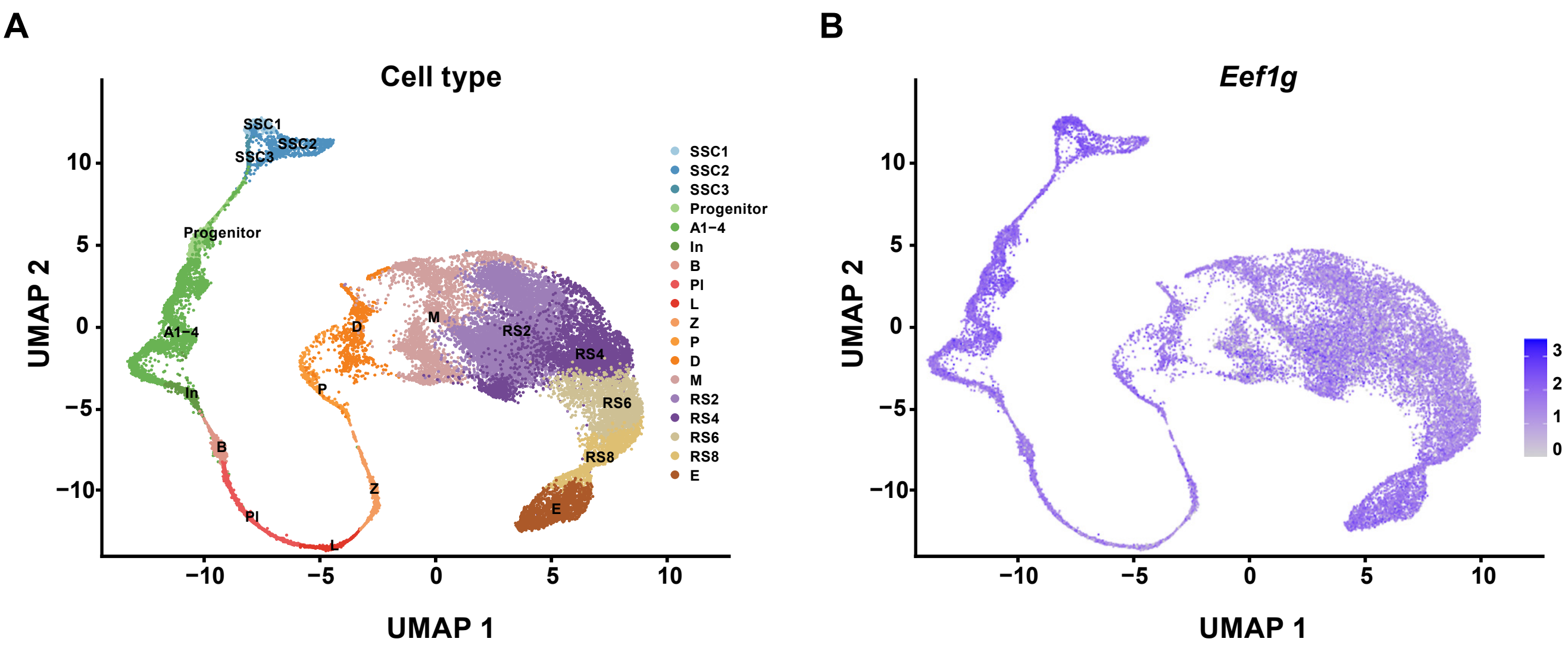

Figure S1. The expression pattern of *Eef1g* during spermatogenesis.

(A-B) t-SNE plots of mouse single-cell RNA sequencing (scRNA-seq) data showing *Eef1g* expression patterns. scRNA-seq datasets were obtained from the Male Health Atlas (http://malehealthatlas.cn/). SSC1, spermatogonial stem cell 1; SSC2, spermatogonial stem cell II; SSC3, A aligned spermatogonia III; Progenitor, spermatogonial progenitor cell; A1-4, A1-A4 spermatogonia; In, intermediate spermatogonia; B, type B spermatogonia; Pl, preleptotene; L, leptotene; Z, zygotene; P, pachytene; D, diplotene; M, metaphase; RS2, step1-2 round spermatid; RS4, step3-4 round spermatid; RS6, step5-6 round spermatid; RS8, step7-8 round spermatid; E, elongating spermatid.

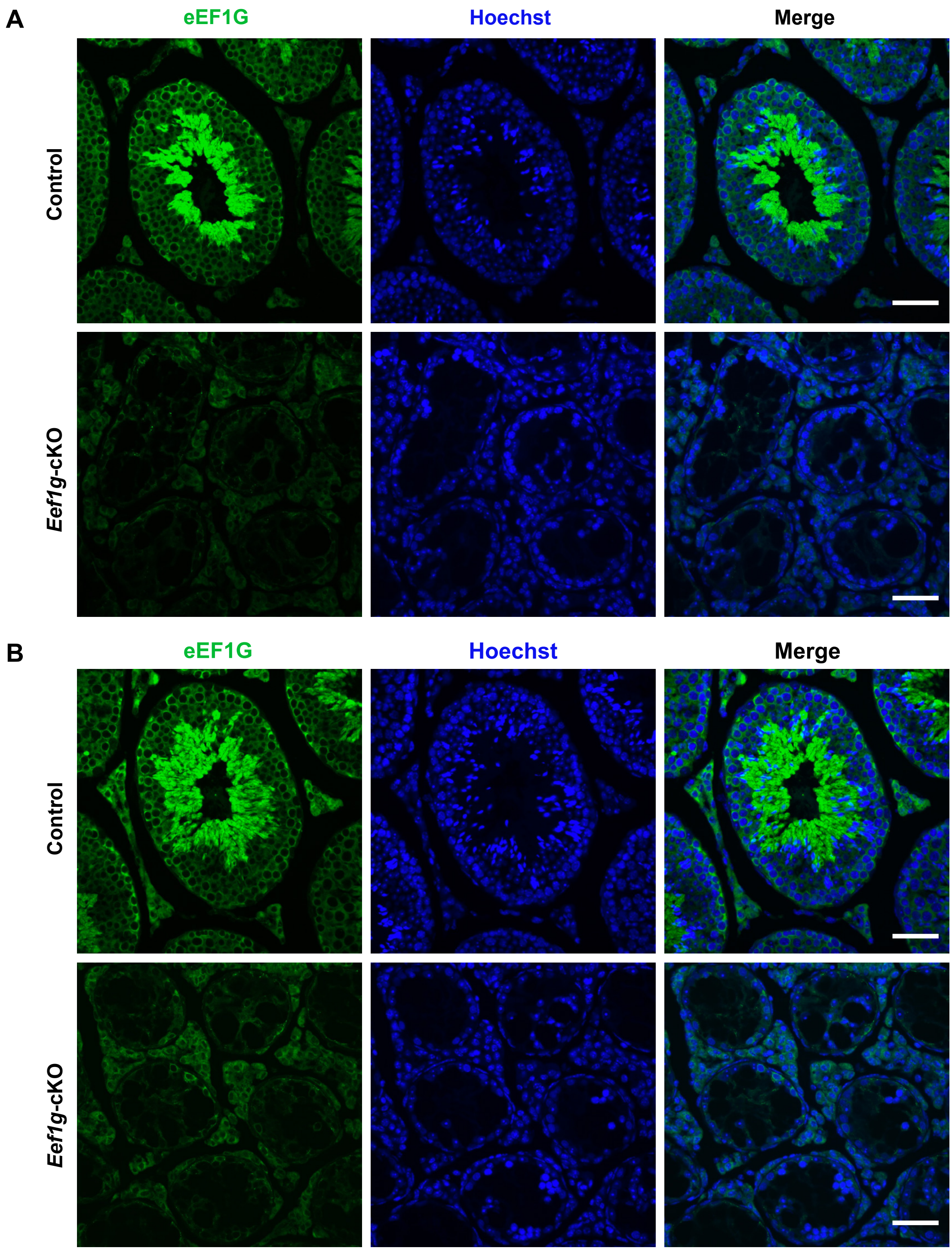

Figure S2. Deletion of *Eef1g* in germ cells.

(A) Immunofluorescence staining of eEF1G (antibody sourced from Abclonal) in mouse testis sections from control and *Eef1g*-cKO mice. Nuclei were stained with Hoechst (blue). Scale bars, 50 μm.

(B) Immunofluorescence staining of eEF1G (antibody sourced from Abmart) in mouse testis cross-sections from control and *Eef1g*-cKO mice. Nuclei were stained with Hoechst (blue). Scale bars, 50 μm.

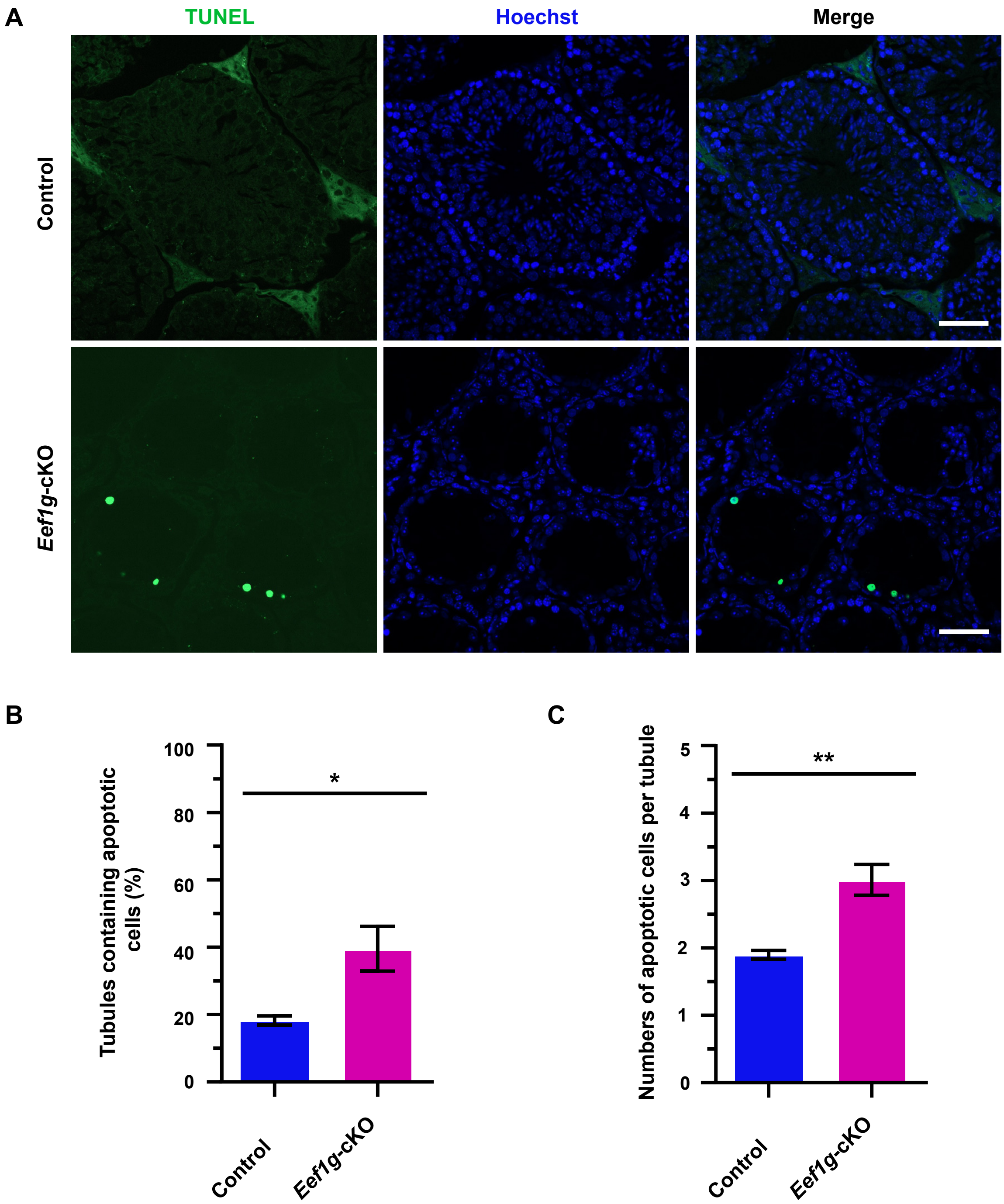

Figure S3. Germ cell-specific *Eef1g* ablation causes germ cell apoptosis.

(A) Testis sections from 10-week-old control and *Eef1g*-cKO mice were stained with TUNEL and Hoechst. Scale bars, 50 µm. **(B)** Percentage of TUNEL-positive tubules in testis sections from 10-week-old control and *Eef1g*-cKO mice (n = 4). **(C)** Quantification of TUNEL-positive cells per testicular tubule in 10-week-old control and *Eef1g*-cKO mice. Data are presented as the mean ± SEMs. $^*P < 0.05$; $^{**}P < 0.01$; Student's *t*-test.

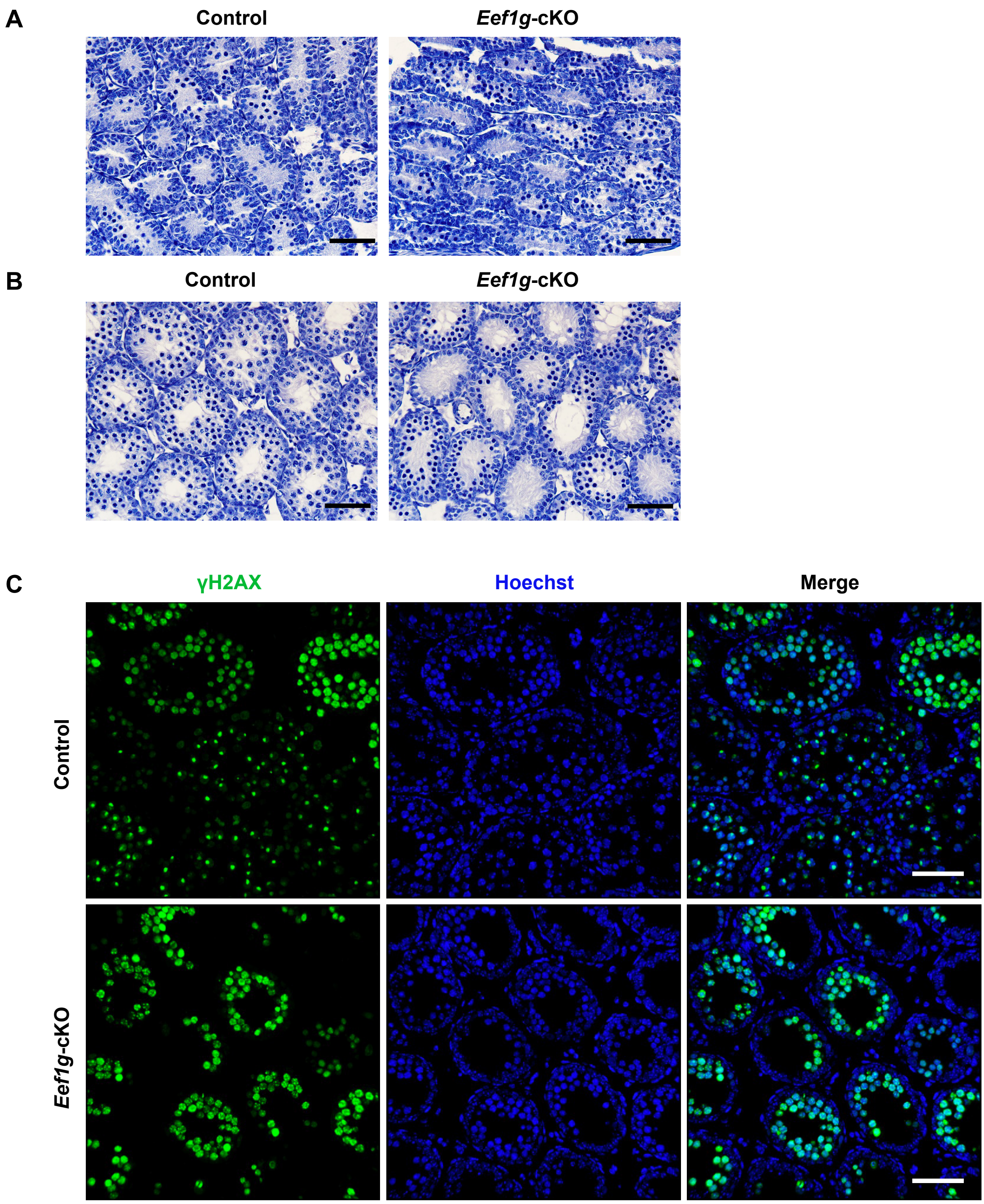

Figure S4. eEF1G deletion results in germ cell loss.

(A) Hematoxylin staining of testis sections from 10 dpp control and *Eef1g*-cKO mice. Scale bars, 50 µm. **(B)** Hematoxylin staining of testis sections from 14 dpp control and *Eef1g*-cKO mice. Scale bars, 50 µm. **(C)** Immunofluorescence staining of γH2AX in testis sections from 14 dpp control and *Eef1g*-cKO mice. Nuclei were stained with Hoechst (blue). Scale bars, 50 µm.

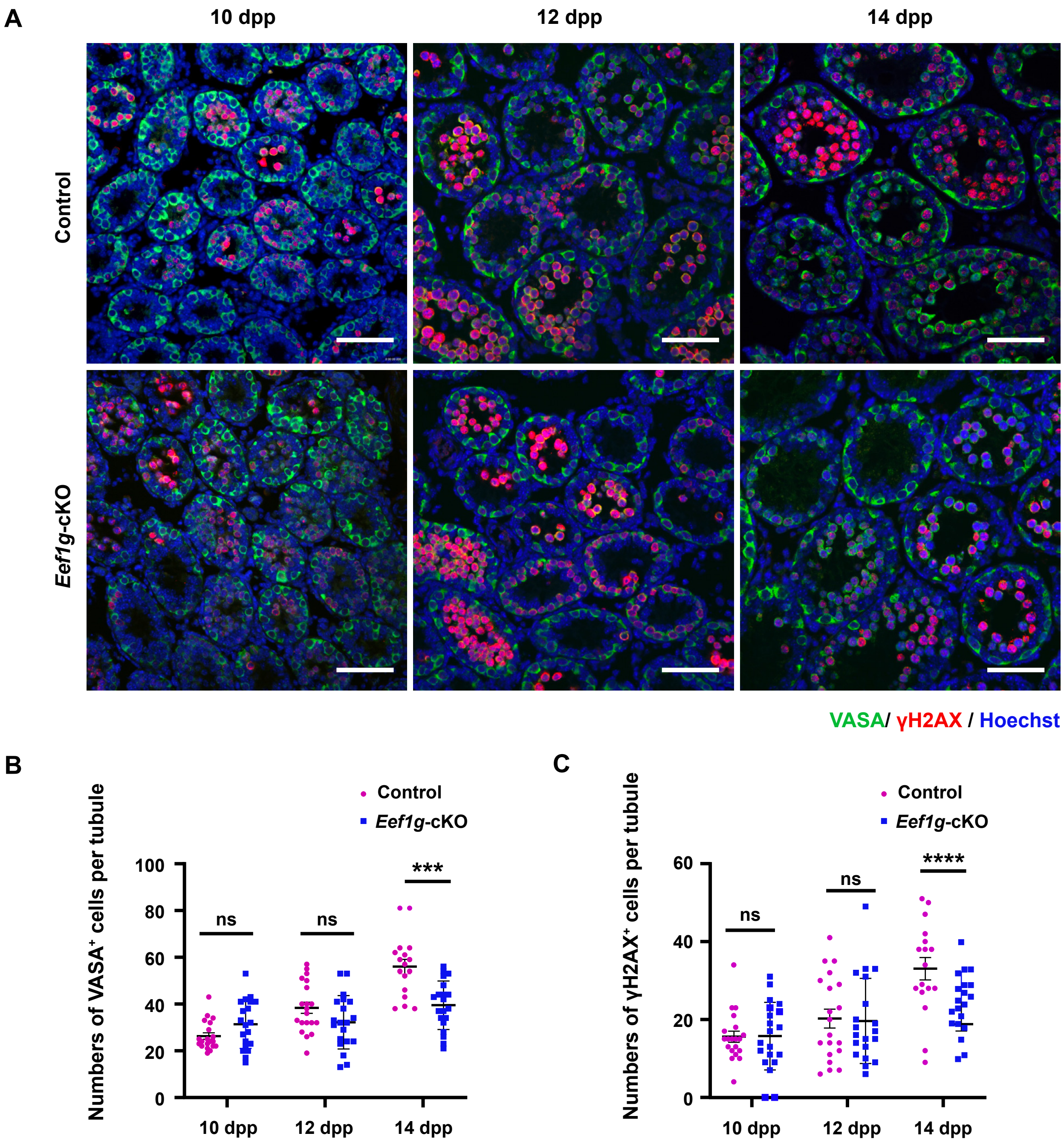

Figure S5. Spermatocytes decrease in the *Eef1g*-cKO mice after postnatal day 12.

(A) Immunostaining of VASA and γH2AX in testis sections from control and *Eef1g*-cKO mice at the indicated times. Nuclei were stained with Hoechst (blue). Scale bar, 50 μm. **(B)** Quantitative analysis of the number of $VASA^+$ cells per tubule. Student's *t*-test. ***$P < 0.001$. ns, not statistically significant. Data are presented as means ± SEMs. **(C)** Quantitative analysis of the number of $\gamma H2AX^+$ cells per tubule. Student's *t*-test. ****$P < 0.0001$. ns, not statistically significant. Data are presented as means ± SEMs.

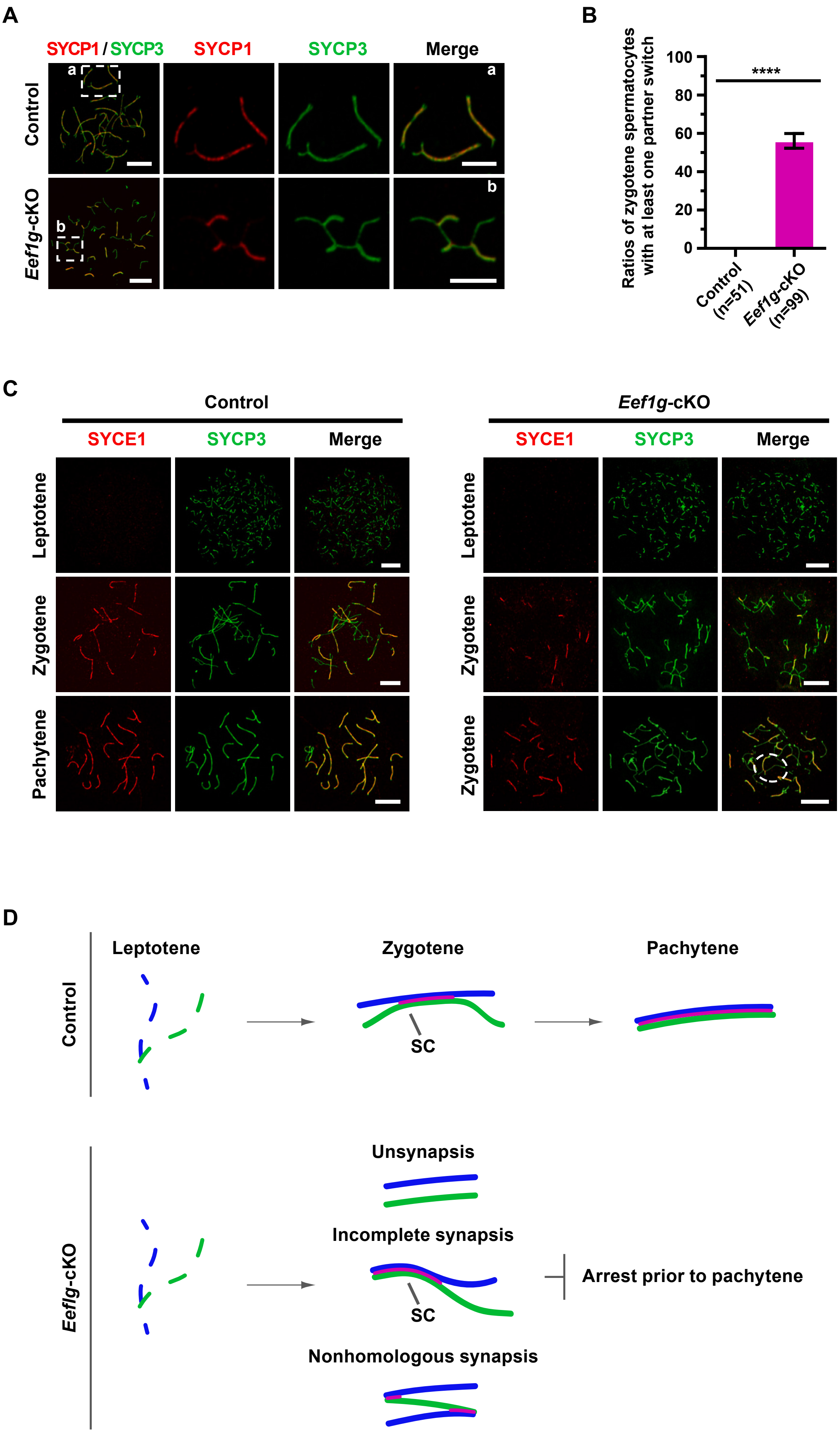

A
SYCP1 / SYCP3
SYCP1
SYCP3
Merge
Control
Eef1g-cKO
a
b
B
Ratios of zygotene spermatocytes with at least one partner switch
100
80
60
40
20
0
****
Control (n=51)
Eef1g-cKO (n=99)
C
Control
Eef1g-cKO
SYCE1
SYCP3
Merge
Leptotene
Zygotene
Pachytene
D
Leptotene
Zygotene
Pachytene
SC
Unsynapsis
Incomplete synapsis
Arrest prior to pachytene
Nonhomologous synapsis

Figure S6. *Eef1g*-deficient spermatocytes show synaptic defects.

(A) Representative zygotene spermatocytes from control and *Eef1g*-cKO mice stained with antibodies for SYCP1 (red) and SYCP3 (green). Magnified pictures highlight the partner switch in *Eef1g*-cKO spermatocytes. Scale bars, 10 µm (main panels) and 5 µm (insets). **(B)** The percentage of zygotene spermatocytes with at least one partner switch is shown (control, n = 51; *Eef1g*-cKO, n = 99; pooled from three mice per genotype). Scale bars: 10 µm (main panel), 5 µm (insets). 56.06 ± 3.82% (mean ± SEM) of *Eef1g*-cKO zygotene spermatocytes showed at least one partner switch. Unpaired two-tailed Student's *t*-test. ****$P < 0.0001$. **(C)** Representative images of spermatocyte spreads from 10-week-old control and *Eef1g*-cKO mice stained for SYCE1 (red) and SYCP3 (green). Scale bars: 10 µm. **(D)** Schematic summary of the synapsis defects in *Eef1g*-cKO spermatocytes. *Eef1g*-deficient spermatocytes exhibit synapsis defects such as asynapsis (completely separated chromosomes), incomplete synapsis, and aberrant non-homologous synapsis.

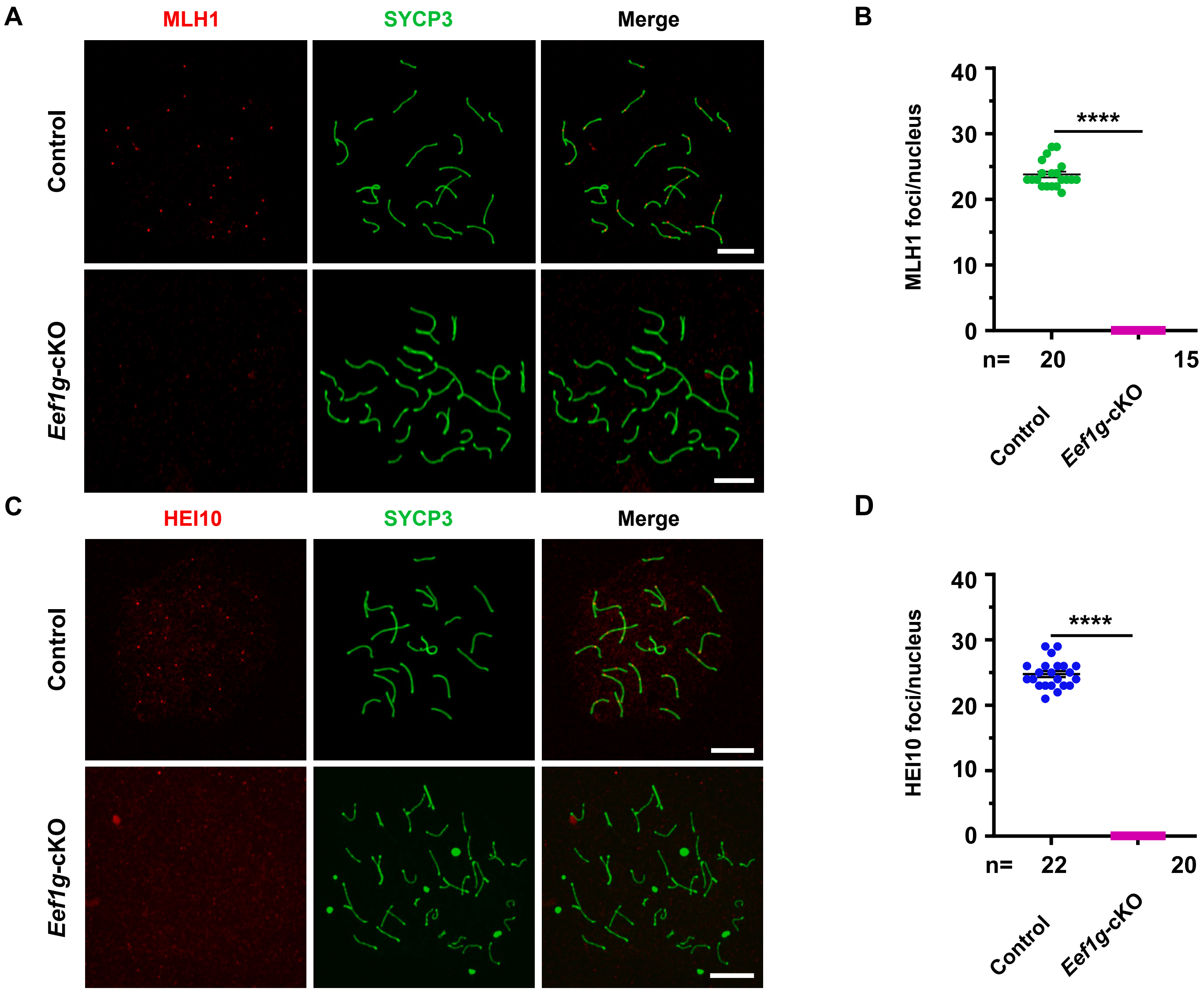

Figure S7. *Eef1g* deletion in germ cells causes defects in meiotic recombination.

(A) Representative spermatocyte spreads from adult control and *Eef1g*-cKO mice stained for SYCP3 (green) and MLH1 (red). Scale bars: 10 μm. **(B)** Quantification of MLH1 foci in spermatocytes. n, the total number of cells analyzed from two animals per genotype. The bars represent mean ± SEMs. ****$P < 0.0001$; Student' s *t*-test. **(C)** Representative spreads of spermatocytes from adult control and *Eef1g*-cKO mice stained for SYCP3 (green) and HEI10 (red). Scale bars: 10 μm. **(D)** Quantification of HEI10 foci in spermatocytes. n, the total number of cells analyzed from three animals per genotype. The bars represent mean ± SEMs. ****$P < 0.0001$; Student's *t*-test.

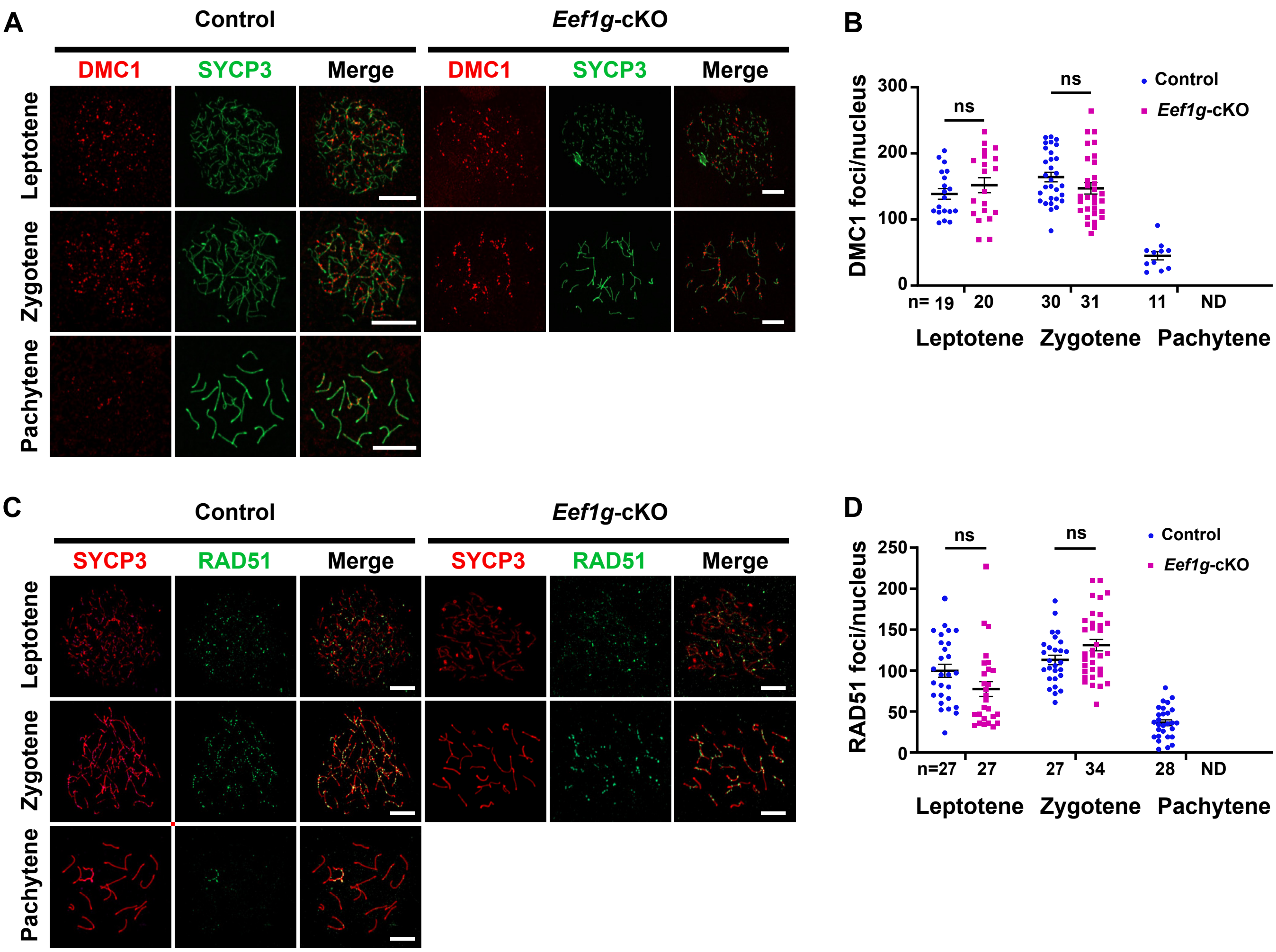

Figure S8.DMC1 and RAD51 signals remain normal in *Eef1g*-deficient spermatocytes.

(A) Representative spread spermatocytes from adult control and *Eef1g*-cKO mice were immunostained for SYCP3 and DMC1. Leptotene, zygotene, and pachytene spermatocytes are shown. Scale bars, 10 µm. **(B)** Quantification of DMC1 foci in spread spermatocytes at the indicated substages. n, the total number of cells analyzed from three animals per genotype. The bars indicate the mean ± SEMs. P values were calculated by the Student' s *t*-test. ns, not significant ($P > 0.05$). **(C)** Representative spread spermatocytes from adult control and *Eef1g*-cKO mice were immunostained for SYCP3 and RAD51. Leptotene, zygotene, and pachytene spermatocytes are shown. Scale bars, 10 µm. **(D)** Quantification of RAD51 foci in spread spermatocytes at the indicated substages. n, the total number of cells analyzed from three animals per genotype. The bars indicate the mean ± SEMs. P values were calculated by the Student' s *t*-test. ns, not significant ($P > 0.05$).

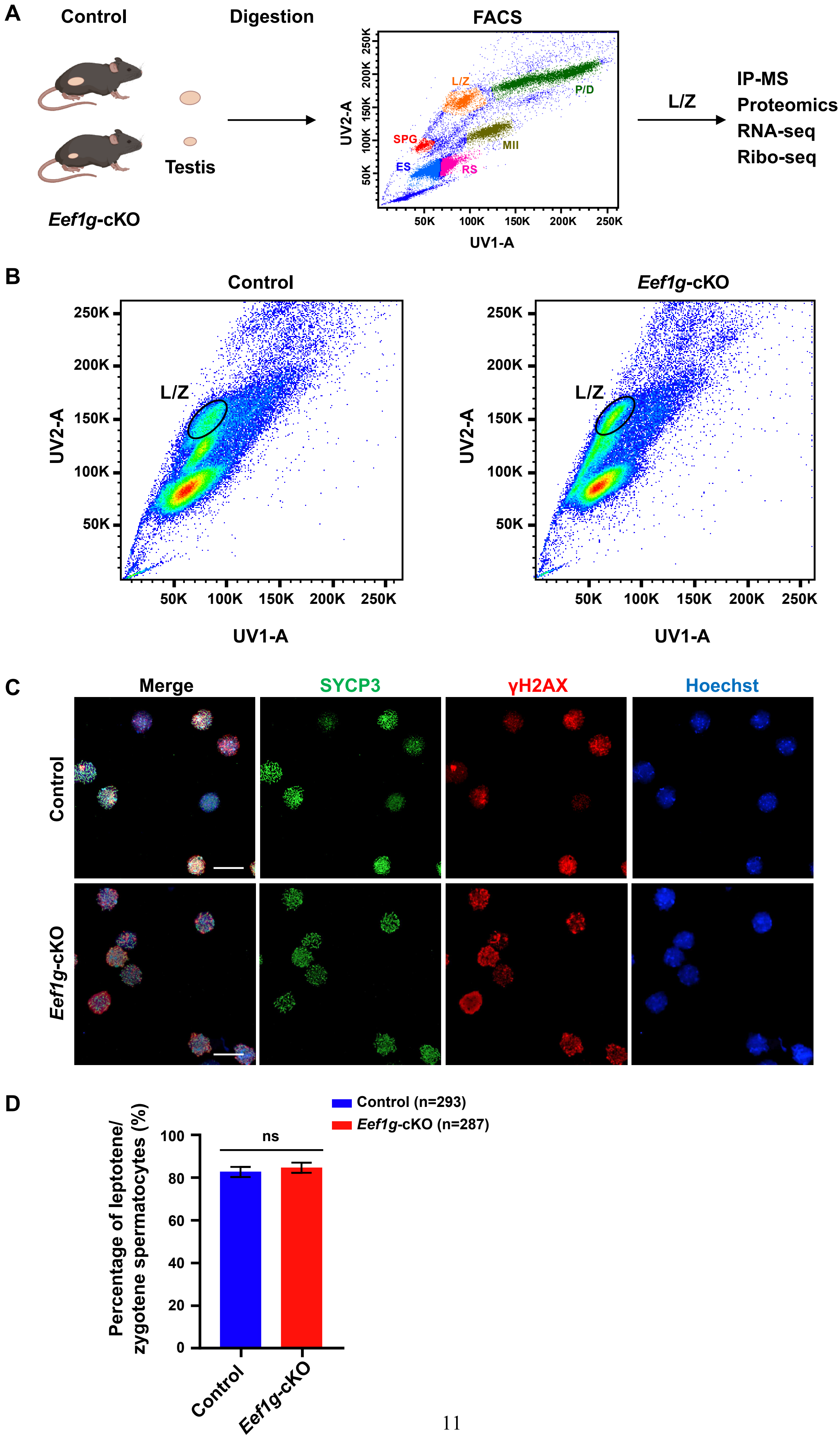
A
Control
Digestion
FACS
Testis
Eef1g-cKO
UV2-A
UV1-A
L/Z
P/D
SPG
MII
ES
RS
L/Z
IP-MS
Proteomics
RNA-seq
Ribo-seq
B
Control
Eef1g-cKO
C
Merge
SYCP3
γH2AX
Hoechst
Control
Eef1g-cKO
D
Control (n=293)
Eef1g-cKO (n=287)
Percentage of leptotene/zygotene spermatocytes (%)
ns
Control
Eef1g-cKO

Figure S9. Isolation of leptotene/zygotene spermatocytes from 12 dpp mouse testes via FACS.

(A) Schematic illustration of eEF1G immunoprecipitation-mass spectrometry (IP-MS), proteome analysis, RNA sequence (RNA-seq), and ribosome sequencing (Ribo-seq) experiments using leptotene and zygotene spermatocytes isolated via fluorescence-activated cell sorting (FACS). **(B)** Flow cytometry analysis of the spermatogenic cell population in 12 dpp control and *Eef1g*-cKO testes. L/Z indicates leptotene/zygotene spermatocytes. **(C)** FACS-purified leptotene/zygotene spermatocytes from 12 dpp control and *Eef1g*-cKO testes were stained for SYCP3 (green) and γH2AX (red). Nuclei were stained with Hoechst (blue). Scale bar: 20 μm. **(D)** Percentage of leptotene/zygotene spermatocytes in FACS-purified populations from control and *Eef1g*-cKO testes. Student's *t*-test; ns,not significant (P > 0.05). Data represent mean ± SEMs (n = 3).

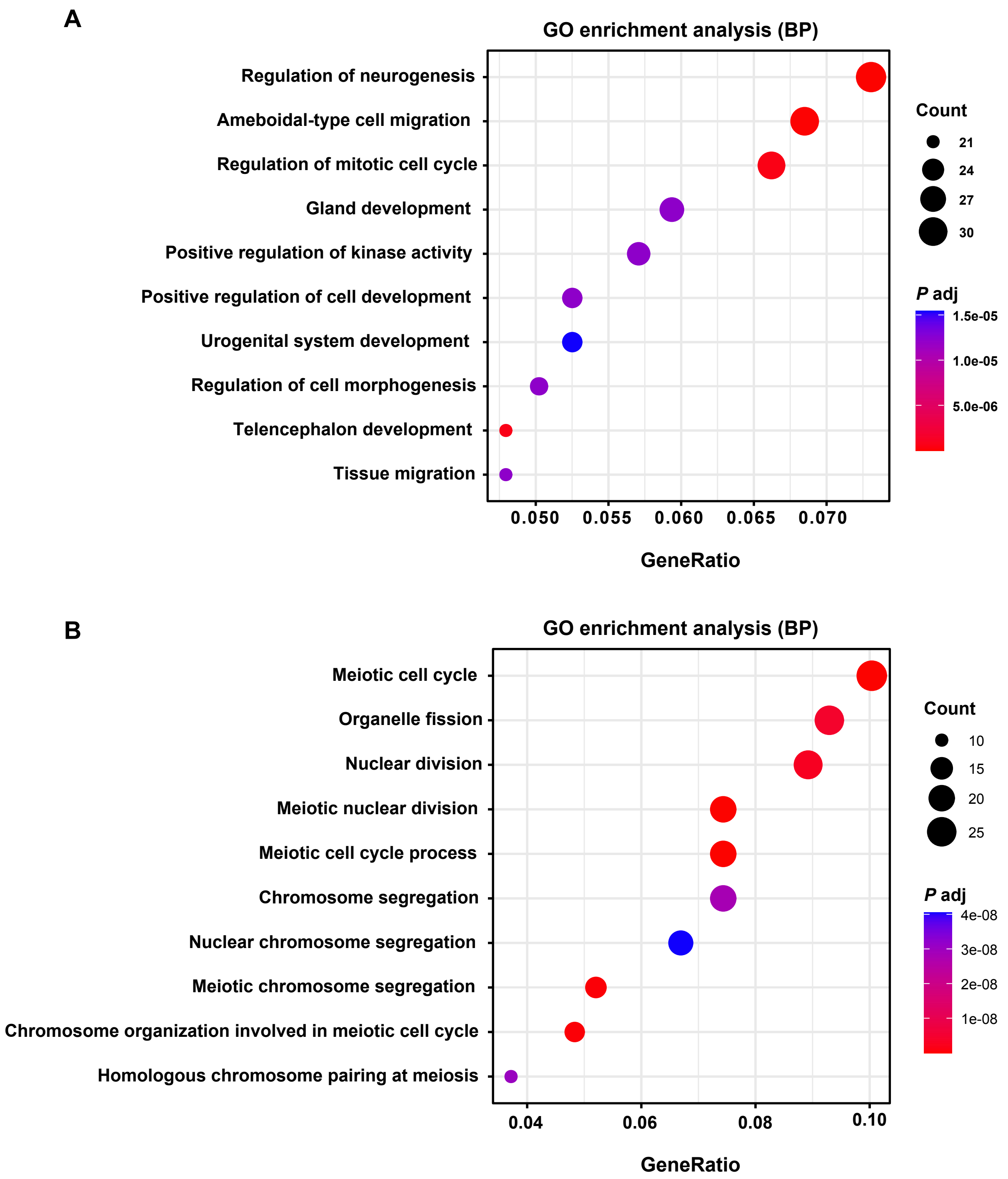

Figure S10. GO analysis of transcriptionally altered genes in *Eef1g*-cKO spermatocytes. **(A)** Gene ontology enrichment analysis of downregulated genes in *Eef1g*-deficient leptotene and zygotene spermatocytes. **(B)** Gene ontology enrichment analysis of upregulated genes in *Eef1g*-deficient leptotene and zygotene spermatocytes.

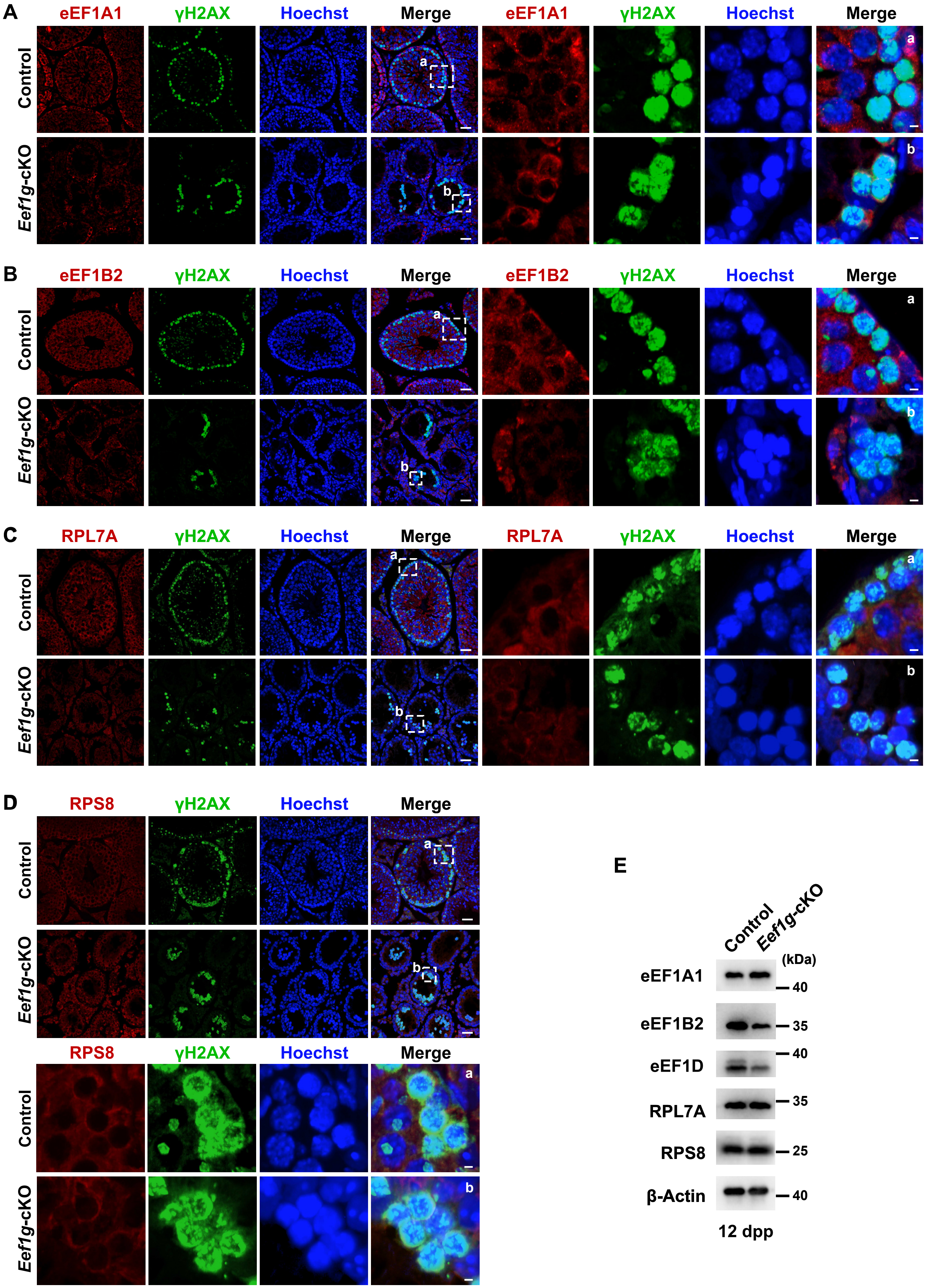
A
eEF1A1
γH2AX
Hoechst
Merge
Control
Eef1g-cKO
a
b
B
eEF1B2
C
RPL7A
D
RPS8
E
Control
Eef1g-cKO
(kDa)
eEF1A1
40
eEF1B2
35
eEF1D
40
RPL7A
35
RPS8
25
β-Actin
40
12 dpp

Figure S11. Germ cell-specific *Eef1g* deletion reduces eEF1B2 and eEF1D protein levels.

(A) Immunofluorescence staining of eEF1A1 (red) and γH2AX (green) in mouse testis sections from 10-week-old control and *Eef1g*-cKO mice. Nuclei were stained with Hoechst (blue). Scale bars, 20 μm (main panels) and 3 μm (insets). **(B)** Immunofluorescence staining of eEF1B2 (red) and γH2AX (green) in mouse testis sections from 10-week-old control and *Eef1g*-cKO mice. Nuclei were stained with Hoechst (blue). Scale bars, 20 μm (main panels) and 3 μm (insets). **(C)** Immunofluorescence staining of RPL7A (red) and γH2AX (green) in mouse testis sections from 10-week-old control and *Eef1g*-cKO mice. Nuclei were stained with Hoechst (blue). Scale bars, 20 μm (main panels) and 3 μm (insets). **(D)** Immunofluorescence staining of RPS8 (red) and γH2AX (green) in mouse testis sections from 10-week-old control and *Eef1g*-cKO mice. Nuclei were stained with Hoechst (blue). Scale bars, 20 μm (main panels) and 3 μm (insets). **(E)** Representative Western blot showing the protein levels of eEF1A1, eEF1B2, eEF1D, RPL7A, and RPS8 in testes of 12 dpp *Eef1g*-cKO and control mice. β-Actin was used as a loading control.

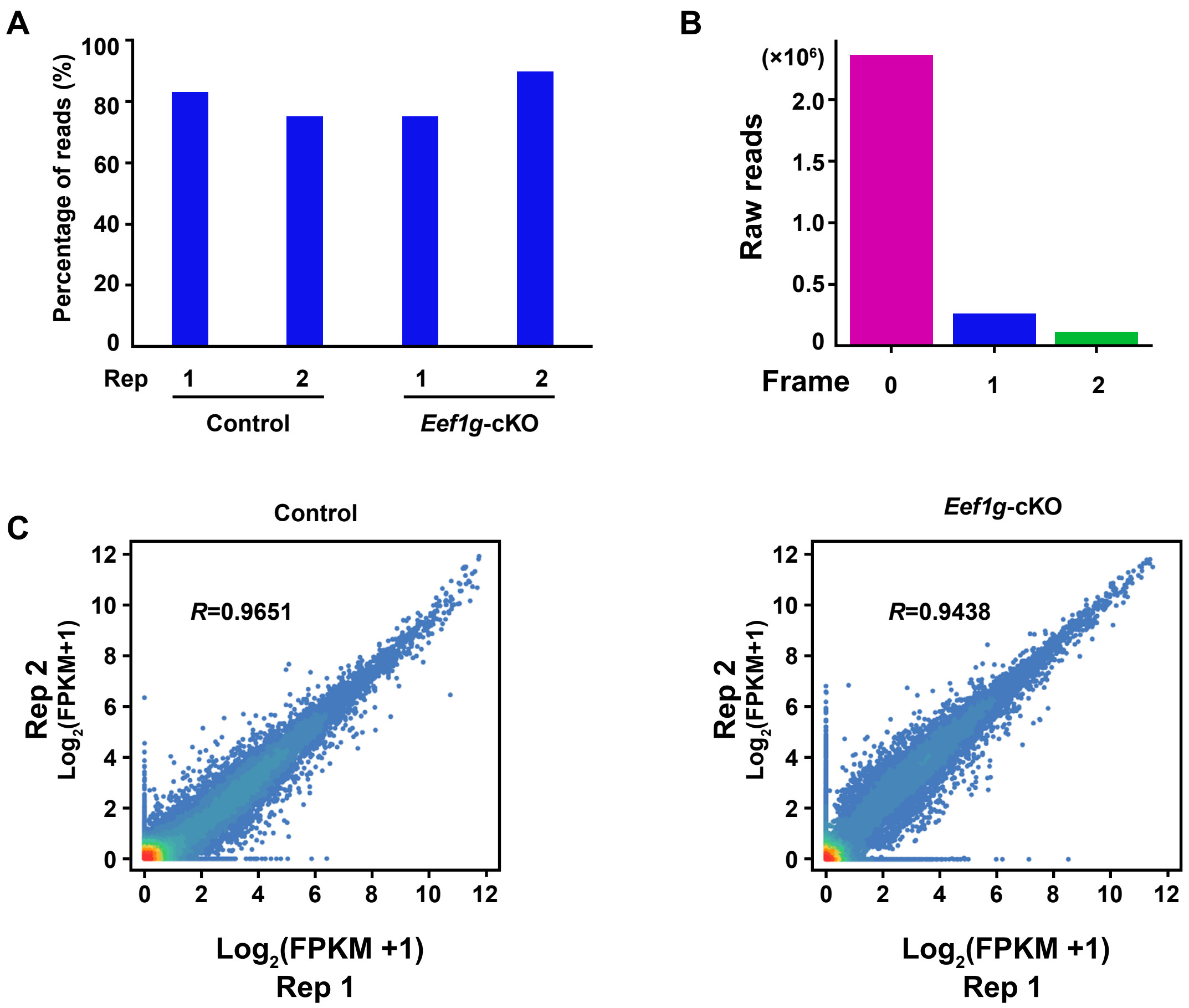

Figure S12. Results of quality control (QC).

(A) The percentage of reads exhibiting 3-nucleotide periodicity across different groups. **(B)** Reads mapped to distinct reading frames. Representative sample (Control Rep1) shown; all four replicates consistent (frame 0 enrichment). **(C)** Scatter plots for comparing the Ribo-seq data generated in this study, with Spearman correlation coefficients indicated.

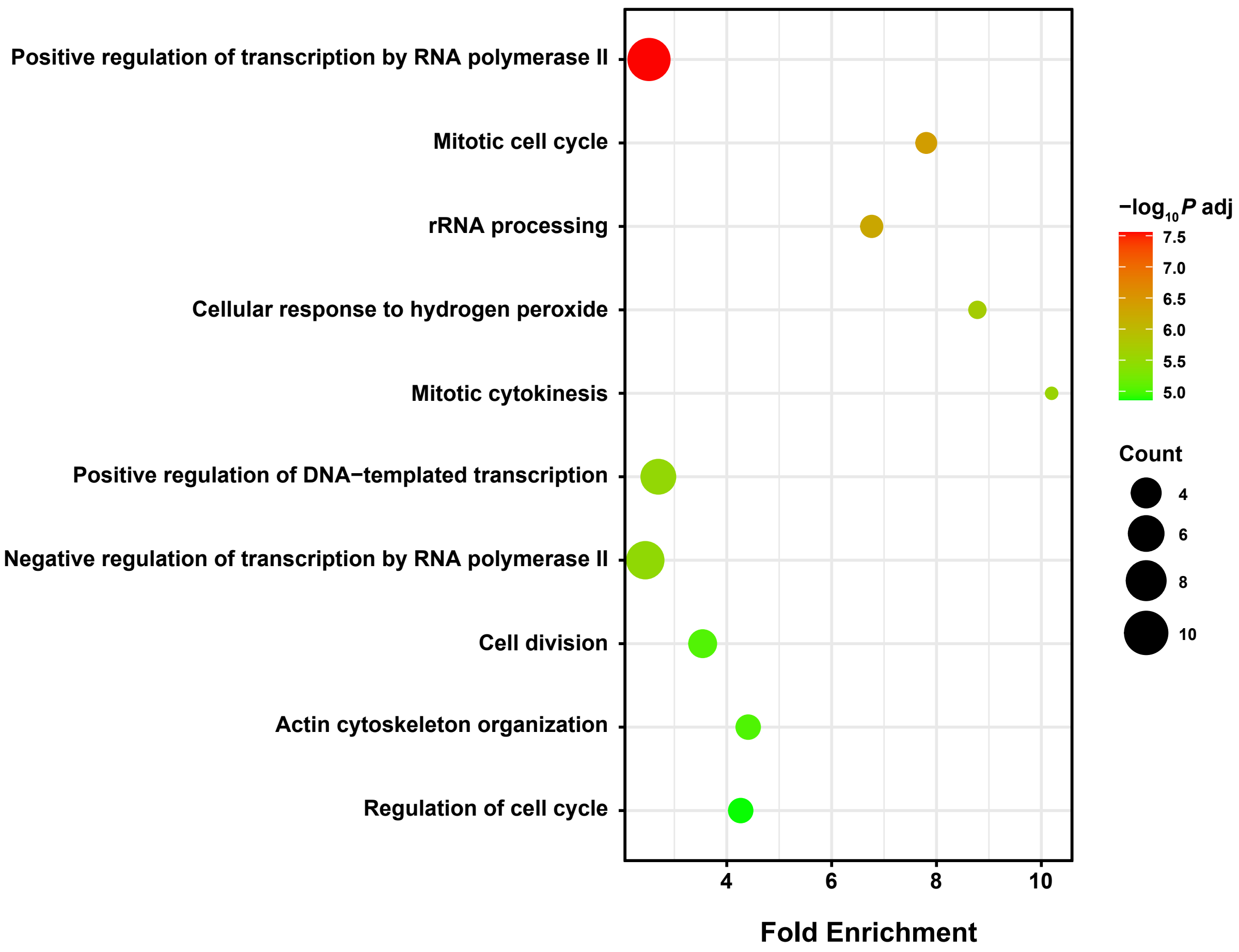

Figure S13. GO enrichment analysis of downregulated ribosome-protected fragments (RPFs) in *Eef1g*-cKO spermatocytes.

GO analysis of downregulated ribosome-protected fragments (RPFs) in leptotene and zygotene spermatocytes from *Eef1g*-cKO mice.

A

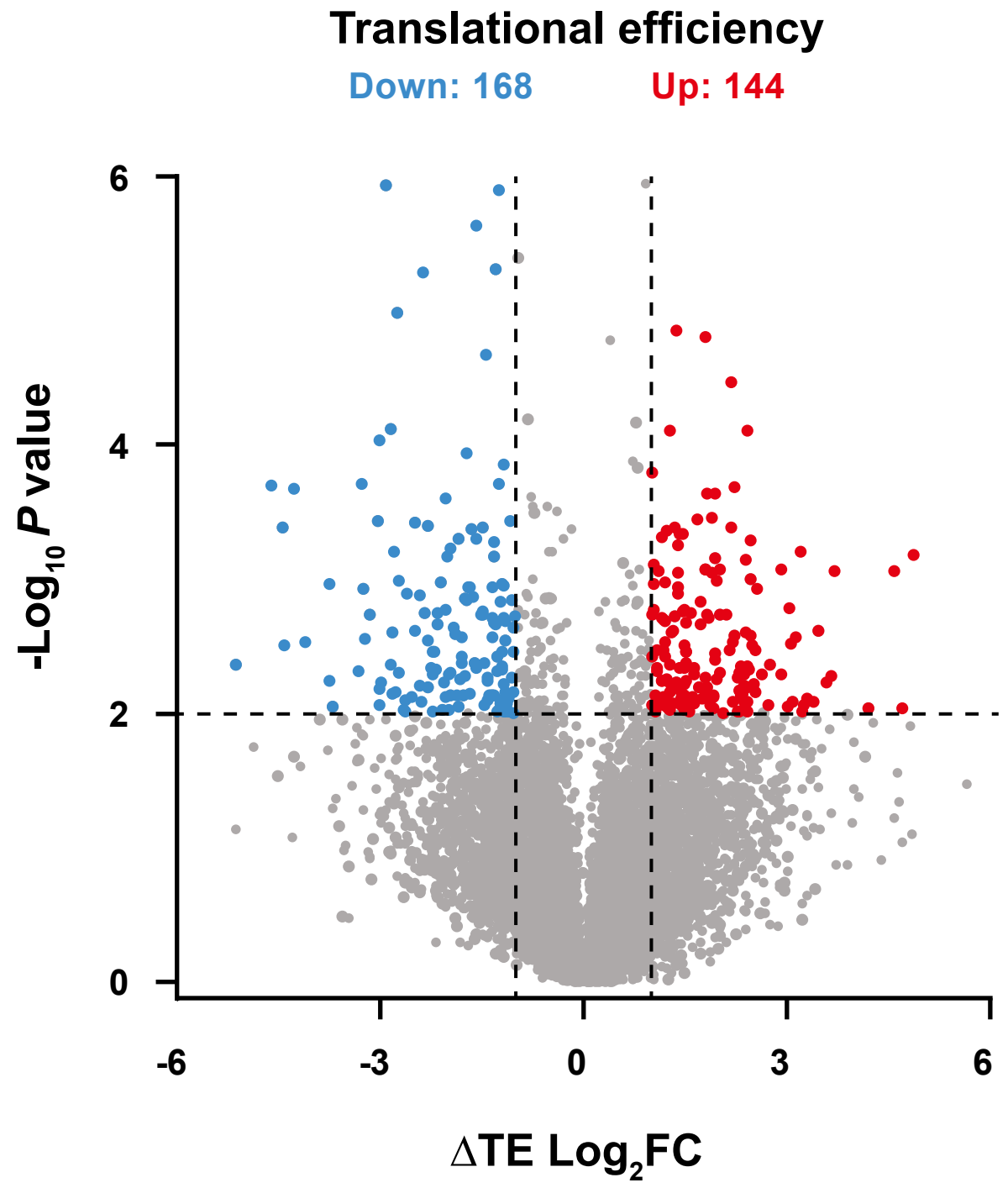
Translational efficiency
Down: 168
Up: 144
-Log10 P value
ΔTE Log2FC
6
4
2
0
-6
-3
0
3
6

B

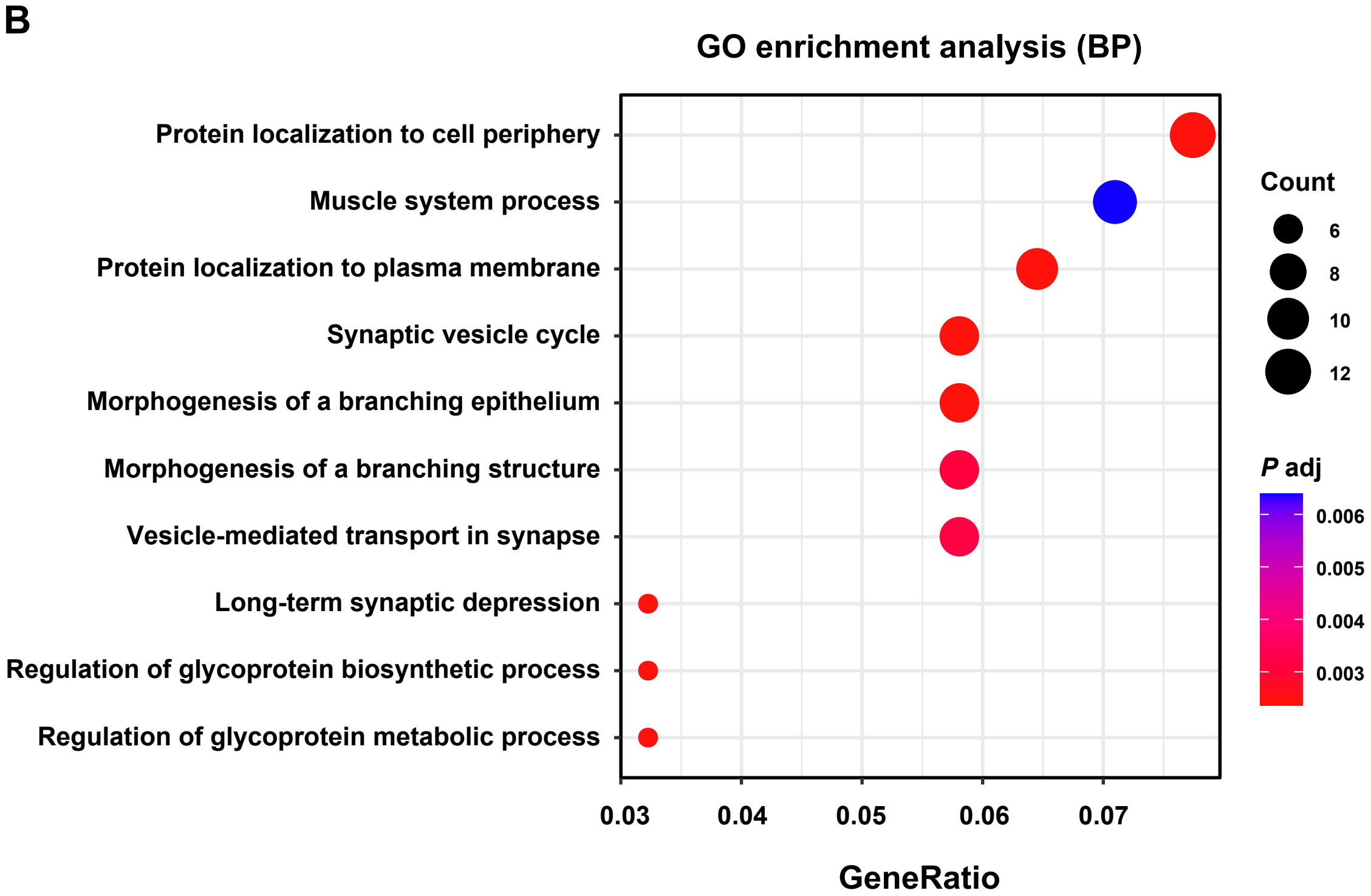
GO enrichment analysis (BP)
Protein localization to cell periphery
Muscle system process
Protein localization to plasma membrane
Synaptic vesicle cycle
Morphogenesis of a branching epithelium
Morphogenesis of a branching structure
Vesicle-mediated transport in synapse
Long-term synaptic depression
Regulation of glycoprotein biosynthetic process
Regulation of glycoprotein metabolic process
0.03
0.04
0.05
0.06
0.07
GeneRatio
Count
6
8
10
12
P adj
0.006
0.005
0.004
0.003

Figure S14. Translational efficiency changes in *Eef1g*-cKO spermatocytes are not enriched for meiotic genes.

(A) Volcano plot showing changes in translational efficiency (ΔTE) between *Eef1g*-cKO and control spermatocytes. The x axis represents the $\log_2$ fold change in translational efficiency (Δ TE $\mathrm{Log_2FC}$), and the y axis represents the $-\log_{10}$ *P* value. Genes with significantly increased translational efficiency (ΔTE > 0) are labeled as " Up " (n = 144), and those with significantly decreased translational efficiency (ΔTE < 0) are labeled as " Down " (n = 168). The dashed line indicates the significance threshold. **(B)** GO enrichment analysis of genes with increased translational efficiency (ΔTE > 0, n = 144) in *Eef1g*-cKO spermatocytes was compared to control spermatocytes. The dot plot shows selected significantly enriched biological processes, ordered by GeneCount (number of genes in each term). Note: No significant GO terms were enriched among genes with decreased translational efficiency (ΔTE < 0, n = 168)

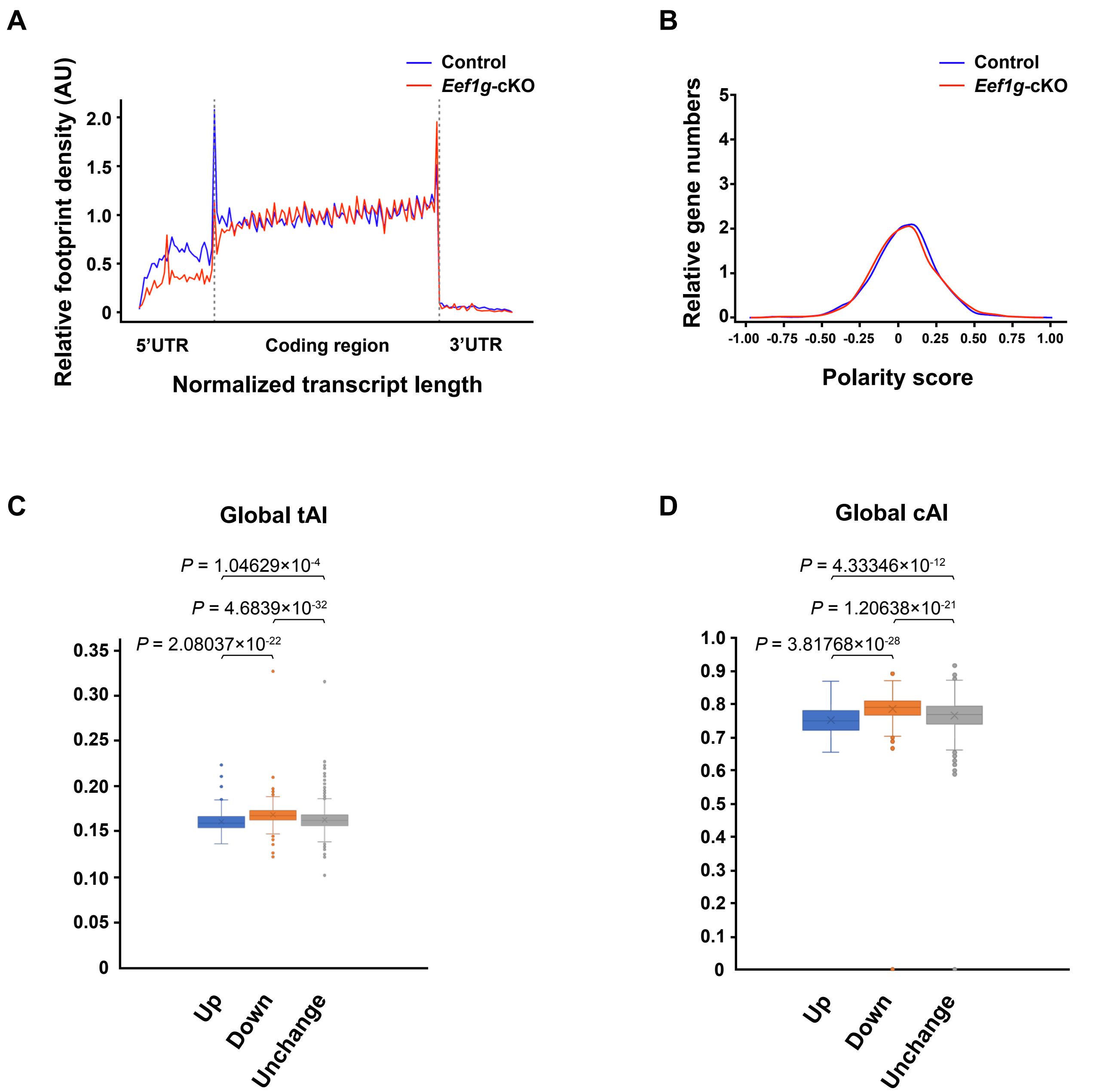

Figure S15. Ribo-seq analysis of translation features in control and *Eef1g*-deficient spermatocytes.

(A) The global distribution pattern of reads along transcripts between control and *Eef1g*-cKO spermatocytes. **(B)** Distribution of polarity scores between control and *Eef1g*-cKO spermatocytes. **(C)** Distribution of the global tRNA adaptation index (tAI) across different gene sets. *P* values were calculated using a two-tailed Student' s *t*-test and are indicated on the graphs. **(D)** Distribution of the global codon adaptation index (cAI) across different gene sets. *P* values were calculated using a two-tailed Student's *t*-test and are indicated on the graphs.

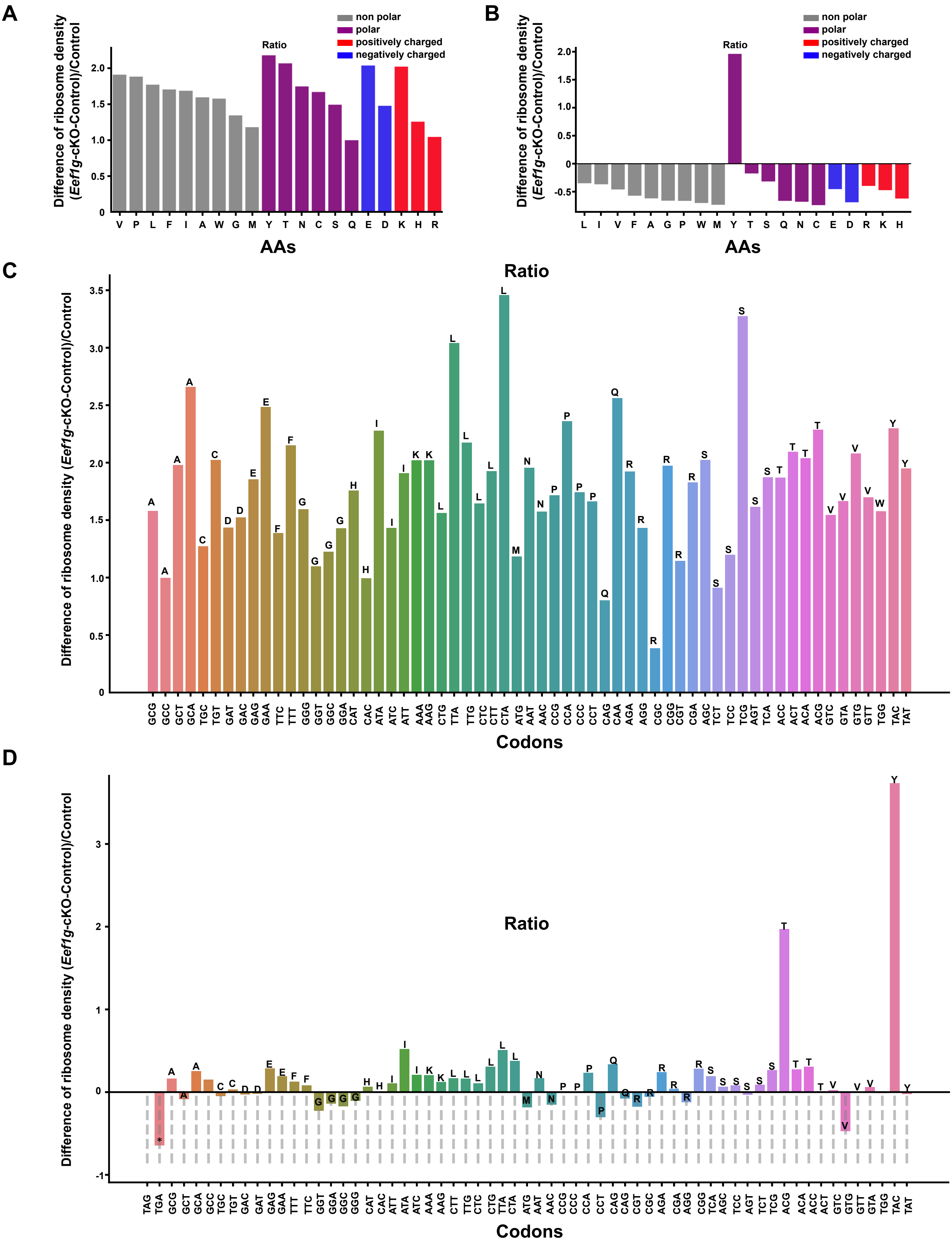
A
Difference of ribosome density (Eef1g-cKO-Control)/Control
Ratio
non polar
polar
positively charged
negatively charged
V P L F I A W G M Y T N C S Q E D K H R
AAs
B
Difference of ribosome density (Eef1g-cKO-Control)/Control
Ratio
non polar
polar
positively charged
negatively charged
L I V F A G P W M Y T S Q N C E D R K H
AAs
C
Ratio
Difference of ribosome density (Eef1g-cKO-Control)/Control
GCG GCC GCT GCA TGC TGT GAT GAC GAG GAA TTC TTT GGG GGT GGC GGA CAT CAC ATA ATC ATT AAA AAG CTG TTA TTG CTC CTT CTA ATG AAT AAC CCG CCA CCC CCT CAG CAA AGA AGG CGC CGG CGT CGA AGC TCT TCC TCG AGT TCA ACC ACT ACA ACG GTC GTA GTG GTT TGG TAC TAT
Codons
D
Ratio
Difference of ribosome density (Eef1g-cKO-Control)/Control
TAG TGA GCG GCT GCA GCC TGC TGT GAC GAT GAG GAA TTT TTC GGT GGA GGC GGG CAT CAC ATT ATA ATC AAA AAG CTT TTG CTC CTG TTA CTA ATG AAT AAC CCG CCC CCA CCT CAG CAG CGT CGC AGA CGA AGG CGG TCA AGC TCC AGT TCT TCG ACA ACC ACT GTC GTG GTT GTA TGG TAC TAT
Codons

Figure S16. Ribosome density across different amino acids and codons.

(A) Fold change in ribosome density between *Eef1g*-cKO samples and control samples for different amino acids within upregulated gene sets. **(B)** Fold change in ribosome density between *Eef1g*-cKO samples and control samples for different amino acids within downregulated gene sets. **(C)** Fold change in ribosome density between *Eef1g*-cKO samples and control samples for codons within up-regulated gene sets. **(D)** Fold change in ribosome density between *Eef1g*-cKO samples and control samples for codons within downregulated gene sets.

Supplementary tables

Table S6 Primers used in this study.

Primer names	Sequences (5'-3')
Eef1g-5wt-tF1	AGGGTTGCTAATTCGCCATG
Eef1g-3wt-tR1A	GTAGGTGGGTCAAAACTGAGCC
Eef1g-3wt-tR1	TGACCAGGCTAAATGAGTGAGTG
Stra8-GFPcre–F	ACTCCAAGCACTGGGCAGAA
Stra8-GFPcre-R1	GCCACCATAGCAGCATCAAA
Stra8-GFPcre-R2	CGTTTACGTCGCCGTCCAG

Table S7 Antibodies used in this study

Antibodies	Sources	Identifiers
Anti-SYCP3 Antibody	Abcam	Cat# Ab97672<br>RRID: AB_10678841
Anti-SYCP1 Antibody	Abcam	Cat# Ab15090<br>RRID: AB_301636
Anti-SYCP1 Antibody	Novus Biologicals	Cat# NB300-229<br>RRID: AB_10002742
Anti-β-Actin Antibody	Proteintech	Cat# 66009-1-Ig<br>RRID: AB_2687938
Anti-GAPDH Antibody	Proteintech	Cat# 60004-1-Ig<br>RRID: AB_2107436
Anti-RPL4 Antibody	Proteintech	Cat# 67028-1-Ig<br>RRID: AB_2882343
Anti-DDX4 Antibody	Abcam	Cat# ab27591<br>RRID: AB_11139638
Anti-RPS8 Antibody	Proteintech	Cat# 18228-1-AP<br>RRID: AB_10666168
Anti-RPL7A Antibody	Proteintech	Cat# 15340-1-AP<br>RRID: AB_2254051

Anti-EEF1A1 Antibody	Proteintech	Cat# 81377-1-RR<br>RRID: AB_2935549
Anti-EEF1B2 Antibody	Abclonal	Cat# A6580<br>RRID: AB_2863531
Anti-EEF1D Antibody	Abclonal	Cat# A2509<br>RRID: AB_2764400
Anti-EEF1G antibody	Abclonal	Cat# A8744<br>RRID: AB_2863600
Anti-EEF1G antibody	Abmart	Cat# T59970M
Anti-gamma H2A.X (phospho S139)	Abcam	Cat# Ab81299<br>RRID: AB_1640564
Anti-p-Histone H2A.X (Ser139) Antibody	Merck	Cat# 05-636<br>RRID: AB_309864
Anti-SYCE1 Polyclonal antibody	Proteintech	Cat# 11063-1-AP<br>RRID: AB_2197057
Anti-MLH1 Recombinant antibody	Proteintech	Cat# 11697-1-AP<br>RRID: AB_2145604
Anti-TEX12 Polyclonal antibody	Proteintech	Cat# 17068-1-AP<br>RRID: AB_2201875
Anti-HIST1H1T Polyclonal antibody	Proteintech	Cat# 18188-1-AP<br>RRID: AB_2878515
Anti-RAD51 Polyclonal antibody	Proteintech	Cat# 14961-1-AP<br>RRID: AB_2177083
Anti-DMC1 Polyclonal antibody	Proteintech	Cat# 13714-1-AP<br>RRID: AB_2091076
Anti-HORMAD1 Polyclonal antibody	Proteintech	Cat# 28719-1-AP<br>RRID: AB_2881199
Anti-MSH4 antibody	Abclonal	Cat# A8556<br>RRID: AB_2770446
Anti-SHOC1 antibody	From Professor Chao Yu	
Anti-RNF212 antibody	From Professor Mengcheng Luo	
Anti-HEI10 antibody	Home-made	
Anti-REC8 antibody	Abcam	Cat# ab192241

Anti-HSPA2 antibody	Proteintech	Cat# 12797-1-AP<br>RRID: AB_2119687
Anti-ATR antibody	Proteintech	Cat# 19787-1-AP<br>RRID:AB_10639516
Anti-TEX11 Polyclonal antibody	Proteintech	Cat# 17266-1-AP<br>RRID: AB_2201874
Lectin PNA From Arachis hypogaea (peanut), Alexa Fluor™ 594 Conjugate	Thermo Fisher Scientific	Cat# L32459

Description of Table S1-S5.

Table S1 Proteomics analysis of control and *Eef1g*-cKO leptotene and zygotene spermatocytes. Proteomic analyses identified 1,001 differentially expressed proteins ($P < 0.05$, $|\log_2$ fold change$| > |0.585|$, n = 3), including 89 upregulated and 912 downregulated proteins

Table S2 RNA sequencing (RNA-seq) analysis of control and *Eef1g*-cKO leptotene and zygotene spermatocytes. 303 genes were upregulated and 488 genes were downregulated ($P < 0.05$, $|\log_2$ fold change$| > |1|$, n = 3).

Table S3 Proteins that interact with eEF1G detected by IP-MS in WT leptotene and zygotene spermatocytes. MS results identified 58 eEF1G-interacting proteins, among which 31 were ribosomal proteins (including 27 large ribosomal subunits and 4 small ribosomal subunits), and 27 were RNA-binding proteins.

Table S4 Ribosome profiling (Ribo-seq) of control and *Eef1g*-cKO leptotene and zygotene spermatocytes. 502 genes with increased and 451 genes with decreased RPF abundance ($P < 0.05$, $| \log_2$ fold change $| > | 1 |$, n = 2) were identified in *Eef1g*-cKO leptotene and zygotene spermatocytes.

Table S5 Translational efficiency (TE) of control and *Eef1g*-cKO leptotene and zygotene spermatocytes. A total of 144 genes showed significantly increased TE (ΔTE Log_2 FC > 0,

adjusted $P < 0.05$), and 168 genes showed significantly decreased TE (ΔTE Log_2 FC < 0, adjusted $P < 0.05$).
