# Supplementary material for: eEF1G Orchestrates Translation to Ensure Meiotic Progression in Transcriptionally Quiescent Spermatocytes
Source: Adv Sci (Weinh). 2026 Jul 1:e76264. Online ahead of print. doi: 10.1002/advs.76264 (PMC13336642; doi:10.1002/advs.76264)
Supplement: Supplementary file 1 — Supporting File 1: advs76264‐sup‐0001‐SuppMat.pdf. [file ADVS-9999-e76264-s001.pdf]

Supporting Information

eEF1G orchestrates translation to ensure meiotic progression in transcriptionally quiescent spermatocytes

Jianze Xu[1,2,3,4,5,6†], Yuwei Hu[1,2,3,4,5,6,7†], Xukun Lu[1,2,3,4,5,6†], Yuling Cai[1,2,3,4,5,6,7†], Tongtong Li[1,2,3,4,5,6,7], Ming Gao[1,2,3,4,5,6], Jinlong Ma[1,2,3,4,5,6], Yuan Gao[1,2,3,4,5,6], Shangming Liu[7], Zi-Jiang Chen[1,2,3,4,5,6,7]*, Jing Meng[8]*, Hongbin Liu[1,2,3,4,5,6,7]*, Xiaohua Jiang[9]*.

†These authors contributed equally to this work

*Corresponding author

This PDF file includes:

Supplementary figures

Figure S1 - 16

Supplementary tables

Table S6 and S7

Description of Table S1 to S5 (Separate excel file)

Other Supplementary materials for this manuscript include the following:

Table S1 - S5

Supplementary figures

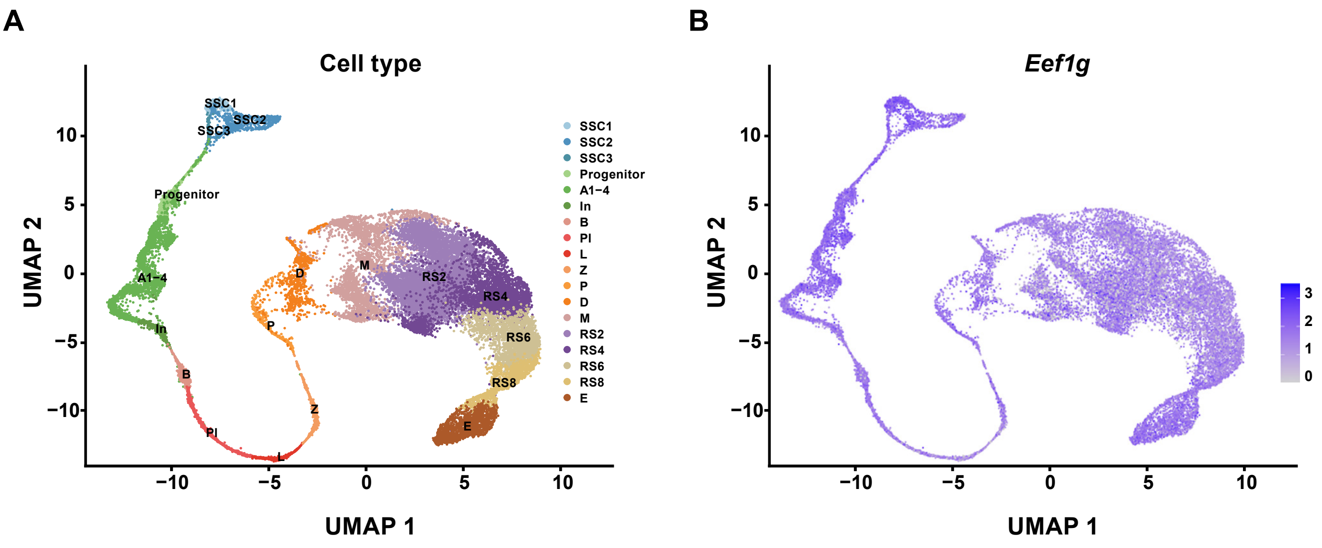

Figure S1. The expression pattern of *Eef1g* during spermatogenesis.

(A-B) t-SNE plots of mouse single-cell RNA sequencing (scRNA-seq) data showing *Eef1g* expression patterns. scRNA-seq datasets were obtained from the Male Health Atlas (http://malehealthatlas.cn/). SSC1, spermatogonial stem cell 1; SSC2, spermatogonial stem cell II; SSC3, A aligned spermatogonia III; Progenitor, spermatogonial progenitor cell; A1-4, A1-A4 spermatogonia; In, intermediate spermatogonia; B, type B spermatogonia; Pl, preleptotene; L, leptotene; Z, zygotene; P, pachytene; D, diplotene; M, metaphase; RS2, step1-2 round spermatid; RS4, step3-4 round spermatid; RS6, step5-6 round spermatid; RS8, step7-8 round spermatid; E, elongating spermatid.

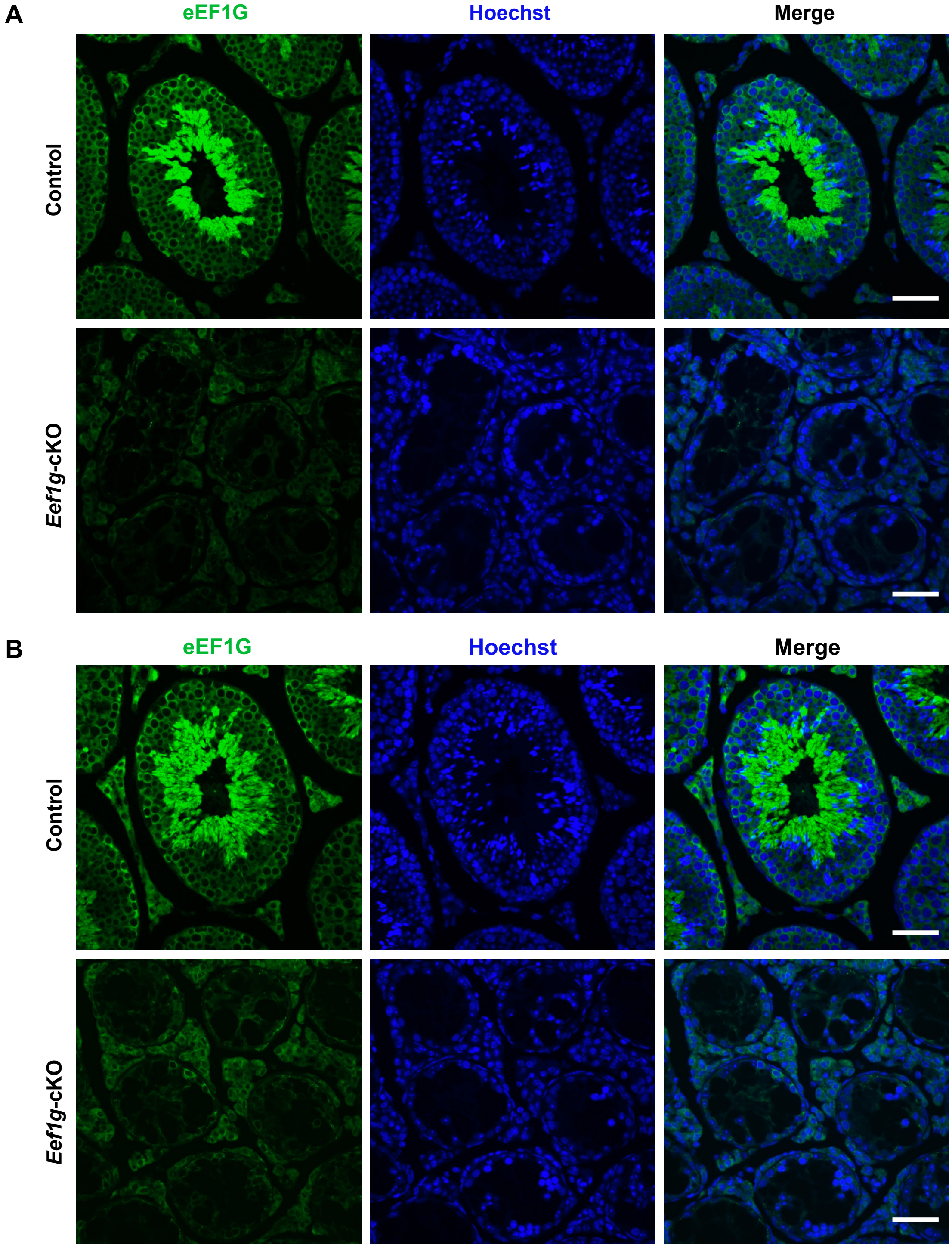

Figure S2. Deletion of *Eef1g* in germ cells.

(A) Immunofluorescence staining of eEF1G (antibody sourced from Abclonal) in mouse testis sections from control and *Eef1g*-cKO mice. Nuclei were stained with Hoechst (blue). Scale bars, 50 μm.

(B) Immunofluorescence staining of eEF1G (antibody sourced from Abmart) in mouse testis cross-sections from control and *Eef1g*-cKO mice. Nuclei were stained with Hoechst (blue). Scale bars, 50 μm.

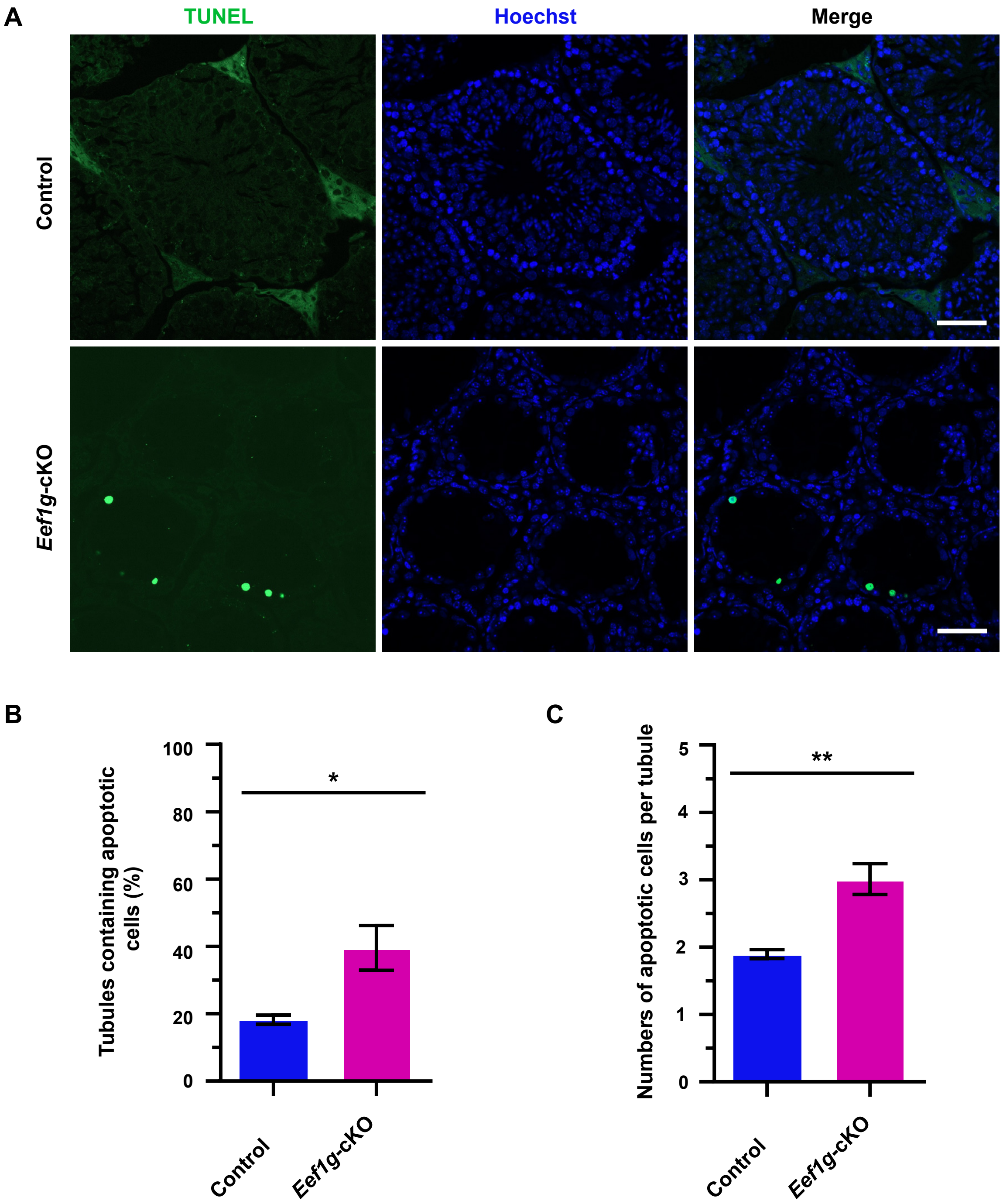

Figure S3. Germ cell-specific *Eef1g* ablation causes germ cell apoptosis.

(A) Testis sections from 10-week-old control and *Eef1g*-cKO mice were stained with TUNEL and Hoechst. Scale bars, 50 µm. **(B)** Percentage of TUNEL-positive tubules in testis sections from 10-week-old control and *Eef1g*-cKO mice (n = 4). **(C)** Quantification of TUNEL-positive cells per testicular tubule in 10-week-old control and *Eef1g*-cKO mice. Data are presented as the mean ± SEMs. *$P < 0.05$; **$P < 0.01$; Student's *t*-test.

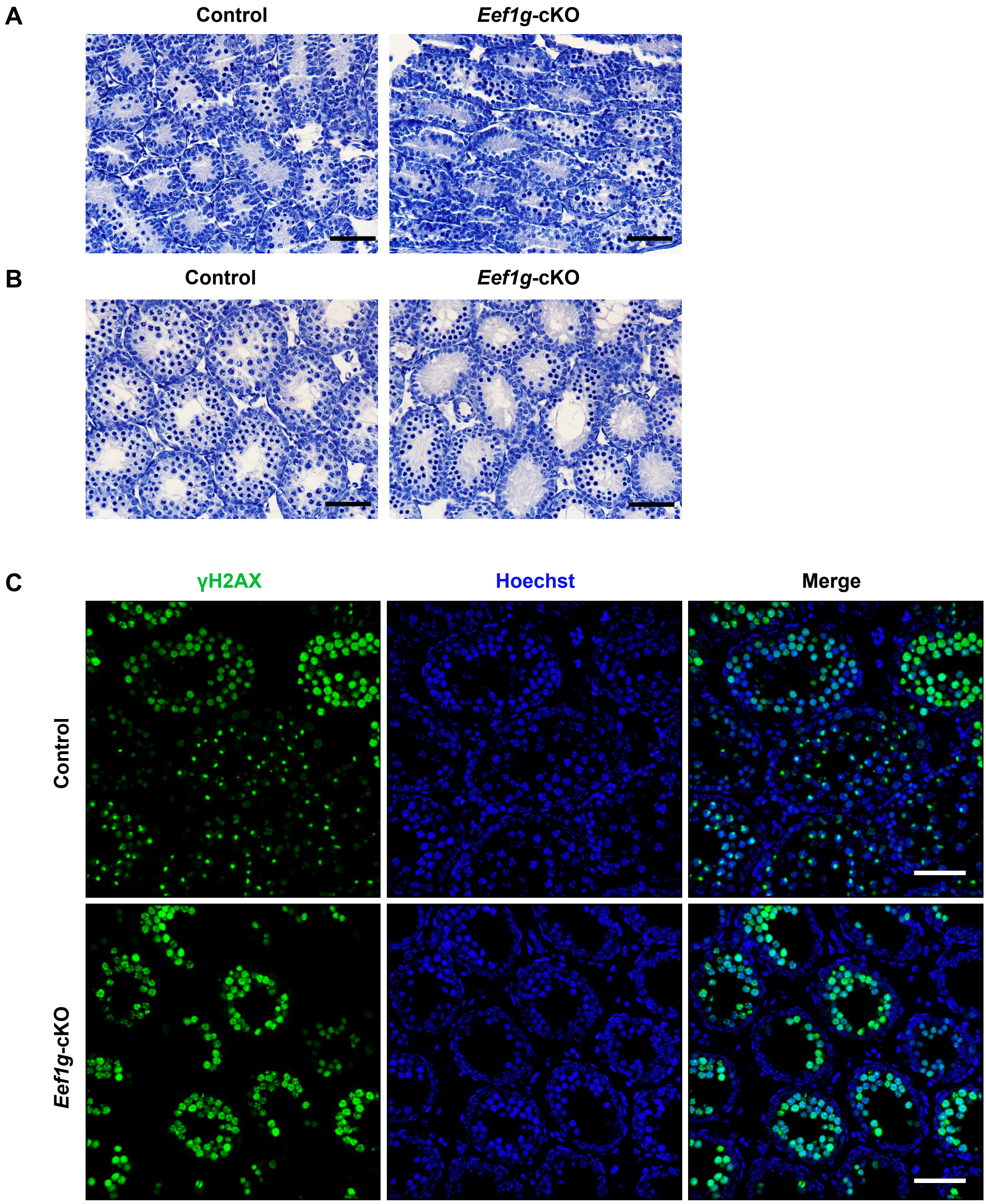

Figure S4. eEF1G deletion results in germ cell loss.

(A) Hematoxylin staining of testis sections from 10 dpp control and *Eef1g*-cKO mice. Scale bars, 50 µm. **(B)** Hematoxylin staining of testis sections from 14 dpp control and *Eef1g*-cKO mice. Scale bars, 50 µm. **(C)** Immunofluorescence staining of γH2AX in testis sections from 14 dpp control and *Eef1g*-cKO mice. Nuclei were stained with Hoechst (blue). Scale bars, 50 µm.

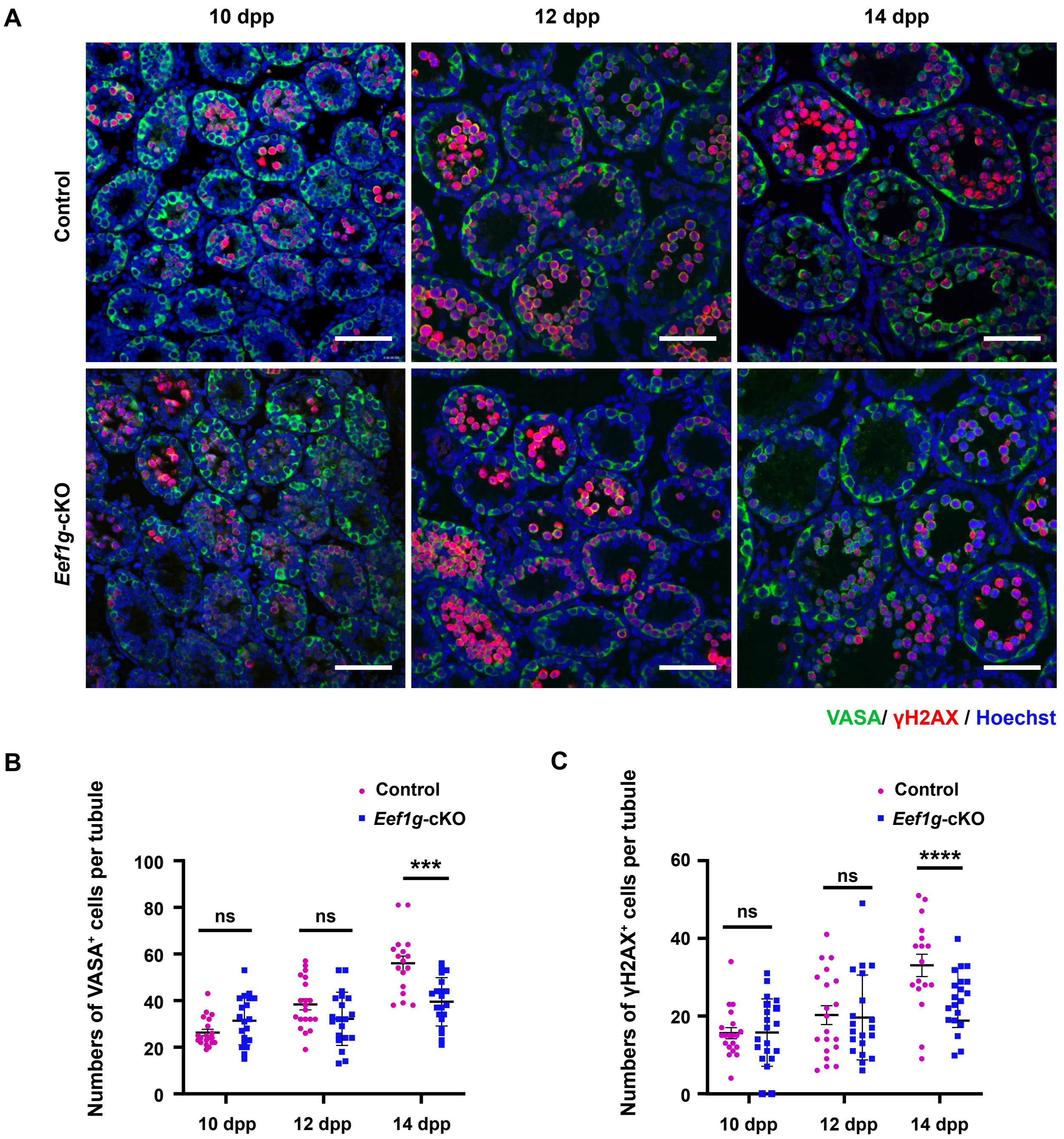

Figure S5. Spermatocytes decrease in the *Eef1g*-cKO mice after postnatal day 12.

(A) Immunostaining of VASA and γH2AX in testis sections from control and *Eef1g*-cKO mice at the indicated times. Nuclei were stained with Hoechst (blue). Scale bar, 50 µm. **(B)** Quantitative analysis of the number of VASA^{+} cells per tubule. Student's *t*-test. ***P < 0.001. ns, not statistically significant. Data are presented as means ± SEMs. **(C)** Quantitative analysis of the number of γH2AX^{+} cells per tubule. Student's *t*-test. ****P < 0.0001. ns, not statistically significant. Data are presented as means ± SEMs.

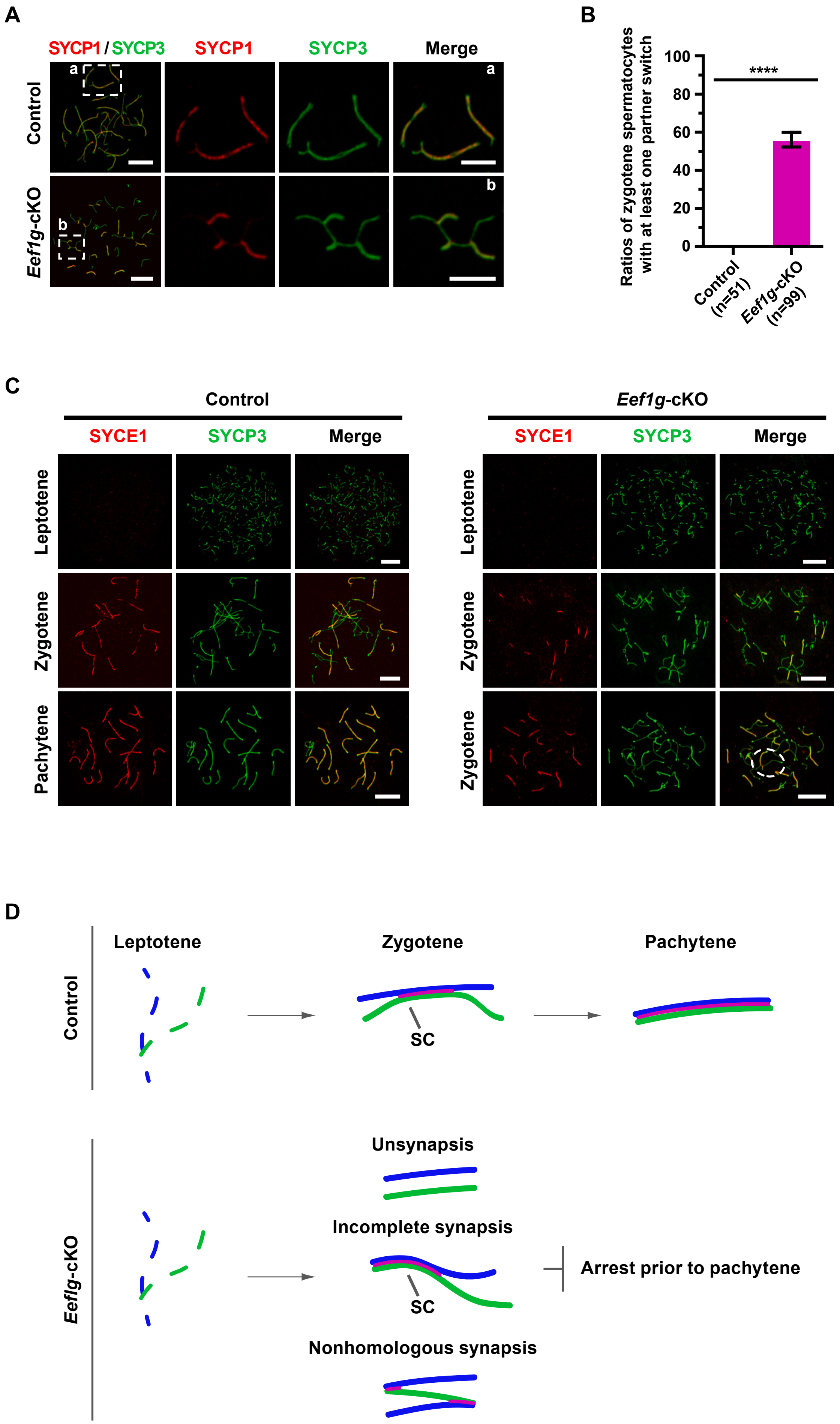
A
SYCP1/SYCP3
SYCP1
SYCP3
Merge
Control
Eef1g-cKO
a
b
B
Ratios of zygotene spermatocytes with at least one partner switch
100
80
60
40
20
0
****
Control (n=51)
Eef1g-cKO (n=99)
C
Control
Eef1g-cKO
SYCE1
SYCP3
Merge
Leptotene
Zygotene
Pachytene
D
Leptotene
Zygotene
Pachytene
Control
SC
Eeflg-cKO
Unsynapsis
Incomplete synapsis
SC
Arrest prior to pachytene
Nonhomologous synapsis

Figure S6. ***Eef1g*-deficient spermatocytes show synaptic defects.**

(A) Representative zygotene spermatocytes from control and *Eef1g*-cKO mice stained with antibodies for SYCP1 (red) and SYCP3 (green). Magnified pictures highlight the partner switch in *Eef1g*-cKO spermatocytes. Scale bars, 10 μm (main panels) and 5 μm (insets). **(B)** The percentage of zygotene spermatocytes with at least one partner switch is shown (control, n = 51; *Eef1g*-cKO, n = 99; pooled from three mice per genotype). Scale bars: 10 μm (main panel), 5 μm (insets). 56.06 ± 3.82% (mean ± SEM) of *Eef1g*-cKO zygotene spermatocytes showed at least one partner switch. Unpaired two-tailed Student's *t*-test. ****$P < 0.0001$. **(C)** Representative images of spermatocyte spreads from 10-week-old control and *Eef1g*-cKO mice stained for SYCE1 (red) and SYCP3 (green). Scale bars: 10 μm. **(D)** Schematic summary of the synapsis defects in *Eef1g*-cKO spermatocytes. *Eef1g*-deficient spermatocytes exhibit synapsis defects such as asynapsis (completely separated chromosomes), incomplete synapsis, and aberrant non-homologous synapsis.

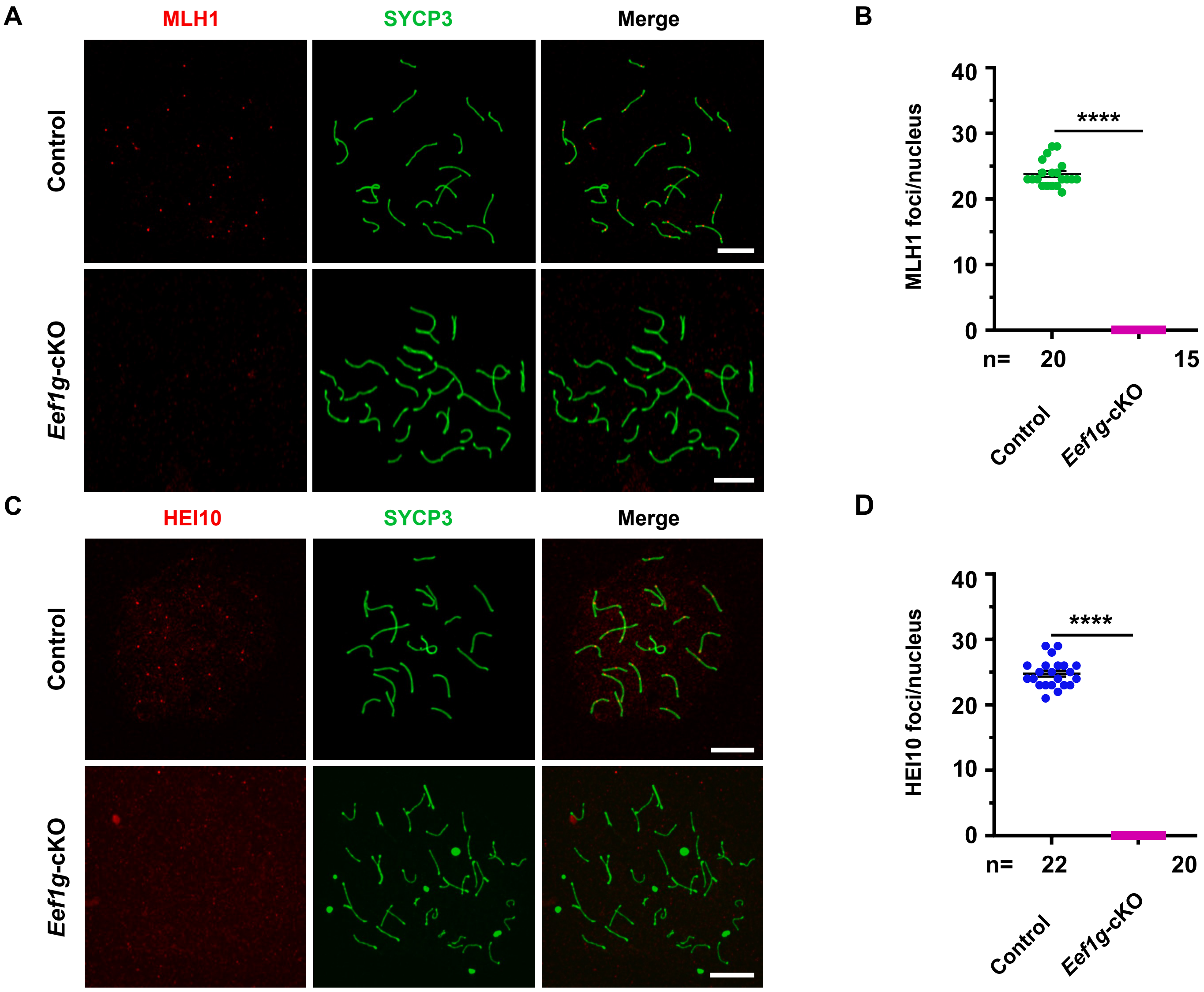

Figure S7. ***Eef1g*** **deletion in germ cells causes defects in meiotic recombination.**

(A) Representative spermatocyte spreads from adult control and *Eef1g*-cKO mice stained for SYCP3 (green) and MLH1 (red). Scale bars: 10 μm. **(B)** Quantification of MLH1 foci in spermatocytes. n, the total number of cells analyzed from two animals per genotype. The bars represent mean ± SEMs. ****$P < 0.0001$; Student' s *t*-test. **(C)** Representative spreads of spermatocytes from adult control and *Eef1g*-cKO mice stained for SYCP3 (green) and HEI10 (red). Scale bars: 10 μm. **(D)** Quantification of HEI10 foci in spermatocytes. n, the total number of cells analyzed from three animals per genotype. The bars represent mean ± SEMs. ****$P < 0.0001$; Student's *t*-test.

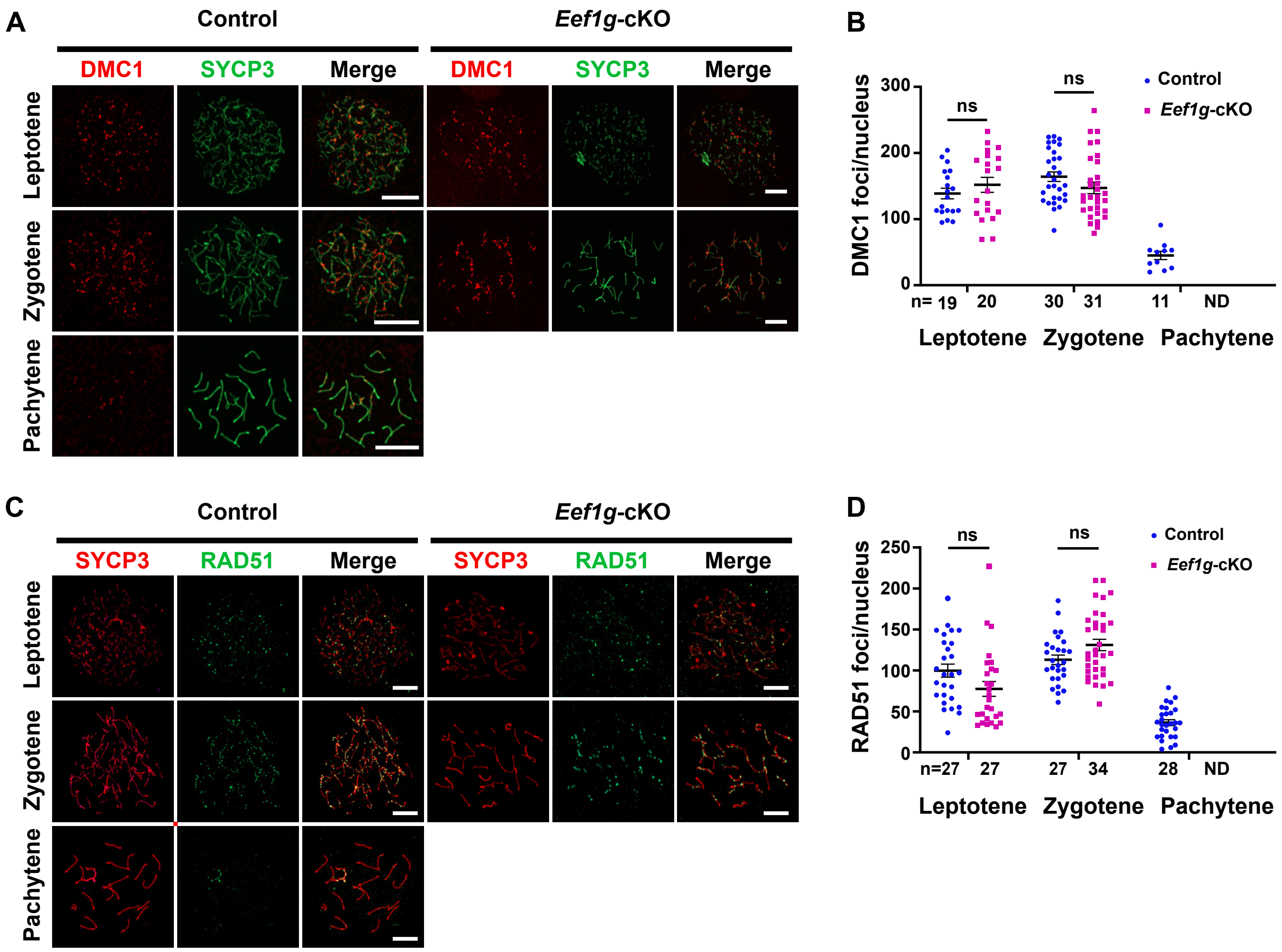

Figure S8.DMC1 and RAD51 signals remain normal in *Eef1g*-deficient spermatocytes.

(A) Representative spread spermatocytes from adult control and *Eef1g*-cKO mice were immunostained for SYCP3 and DMC1. Leptotene, zygotene, and pachytene spermatocytes are shown. Scale bars, 10 μm. **(B)** Quantification of DMC1 foci in spread spermatocytes at the indicated substages. n, the total number of cells analyzed from three animals per genotype. The bars indicate the mean ± SEMs. P values were calculated by the Student' s *t*-test. ns, not significant ($P > 0.05$). **(C)** Representative spread spermatocytes from adult control and *Eef1g*-cKO mice were immunostained for SYCP3 and RAD51. Leptotene, zygotene, and pachytene spermatocytes are shown. Scale bars, 10 μm. **(D)** Quantification of RAD51 foci in spread spermatocytes at the indicated substages. n, the total number of cells analyzed from three animals per genotype. The bars indicate the mean ± SEMs. P values were calculated by the Student' s *t*-test. ns, not significant ($P > 0.05$).

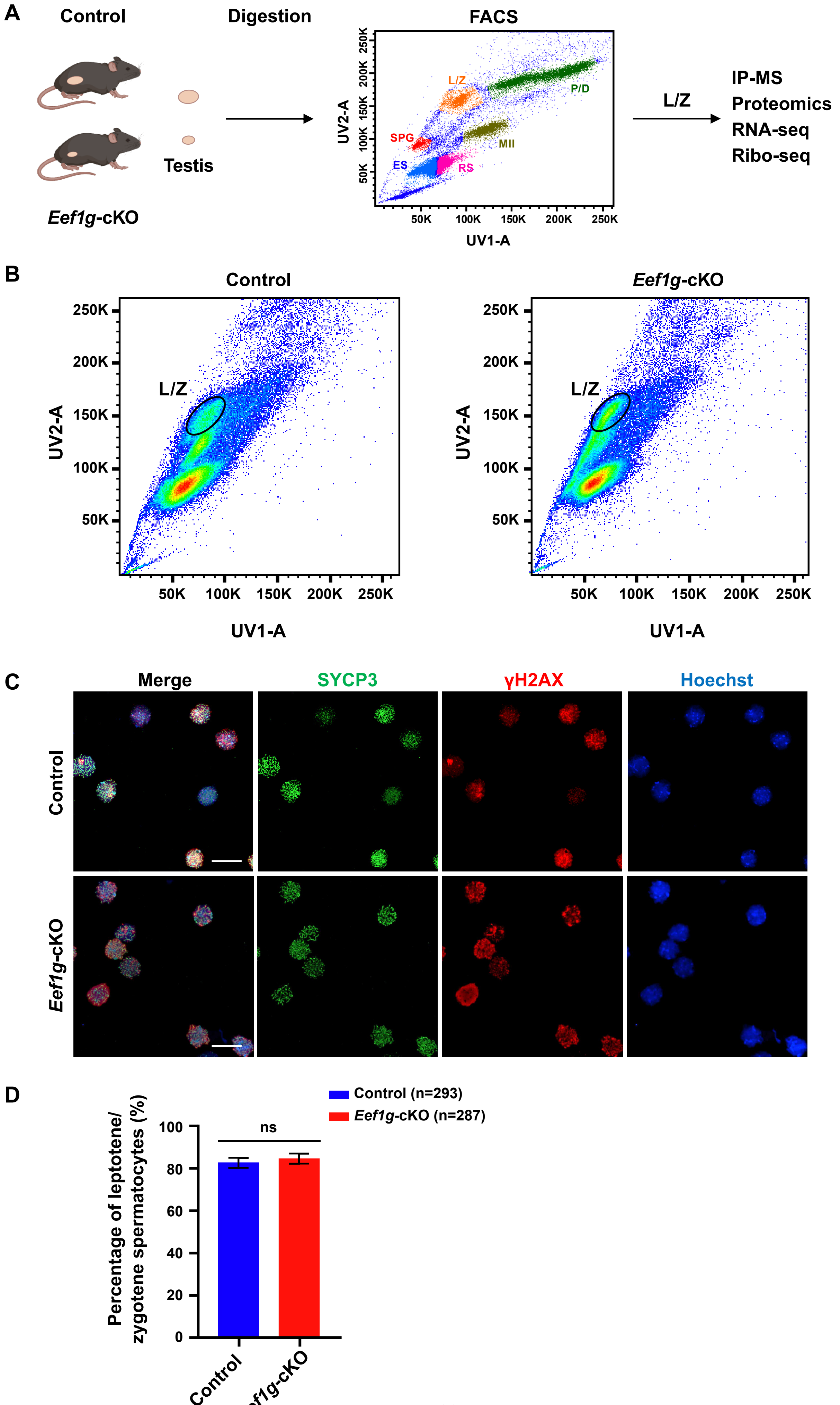
A
Control
Digestion
FACS
Testis
Eef1g-cKO
UV2-A
UV1-A
50K 100K 150K 200K 250K
L/Z
P/D
SPG
MII
ES
RS
L/Z
IP-MS
Proteomics
RNA-seq
Ribo-seq
B
Control
Eef1g-cKO
L/Z
UV2-A
UV1-A
250K 200K 150K 100K 50K
50K 100K 150K 200K 250K
C
Merge
SYCP3
γH2AX
Hoechst
Control
Eef1g-cKO
D
Control (n=293)
Eef1g-cKO (n=287)
ns
Percentage of leptotene/ zygotene spermatocytes (%)
100 80 60 40 20 0
Control
Eef1g-cKO

Figure S9. Isolation of leptotene/zygotene spermatocytes from 12 dpp mouse testes via FACS.

(A) Schematic illustration of eEF1G immunoprecipitation-mass spectrometry (IP-MS), proteome analysis, RNA sequence (RNA-seq), and ribosome sequencing (Ribo-seq) experiments using leptotene and zygotene spermatocytes isolated via fluorescence-activated cell sorting (FACS). **(B)** Flow cytometry analysis of the spermatogenic cell population in 12 dpp control and *Eef1g*-cKO testes. L/Z indicates leptotene/zygotene spermatocytes. **(C)** FACS-purified leptotene/zygotene spermatocytes from 12 dpp control and *Eef1g*-cKO testes were stained for SYCP3 (green) and γH2AX (red). Nuclei were stained with Hoechst (blue). Scale bar: 20 μm. **(D)** Percentage of leptotene/zygotene spermatocytes in FACS-purified populations from control and *Eef1g*-cKO testes. Student's *t*-test; ns,not significant ($P >$ 0.05). Data represent mean ± SEMs (n = 3).

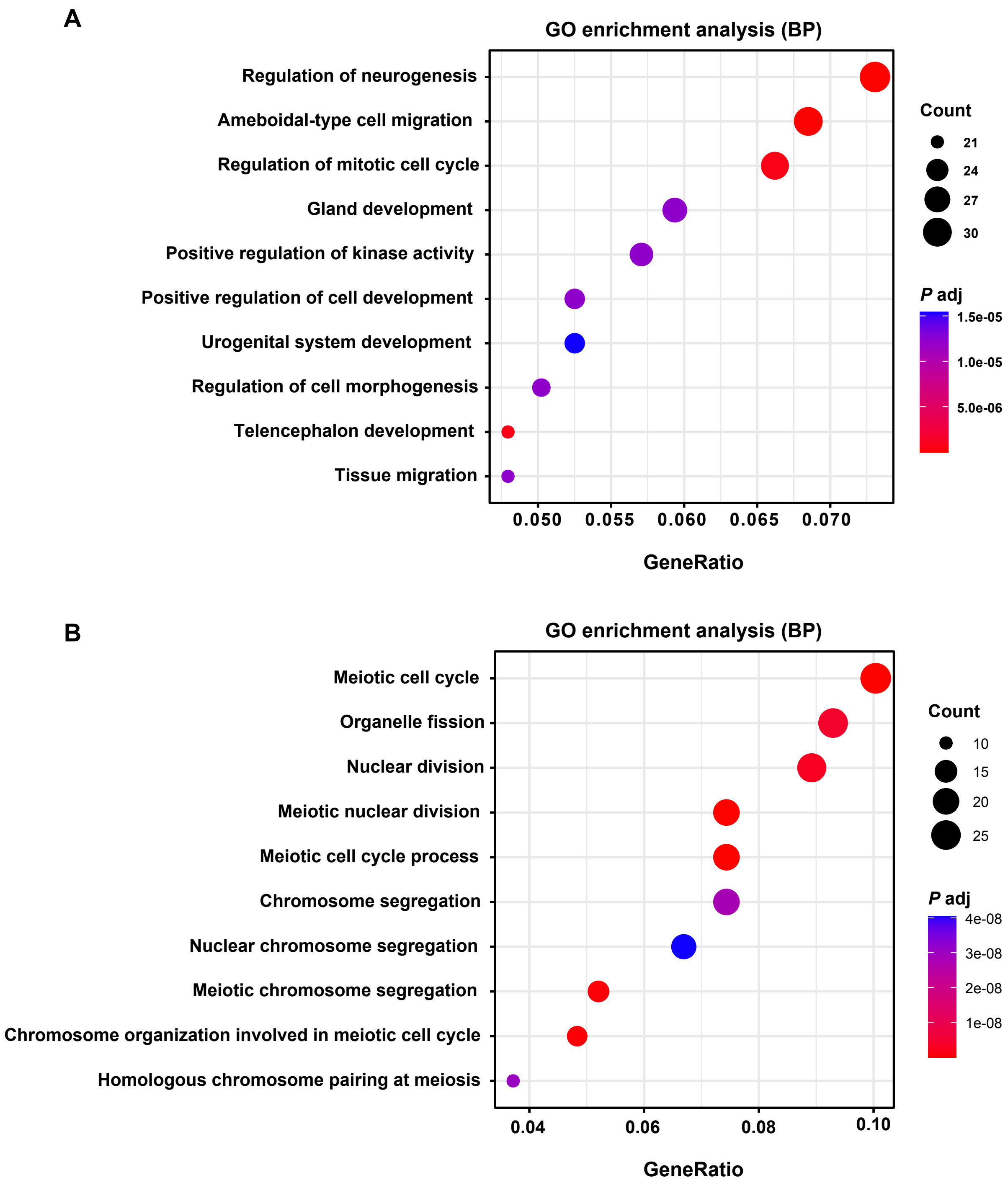

Figure S10. GO analysis of transcriptionally altered genes in *Eef1g*-cKO spermatocytes. **(A)** Gene ontology enrichment analysis of downregulated genes in *Eef1g*-deficient leptotene and zygotene spermatocytes. **(B)** Gene ontology enrichment analysis of upregulated genes in *Eef1g*-deficient leptotene and zygotene spermatocytes.

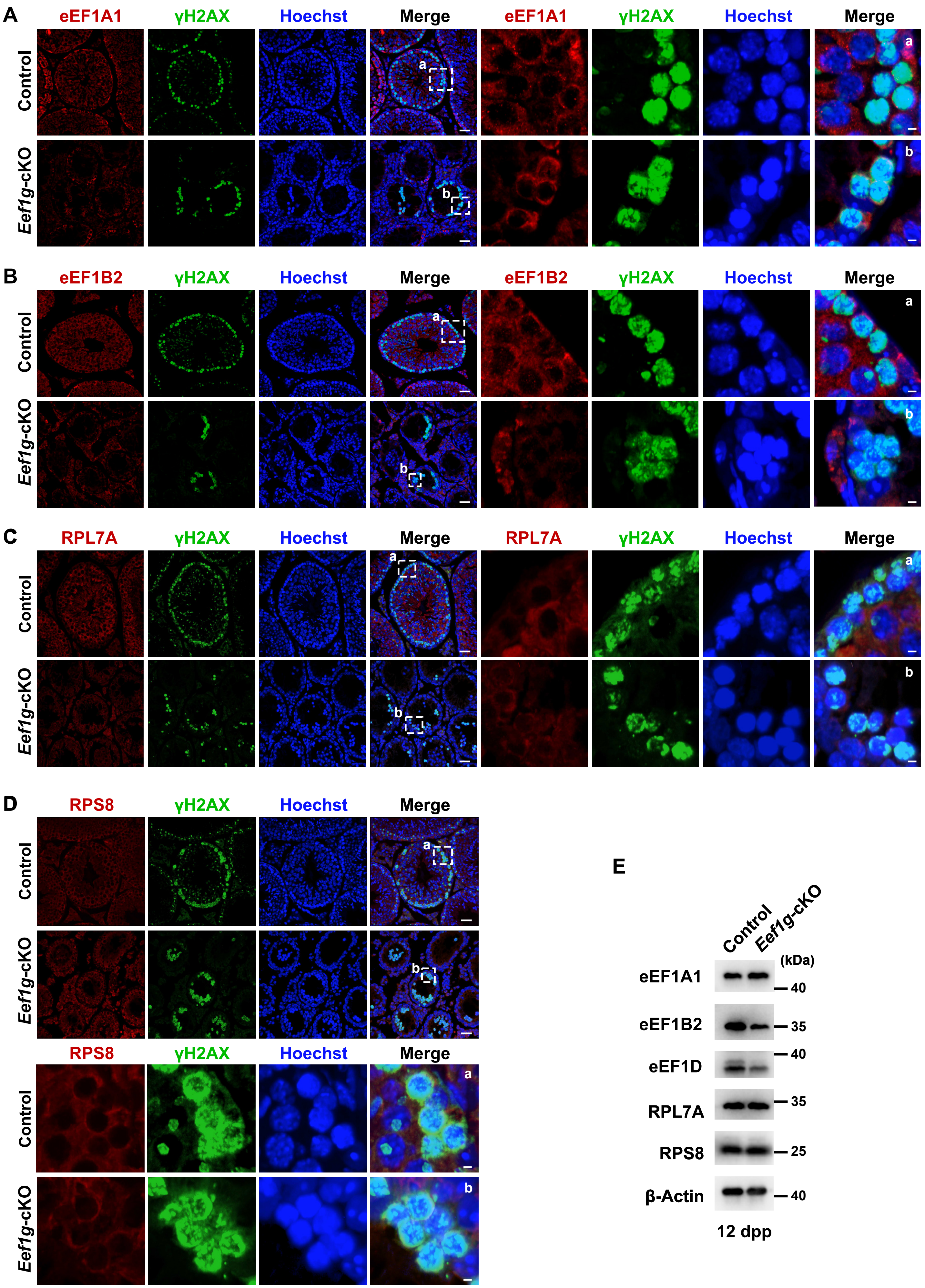
A
eEF1A1
γH2AX
Hoechst
Merge
Control
Eef1g-cKO
a
b
B
eEF1B2
C
RPL7A
D
RPS8
E
Control
Eef1g-cKO
(kDa)
eEF1A1
40
eEF1B2
35
eEF1D
40
RPL7A
35
RPS8
25
β-Actin
40
12 dpp

Figure S11. Germ cell-specific *Eef1g* deletion reduces eEF1B2 and eEF1D protein levels.

(A) Immunofluorescence staining of eEF1A1 (red) and γH2AX (green) in mouse testis sections from 10-week-old control and *Eef1g*-cKO mice. Nuclei were stained with Hoechst (blue). Scale bars, 20 μm (main panels) and 3 μm (insets). **(B)** Immunofluorescence staining of eEF1B2 (red) and γH2AX (green) in mouse testis sections from 10-week-old control and *Eef1g*-cKO mice. Nuclei were stained with Hoechst (blue). Scale bars, 20 μm (main panels) and 3 μm (insets). **(C)** Immunofluorescence staining of RPL7A (red) and γH2AX (green) in mouse testis sections from 10-week-old control and *Eef1g*-cKO mice. Nuclei were stained with Hoechst (blue). Scale bars, 20 μm (main panels) and 3 μm (insets). **(D)** Immunofluorescence staining of RPS8 (red) and γH2AX (green) in mouse testis sections from 10-week-old control and *Eef1g*-cKO mice. Nuclei were stained with Hoechst (blue). Scale bars, 20 μm (main panels) and 3 μm (insets). **(E)** Representative Western blot showing the protein levels of eEF1A1, eEF1B2, eEF1D, RPL7A, and RPS8 in testes of 12 dpp *Eef1g*-cKO and control mice. β-Actin was used as a loading control.

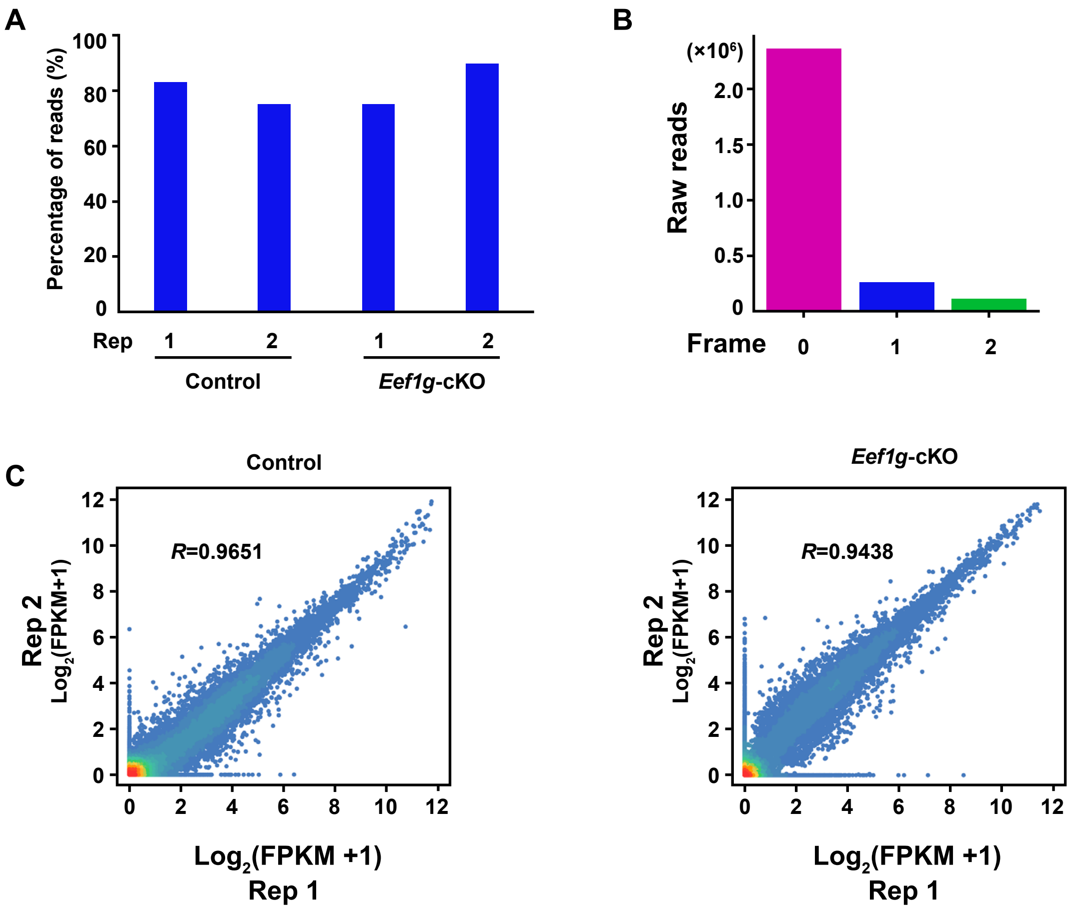

Figure S12. Results of quality control (QC).

(A) The percentage of reads exhibiting 3-nucleotide periodicity across different groups. **(B)** Reads mapped to distinct reading frames. Representative sample (Control Rep1) shown; all four replicates consistent (frame 0 enrichment). **(C)** Scatter plots for comparing the Ribo-seq data generated in this study, with Spearman correlation coefficients indicated.

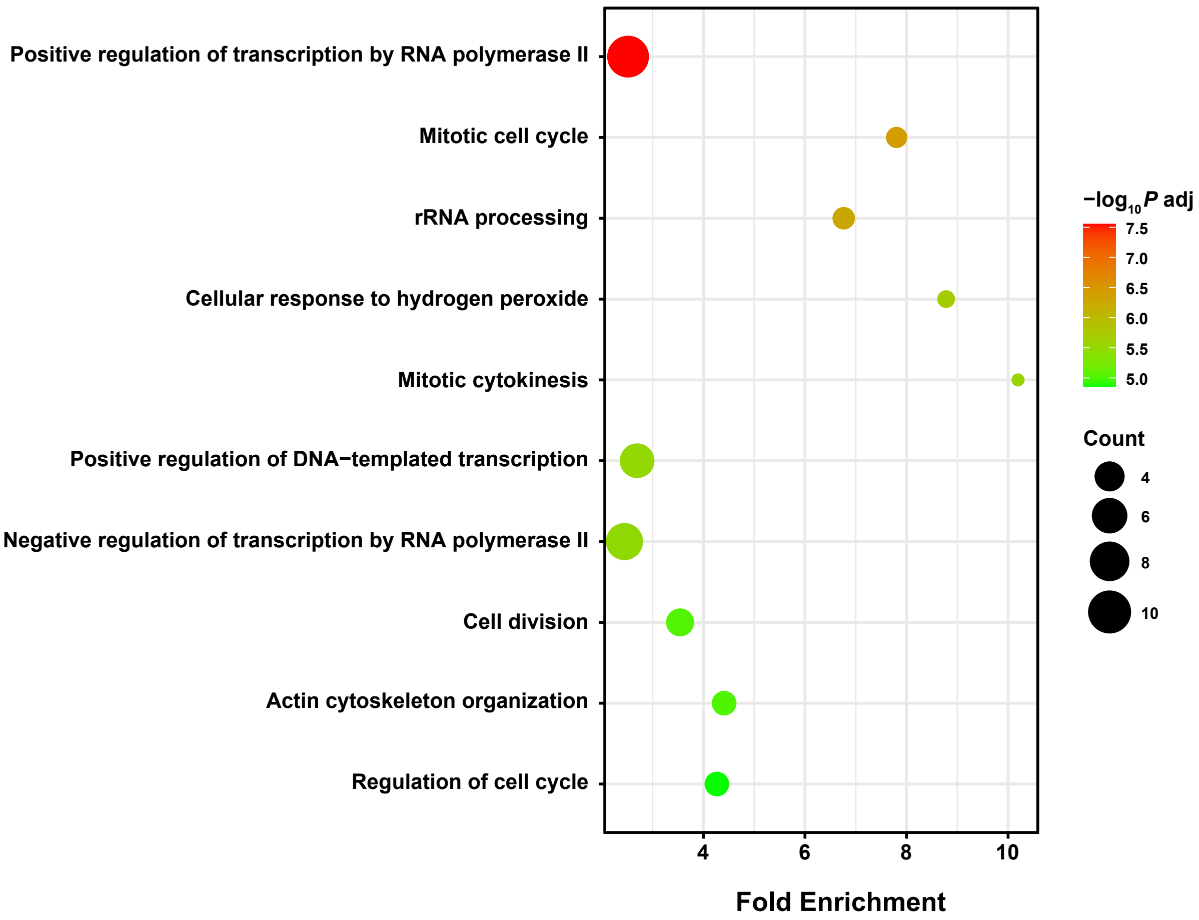

Figure S13. GO enrichment analysis of downregulated ribosome-protected fragments (RPFs) in *Eef1g*-cKO spermatocytes.

GO analysis of downregulated ribosome-protected fragments (RPFs) in leptotene and zygotene spermatocytes from *Eef1g*-cKO mice.

A

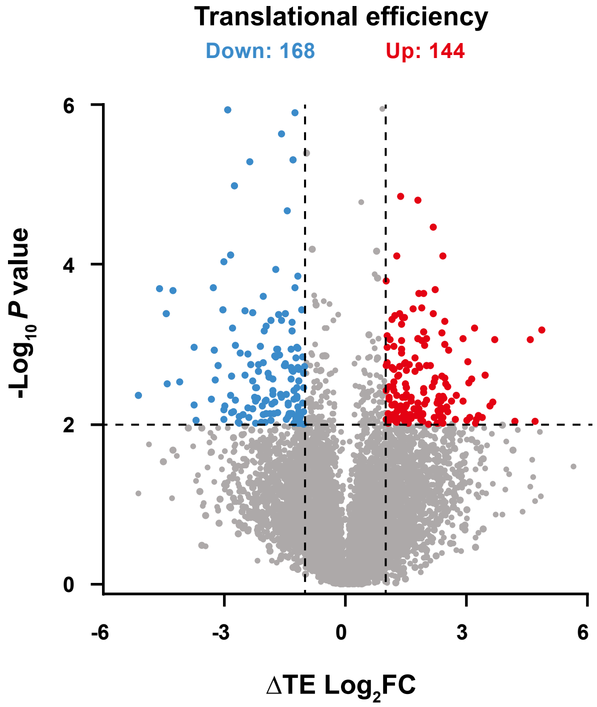
Translational efficiency
Down: 168
Up: 144
-Log10 P value
0
2
4
6
-6
-3
0
3
6
ΔTE Log2FC

B

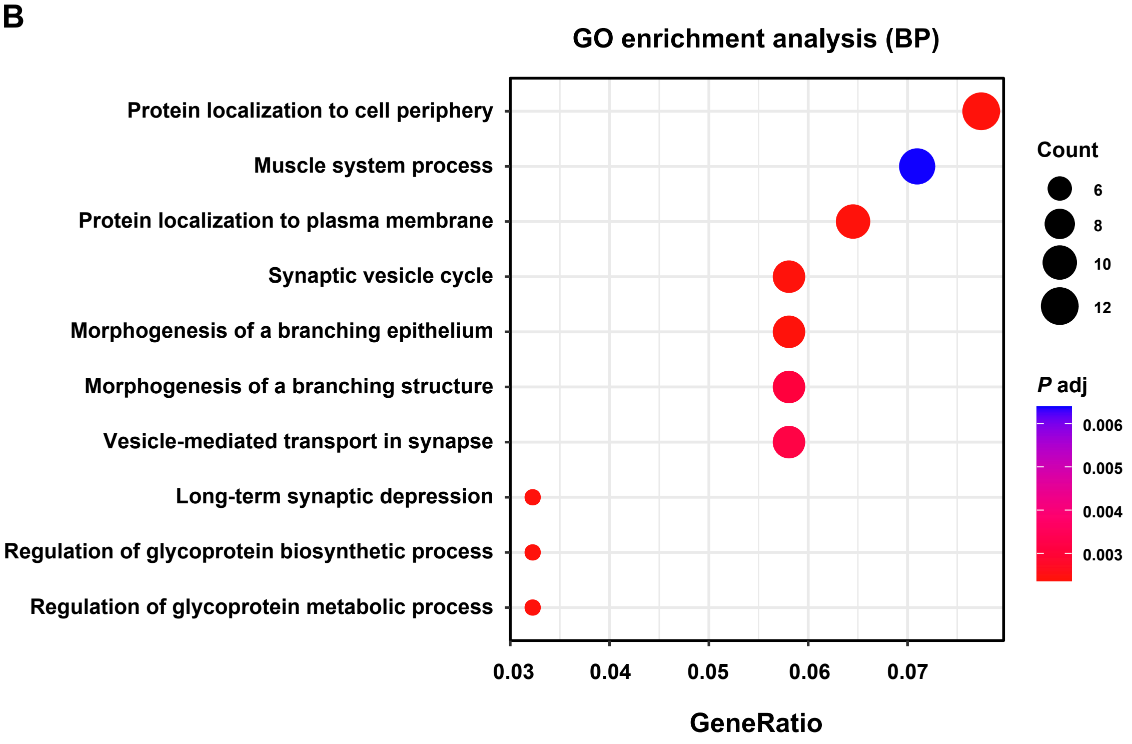
GO enrichment analysis (BP)
Protein localization to cell periphery
Muscle system process
Protein localization to plasma membrane
Synaptic vesicle cycle
Morphogenesis of a branching epithelium
Morphogenesis of a branching structure
Vesicle-mediated transport in synapse
Long-term synaptic depression
Regulation of glycoprotein biosynthetic process
Regulation of glycoprotein metabolic process
0.03
0.04
0.05
0.06
0.07
GeneRatio
Count
6
8
10
12
P adj
0.006
0.005
0.004
0.003

Figure S14. Translational efficiency changes in *Eef1g*-cKO spermatocytes are not enriched for meiotic genes.

(A) Volcano plot showing changes in translational efficiency (ΔTE) between *Eef1g*-cKO and control spermatocytes. The x axis represents the $\log_2$ fold change in translational efficiency (Δ TE Log_2FC), and the y axis represents the $-\log_{10} P$ value. Genes with significantly increased translational efficiency (ΔTE > 0) are labeled as " Up " (n = 144), and those with significantly decreased translational efficiency (ΔTE < 0) are labeled as " Down " (n = 168). The dashed line indicates the significance threshold. **(B)** GO enrichment analysis of genes with increased translational efficiency (ΔTE > 0, n = 144) in *Eef1g*-cKO spermatocytes was compared to control spermatocytes. The dot plot shows selected significantly enriched biological processes, ordered by GeneCount (number of genes in each term). Note: No significant GO terms were enriched among genes with decreased translational efficiency (ΔTE < 0, n = 168)

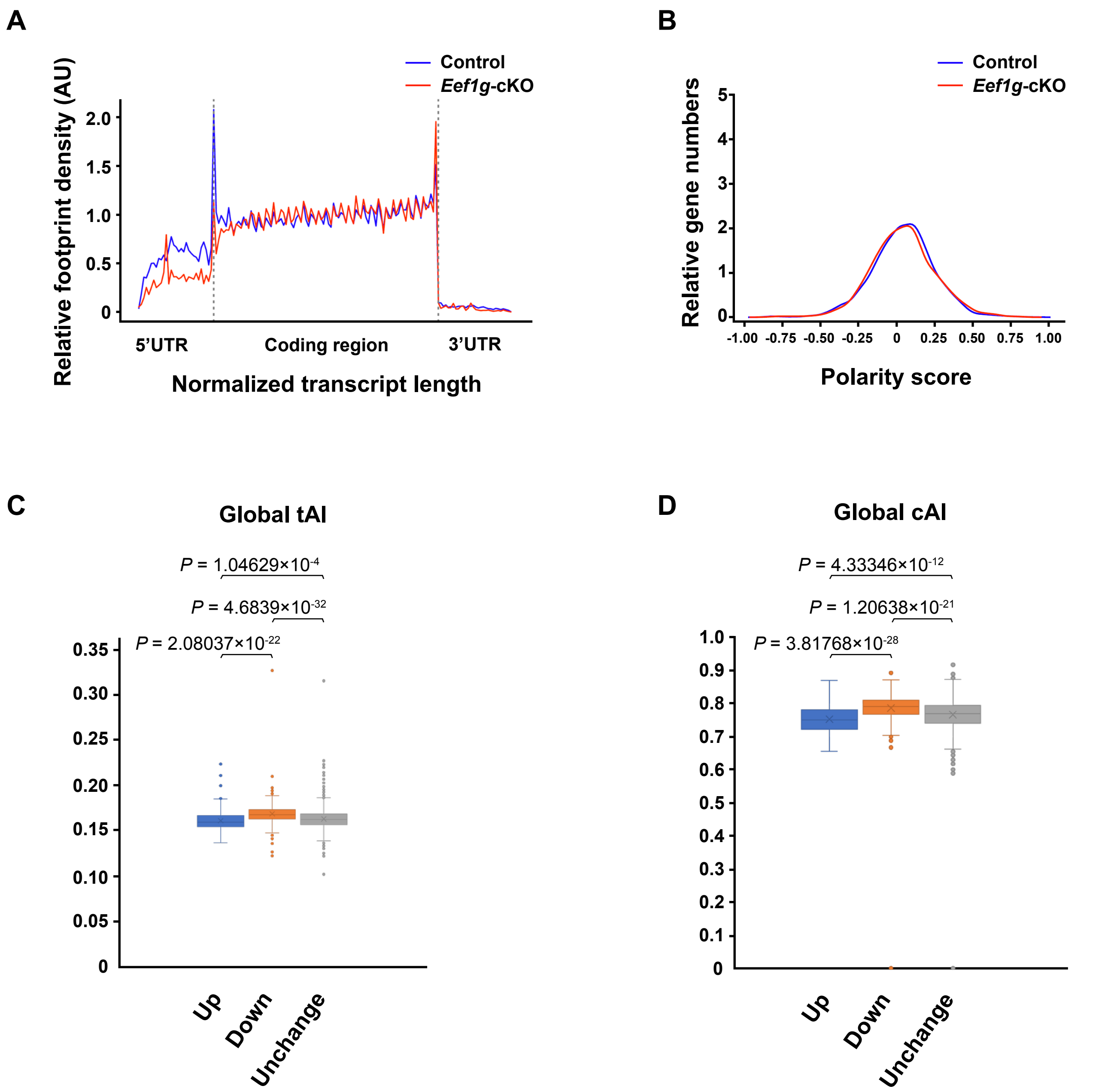

Figure S15. Ribo-seq analysis of translation features in control and *Eef1g*-deficient spermatocytes.

(A) The global distribution pattern of reads along transcripts between control and *Eef1g*-cKO spermatocytes. **(B)** Distribution of polarity scores between control and *Eef1g*-cKO spermatocytes. **(C)** Distribution of the global tRNA adaptation index (tAI) across different gene sets. *P* values were calculated using a two-tailed Student' s *t*-test and are indicated on the graphs. **(D)** Distribution of the global codon adaptation index (cAI) across different gene sets. *P* values were calculated using a two-tailed Student's *t*-test and are indicated on the graphs.

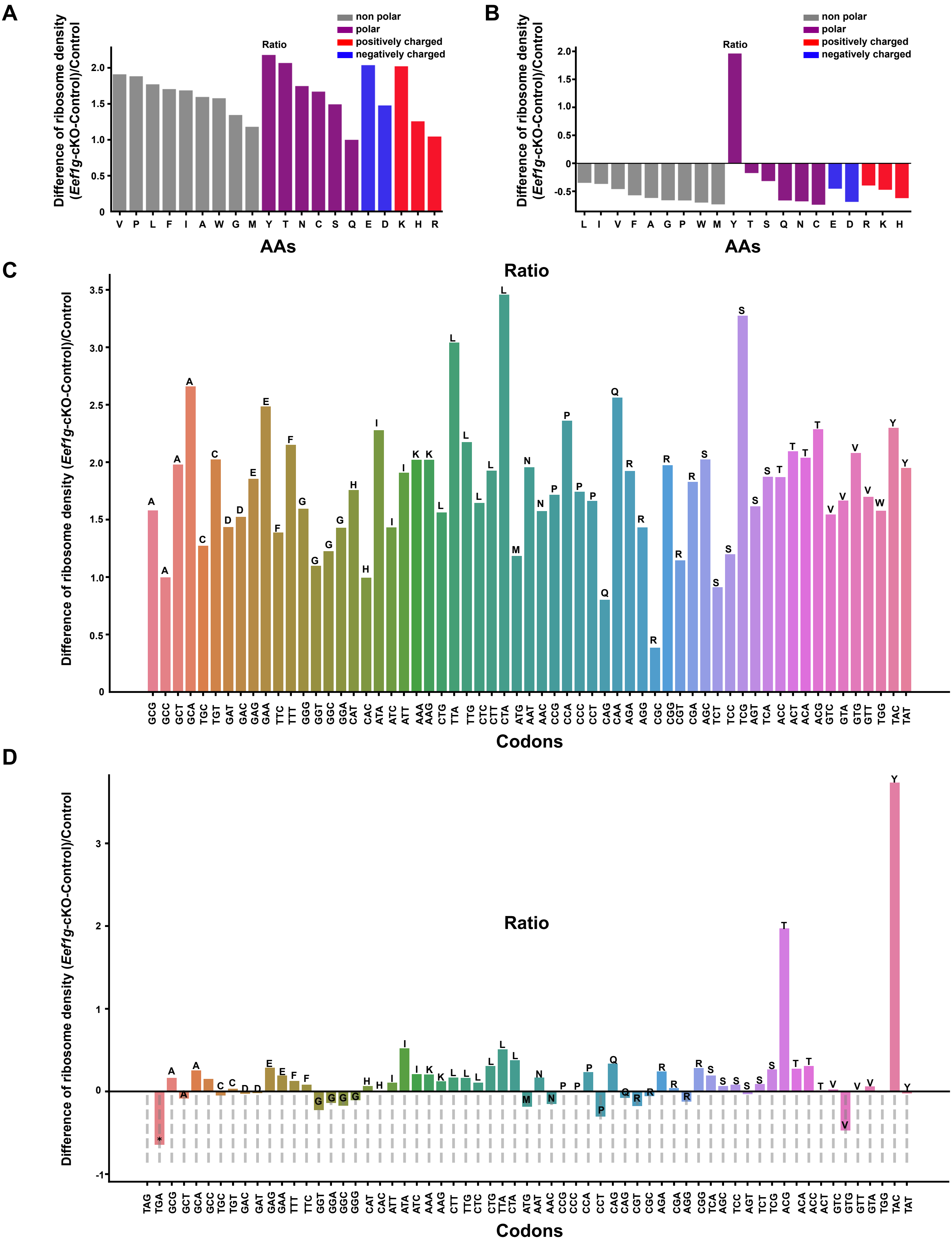
A
B
C
D
non polar
polar
positively charged
negatively charged
Ratio
Difference of ribosome density (Eef1g-cKO-Control)/Control
AAs
Codons

Figure S16. Ribosome density across different amino acids and codons.

(A) Fold change in ribosome density between *Eef1g*-cKO samples and control samples for different amino acids within upregulated gene sets. **(B)** Fold change in ribosome density between *Eef1g*-cKO samples and control samples for different amino acids within downregulated gene sets. **(C)** Fold change in ribosome density between *Eef1g*-cKO samples and control samples for codons within up-regulated gene sets. **(D)** Fold change in ribosome density between *Eef1g*-cKO samples and control samples for codons within downregulated gene sets.

Supplementary tables

Table S6 Primers used in this study.

Primer names	Sequences (5’-3’)
Eef1g-5wt-tF1	AGGGTTGCTAATTCGCCATG
Eef1g-3wt-tR1A	GTAGGTGGGTCAAAACTGAGCC
Eef1g-3wt-tR1	TGACCAGGCTAAATGAGTGAGTG
Stra8-GFPcre–F	ACTCCAAGCACTGGGCAGAA
Stra8-GFPcre-R1	GCCACCATAGCAGCATCAAA
Stra8-GFPcre-R2	CGTTTACGTCGCCGTCCAG

Table S7 Antibodies used in this study

Antibodies	Sources	Identifiers
Anti-SYCP3 Antibody	Abcam	Cat# Ab97672<br>RRID: AB_10678841
Anti-SYCP1 Antibody	Abcam	Cat# Ab15090<br>RRID: AB_301636
Anti-SYCP1 Antibody	Novus Biologicals	Cat# NB300-229<br>RRID: AB_10002742
Anti-β-Actin Antibody	Proteintech	Cat# 66009-1-Ig<br>RRID: AB_2687938
Anti-GAPDH Antibody	Proteintech	Cat# 60004-1-Ig<br>RRID: AB_2107436
Anti-RPL4 Antibody	Proteintech	Cat# 67028-1-Ig<br>RRID: AB_2882343
Anti-DDX4 Antibody	Abcam	Cat# ab27591<br>RRID: AB_11139638
Anti-RPS8 Antibody	Proteintech	Cat# 18228-1-AP<br>RRID: AB_10666168
Anti-RPL7A Antibody	Proteintech	Cat# 15340-1-AP<br>RRID: AB_2254051

Anti-EEF1A1 Antibody	Proteintech	Cat# 81377-1-RR<br>RRID: AB_2935549
Anti-EEF1B2 Antibody	Abclonal	Cat# A6580<br>RRID: AB_2863531
Anti-EEF1D Antibody	Abclonal	Cat# A2509<br>RRID: AB_2764400
Anti-EEF1G antibody	Abclonal	Cat# A8744<br>RRID: AB_2863600
Anti-EEF1G antibody	Abmart	Cat# T59970M
Anti-gamma H2A.X (phospho S139)	Abcam	Cat# Ab81299<br>RRID: AB_1640564
Anti-p-Histone H2A.X (Ser139) Antibody	Merck	Cat# 05-636<br>RRID: AB_309864
Anti-SYCE1 Polyclonal antibody	Proteintech	Cat# 11063-1-AP<br>RRID: AB_2197057
Anti-MLH1 Recombinant antibody	Proteintech	Cat# 11697-1-AP<br>RRID: AB_2145604
Anti-TEX12 Polyclonal antibody	Proteintech	Cat# 17068-1-AP<br>RRID: AB_2201875
Anti-HIST1H1T Polyclonal antibody	Proteintech	Cat# 18188-1-AP<br>RRID: AB_2878515
Anti-RAD51 Polyclonal antibody	Proteintech	Cat# 14961-1-AP<br>RRID: AB_2177083
Anti-DMC1 Polyclonal antibody	Proteintech	Cat# 13714-1-AP<br>RRID: AB_2091076
Anti-HORMAD1 Polyclonal antibody	Proteintech	Cat# 28719-1-AP<br>RRID: AB_2881199
Anti-MSH4 antibody	Abclonal	Cat# A8556<br>RRID: AB_2770446
Anti-SHOC1 antibody	From Professor Chao Yu	
Anti-RNF212 antibody	From Professor Mengcheng Luo	
Anti-HEI10 antibody	Home-made	
Anti-REC8 antibody	Abcam	Cat# ab192241

Anti-HSPA2 antibody	Proteintech	Cat# 12797-1-AP<br>RRID: AB_2119687
Anti-ATR antibody	Proteintech	Cat# 19787-1-AP<br>RRID:AB_10639516
Anti-TEX11 Polyclonal antibody	Proteintech	Cat# 17266-1-AP<br>RRID: AB_2201874
Lectin PNA From Arachis hypogaea (peanut), Alexa Fluor™ 594 Conjugate	Thermo Fisher Scientific	Cat# L32459

Description of Table S1-S5.

Table S1 Proteomics analysis of control and *Eef1g*-cKO leptotene and zygotene spermatocytes. Proteomic analyses identified 1,001 differentially expressed proteins ($P < 0.05$, |$\log_2$ fold change| > |0.585|, n = 3), including 89 upregulated and 912 downregulated proteins

Table S2 RNA sequencing (RNA-seq) analysis of control and *Eef1g*-cKO leptotene and zygotene spermatocytes. 303 genes were upregulated and 488 genes were downregulated ($P <$ 0.05, |$\log_2$ fold change| > |1|, n = 3).

Table S3 Proteins that interact with eEF1G detected by IP-MS in WT leptotene and zygotene spermatocytes. MS results identified 58 eEF1G-interacting proteins, among which 31 were ribosomal proteins (including 27 large ribosomal subunits and 4 small ribosomal subunits), and 27 were RNA-binding proteins.

Table S4 Ribosome profiling (Ribo-seq) of control and *Eef1g*-cKO leptotene and zygotene spermatocytes. 502 genes with increased and 451 genes with decreased RPF abundance ($P < 0.05$, | $\log_2$ fold change | > | 1 |, n = 2) were identified in *Eef1g*-cKO leptotene and zygotene spermatocytes.

Table S5 Translational efficiency (TE) of control and *Eef1g*-cKO leptotene and zygotene spermatocytes. A total of 144 genes showed significantly increased TE (ΔTE Log_2 FC > 0,

adjusted $P < 0.05$), and 168 genes showed significantly decreased TE (ΔTE Log_2 FC < 0, adjusted $P < 0.05$).
